# Characterization of Rheumatoid Arthritis Risk-Associated SNPs and Identification of Novel Therapeutic Sites Using an *In-Silico* Approach

**DOI:** 10.3390/biology10060501

**Published:** 2021-06-04

**Authors:** Mehran Akhtar, Yasir Ali, Zia-ul Islam, Maria Arshad, Mamoona Rauf, Muhammad Ali, Saleh N. Maodaa, Saleh A. Al-Farraj, Hamed A. El-Serehy, Fazal Jalil

**Affiliations:** 1 Department of Biotechnology, Garden Campus, Abdul Wali Khan University Mardan, Khyber Pakhtunkhwa 23200, Pakistan; mehranakhtar94@yahoo.com (M.A.); yasirali@awkum.edu.pk (Y.A.); zia_biotek@awkum.edu.pk (Z.-u.I.); 2 Department of Biotechnology, COMSATS University Islamabad, Abbottabad Campus, Abbottabad 22060, Pakistan; ali@cuiatd.edu.pk; 3 Attaur Rahman School of Applied Biosciences, NUST, Islamabad 44000, Pakistan; maria.arshad14@gmail.com; 4 Department of Botany, Abdul Wali Khan University Mardan, Khyber Pakhtunkhwa 23200, Pakistan; mamoona@awkum.edu.pk; 5 Department of Zoology, College of Science, King Saud University, Riyadh I1451, Saudi Arabia; smaodaa@ksu.edu.sa (S.N.M.); alfarraj@ksu.edu.sa (S.A.A.-F.); helserehy@ksu.edu.sa (H.A.E.-S.); 6 Marine Science Department, Faculty of Science, Suez Canal University, Ismailia 41522, Egypt

**Keywords:** SNPs, rheumatoid arthritis, miRNA, gene–gene interaction, therapeutic sites

## Abstract

**Simple Summary:**

Rheumatoid arthritis (RA) is a complex disease resulting from multiple genetic and environmental pathogenic factors. The genetic factors include single-nucleotide polymorphisms (SNPs), which have been reported to be associated with RA, but their specific role in the pathogenesis of RA remains unexplained. This study explains the potential role of RA risk-associated SNPs in its pathogenesis in order to provide a basis for understanding the genetic complexity of RA. Several roles of these SNPs are described in this study, and may also aid in the design of a therapeutic strategy for RA. Furthermore, novel potential therapeutic sites have also been researched, resulting in the identification of three novel therapeutic targets. The therapeutic strategies for the treatment of RA include inflammatory pathway-targeting drugs, which alleviate inflammation in joints. There is always a need for novel therapeutic targets that can play a role in alleviating inflammation in autoimmune diseases including RA. Therefore, these novel therapeutic sites are very important, and further experimental studies are required.

**Abstract:**

Single-nucleotide polymorphisms (SNPs) are reported to be associated with many diseases, including autoimmune diseases. In rheumatoid arthritis (RA), about 152 SNPs are reported to account for ~15% of its heritability. These SNPs may result in the alteration of gene expression and may also affect the stability of mRNA, resulting in diseased protein. Therefore, in order to predict the underlying mechanism of these SNPs and identify novel therapeutic sites for the treatment of RA, several bioinformatics tools were used. The damaging effect of 23 non-synonymous SNPs on proteins using different tools suggested four SNPs, including rs2476601 in *PTPN22*, rs5029941 and rs2230926 in *TNFAIP3*, and rs34536443 in *TYK2,* to be the most damaging. In total, 42 of 76 RA-associated intronic SNPs were predicted to create or abolish potential splice sites. Moreover, the analysis of 11 RA-associated UTR SNPs indicated that only one SNP, rs1128334, located in 3′UTR of *ETS1,* caused functional pattern changes in BRD-BOX. For the identification of novel therapeutics sites to treat RA, extensive gene–gene interaction network interactive pathways were established, with the identification of 13 potential target sites for the development of RA drugs, including three novel target genes. The anticipated effect of these findings on RA pathogenesis may be further validated in both in vivo and in vitro studies.

## 1. Introduction

Rheumatoid arthritis (RA) is a systemic autoimmune disease with approximately 1% prevalence worldwide, and its presence carries the risk of irreparable functional disability of inflamed joints due to articular damage [1]. Rheumatoid joints exhibit an inflammatory environment that favors the activation of T cells, B cells, macrophages, osteoclasts, and synovial fibroblasts [1]. These cells maintain crosstalk through the production of cytokines, which upon activation induce the secretion of enzymes and other products that contribute to the destruction of cartilage and bone tissues [2]. To date, the etiology of RA remains obscure. However, some authors suggest that the over-reactive immune system in RA is due to both genetic and environmental factors [3,4]. It has been estimated that the inheritability of RA is around 65%, which underlines the importance of its genetics [5,6,7]. Among the genetic factors, several genes have been associated with RA susceptibility [7,8,9,10,11]. Genetic association studies based on different populations have identified more than 100 genomic loci [10,11,12] which account for approximately 15% of the variance [12,13]. However, the actual underlying genetic mechanism concerning SNPs has not been determined.

SNPs are genetic variations that account for ~0.1% differences in populations. The coding region contains about 50% SNPs, with ~25% being missense and ~25% being silent or synonymous [14,15]. Non-coding SNPs may change mRNA stability and promoter activity by creating or disrupting the miRNA sites, causing an altered gene expression with the consequent up-or down-regulation of a gene. The role of these variants in relation to RA risk needs to be explored for the proper elucidation of the biological pathways involved. Besides understanding the underlying disease mechanism, SNP analysis will help in the development of new drugs against RA.

In this study, 152 RA-associated SNPs were characterized and their functional importance with regard to the respective genes and their products was examined in detail. In addition, we investigated the gene–gene interaction patterns and suggested 13 potential and highly significant target sites for the development of RA drugs.

## 2. Results

### 2.1. SNP Retrieval

**Figure 1:**
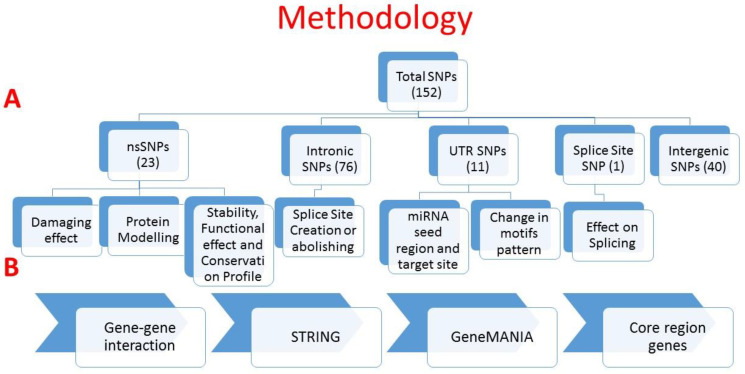
Workflow of the study. (**A**) SNP distribution and analysis with regard to their respective effects. (**B**) Prediction of gene–gene interactions and core region genes. This figure was generated using Microsoft PowerPoint 2016.

**Figure 2:**
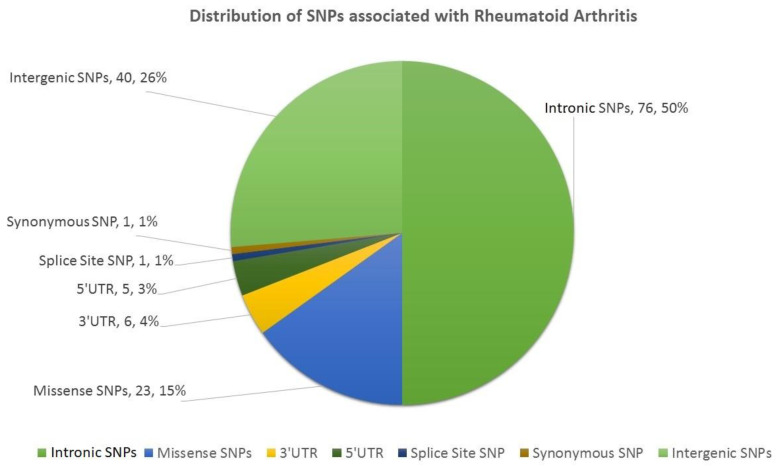
Distribution of SNPs associated with RA, represented in a pie chart. This figure was generated using Microsoft Excel 2016.

The first step in this study involved mining the literature from PubMed and Web of Science (Figure 1). We found 152 SNPs (located in 75 genes) in the literature which were reported to be associated with RA (Table S1). Of these SNPs, 76 SNPs were intronic (located in 51 genes), 40 SNPs were intergenic, 23 SNPs were missense (located in 18 genes), 11 SNPs were in the UTRs of 9 genes (6 SNPs in 3′UTR and 5 SNPs in 5′UTR), 1 SNP was synonymous, and 1 belonged to the splice site (Figure 2). Details on all the SNPs are provided in Table S1. The associations of these SNPs with the clinical characteristics of RA patients are provided in Table S2.

### 2.2. Characterization of nsSNPs

The 23 nsSNPs that were retrieved from the literature and were found to be potentially associated with RA were analyzed using different tools. These nsSNPs are listed in Table 1 along with amino acid residue change and global MAFs.

### 2.3. Prediction of Damaging Effects of nsSNPs

The damaging effects of nsSNPs on proteins were predicted using five different *in-silico* tools, which included PhD-SNP, SNPs&GO, PolyPhen2, PROVEAN, and SIFT. For PhD-SNP and SNPs&GO, a threshold value of 0.5 was set and any prediction beyond this value was considered deleterious. According to these tools, all the nsSNPs were found to exhibit a neutral effect. PolyPhen2 predicted the nsSNPs to be probably damaging, possibly damaging, and benign on a scale of 0–1, with 1 being the most damaging. According to PolyPhen2, 5 out of 23 nsSNPs were predicted to be probably damaging. In the case of PROVEAN, a threshold value of −2.5 was selected and any prediction below this value was considered deleterious. Out of the total 23 nsSNPs, PROVEAN predicted four SNPs to be deleterious. In SIFT, a tolerance index (TI) of 0.05 was selected and the predictions with values less than this were considered deleterious. SIFT predicted three of the total nsSNPs to be deleterious. Finally, four nsSNPs (corresponding to three genes) which were predicted to be damaging or deleterious by at least two of the five *in-silico* tools were selected for further analysis (Table 2). The selected nsSNPs were cross-checked for consistency using the Ensembl genome browser (release 96), MetalR, Mutation Assessor, REVEL, and CADD. The selected nsSNPs included *PTPN22* rs2476601, *TNFAIP3* rs5029941 and rs2230926, and *TYK2* rs34536443. Ensembl results for these four nsSNPs are listed in Table 3. These results were in accordance with our prediction results, which confirmed the reliability of our methodology. Results for all the nsSNPs are provided in Table S3.

### 2.4. Prediction of Stability, Functional, Structural Effects, and Conservation Profile of Proteins

I-Mutant was used to predict the effects of the nsSNPs on protein stability. This tool predicted that 21 of the 23 nsSNPs would decrease protein stability, while two nsSNPs (*rs2233433* and *rs5029941*) showed the opposite results. For the structure-based predictions, we used CUPSAT (released January 2018) (http://cupsat.tu-bs.de/, accessed on 2 February 2021) to cross-check the reliability of these predictions. The CUPSAT predicted eight nsSNPs (34.78%) to be stabilizing as compared to I-Mutant (8.70%), while 15 nsSNPs (65.22%) were predicted to be destabilizing as compared to I-Mutant (91.30%). This tool also predicted changes in energy upon amino acid substitution (Table 4). The MutPred server was used to predict different structural and functional effects, such as the creation of glycosylation and catalytic sites, altered membrane proteins, the gain of intrinsic disorder, the loss of allosteric sites, etc. Only one *(rs2230926)* of the 23 nsSNPs caused gain of an intrinsic disorder and loss of an allosteric site, while all the remaining nsSNPs were predicted to have no structural or functional effects on proteins. The ConSurf tool was used to predict the evolutionary conservation profile of all the amino acids of a protein. The protein FASTA sequences of each protein were submitted to ConSurf, which generated the conservation profiles of each proteins (Figure S1). Interestingly, only 2 of the 23 nsSNPs were located at buried amino acid sites and three were located at highly conserved and functional residues, while all the remaining nsSNPs were present at the exposed residues. The findings regarding the stability, functional and structural effects, and conservation profile of proteins are listed in Table 5. 

### 2.5. Modeling of Proteins

**Figure 3:**
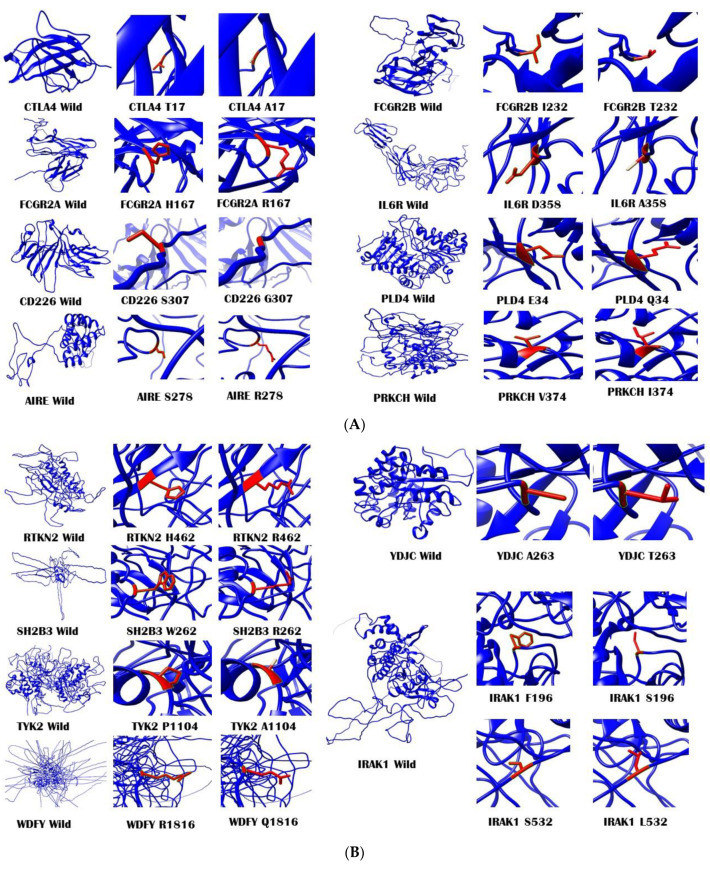

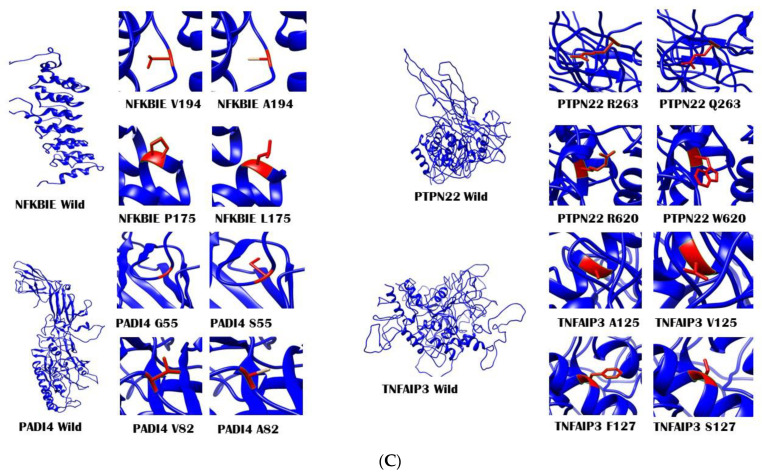
Modeled structures using MODELER v9.22 for wild-type proteins along with a close-up of wild and mutated amino acid residues. (**A**) Modeled structures for CTLA4, FCGR2A, CD226, AIRE, FCGR2B, IL6R, PLD4, and PRKCH. (**B**) Modeled structures for RTKN2, YDJC, SH2B3, TYK2, WDFY4, and IRAK1. (**C**) Modeled structures for NFKBIE, PADI4, PTPN22, and TNFAIP3. All the protein structures were visualized, and figures were generated using Chimera v1.11 software (https://www.cgl.ucsf.edu/chimera/, accessed on 3 February 2021). The structures were then assembled and combined using Microsoft PowerPoint 2016.

The protein modeling was performed using comparative homology modeling with MODELLER v9.22. For each of the proteins, NCBI BLAST was utilized and the source database was set as the Protein Data Bank (pdb). The best-matching templates for each of the proteins were selected for homology modeling. The templates, along with the percentages of identity and coverage, are listed in Table 6. For each protein, the templates were searched and their respective pdb files were downloaded from the RCSB Protein Data Bank. Python script files were written according to the protocol by Andrej Sali Laboratory (https://salilab.org/modeller/tutorial/, accessed on 3 February 2021). For each homology model, the best models with the lowest DOPE value and highest GA341 score were selected for final modeling. The final models were viewed and studied using Chimera v1.11 (https://www.cgl.ucsf.edu/chimera/, accessed on 3 February 2021) [13]. Mutant structures were modeled using Chimera v1.11 by mutating the residue of interest. All the modeled structures along with mutated residues are given in Figure 3A–C. The RMSD values for each of the mutant proteins were calculated using TM-align for every nsSNP. Interestingly, the RMSD values for all the mutated structures were zero. To validate our designed structures, Ramachandran plot assessment was used. The RAMPAGE values for each modeled structure are listed in Table 7. All the modeled structures had outlier region residues <10%.

### 2.6. Characterization of Intronic SNPs

The SNPs located in the intronic regions of different genes, which were reported to be associated with RA, were compiled and subjected to characterization using ESEfinder3.0. The DNA FASTA sequences for each of the SNPs were retrieved from dbSNP database and are provided in Table S3. All the FASTA sequences, for both the wild-type and mutated proteins, were submitted, and the exon splicing enhancer sites were predicted in both the sequences separately. Of all the 76 intronic SNPs, 42 SNPs were predicted to change the functional pattern and were noted accordingly (Table 8). Of the 42 SNPs, 22 SNPs (located in 27 genes) were found to destroy potential splice sites, 16 SNPs created new splice sites, and 4 SNPs created 1 and destroyed other potential splice sites.

### 2.7. Characterization of Splice Site SNPs

The splice site SNP rs2004640, located in the *IRF5* gene, was characterized to investigate its potential functional effect on splicing using NetGene2 (http://www.cbs.dtu.dk/services/NetGene2/, accessed on 20 February 2021), the Alternative Splice Site Predictor (ASSP) (http://www.wangcomputing.com/assp/, accessed on 20 February 2021), ESEfinder release 3.0 (http://krainer01.cshl.edu/cgi-bin/tools/ESE3/esefinder.cgi, accessed on 20 February 2021), and Human Splicing Finder v3.1 (HSF v3.1) (http://www.umd.be/HSF3/, accessed on 20 February 2021). NetGene2 and ASSP did not predict any functional effect of this SNP on the splicing mechanism. However, ESEfinder3.0 predicted one potential splice site to be broken at 4 bp upstream of the SNP position, where the human SRSF2 protein may react. The HSF3.1 used the HSF matrices and MaxEnt algorithms to predict the creation of a new donor splice site. HSF3.1 predicted 1 potential splice site to be created, 1 enhancer SF2 motif to be broken, 3 silencer motifs (method of Sironi et al.) to be broken, 1 silencer motif to be created, and 1 silencer IIEs motif to be broken. The details of the results of ESEfinder3.0 and HSF3.1 are listed in Table 9.

### 2.8. Characterization of UTR SNPs

The SNPs in the UTR region were studied using UTRScan, PolymiRTS Database, and MicroSNiPer. The DNA FASTA sequences for both the 3′UTR and 5′UTR were submitted to UTRScan, which analyzed the sequences without mutations. For this reason, the FASTA sequences for both the wild-type and mutated sequences were submitted separately (provided in Text S1), and the changes in functional patterns due to the UTR SNPs were noted. Of the 11 UTR SNPs (6 in 3′UTR and 5 in 5′UTR), only 1 SNP in the 3′UTR (*rs1128334* located in *ETS1* gene) was found to cause significant pattern changes, resulting in the creation of BRD-BOX. All the other SNPs did not indicate any significant changes in the expression pattern of the respective genes. The 3′UTR SNPs were further submitted to PolymiRTS database to investigate if they could disrupt or create miRNA binding site. Of the 6 SNPs in 3′UTR, 3 SNPs were predicted to create 3 miRNA binding sites and disrupt 6 miRNA binding sites. The potential effect of the UTR SNPs on destroying the possible miRNA seed region in 3′UTR was investigated using MicroSNiPer. The results showed that there were 5 SNPs in the 3′UTR which could possibly destroy 10 miRNA seed regions. The results of UTRScan, PolymiRTS database, and MicroSNiPer are listed in Table 10.

### 2.9. Gene–Gene Interactions of RA Associated Genes

**Figure 4:**
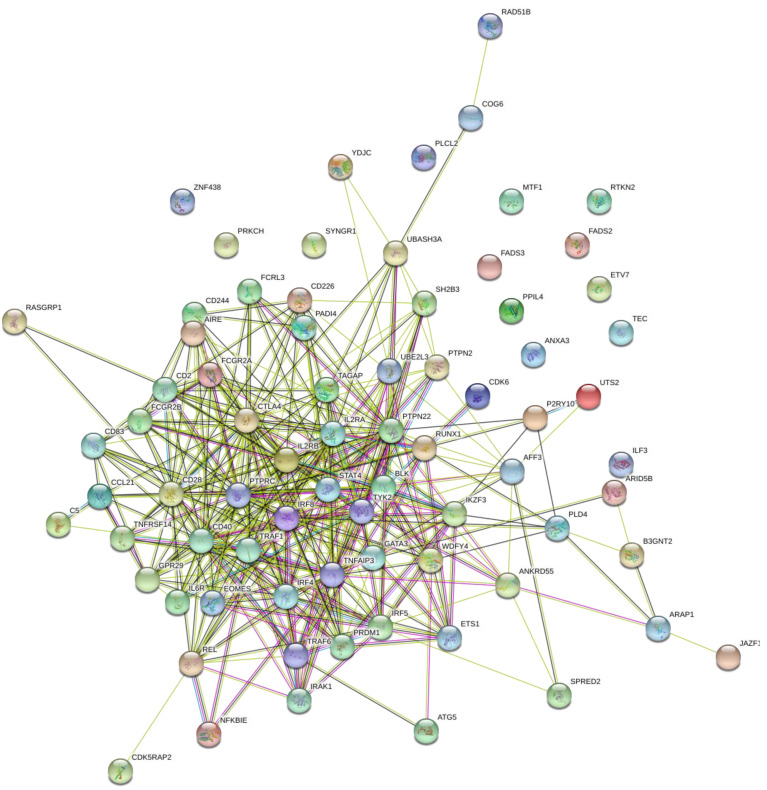
Gene–gene interaction model of 75 RA-associated genes using STRING. This figure was downloaded as a high-quality image file from STRING v11.0 (https://string-db.org/, accessed on 25 February 2021). A guide, representing the different colors in this figure, is provided in Supplementary Figure S2.

**Figure 5:**
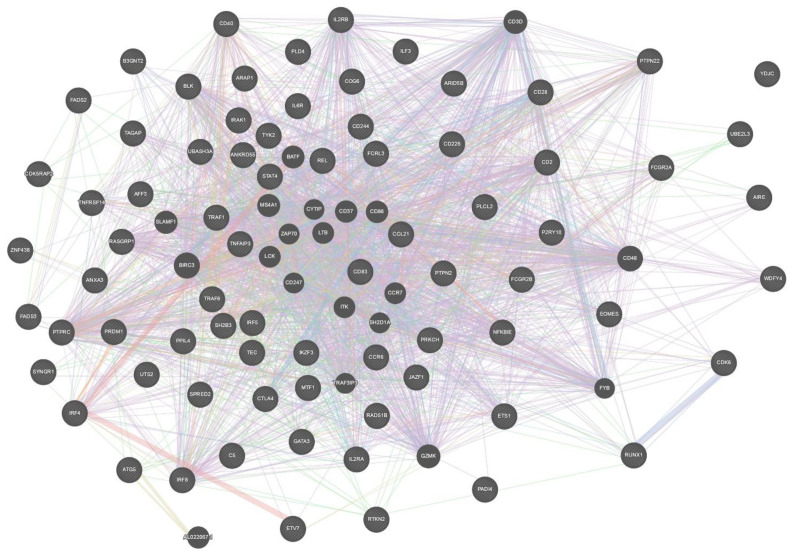
Gene–gene interaction network generated by GeneMANIA for all the interaction types. This figure was downloaded as a high-quality image file from GeneMANIA v3.5.1 (https://genemania.org/, accessed on 25 February 2021).

**Figure 6:**
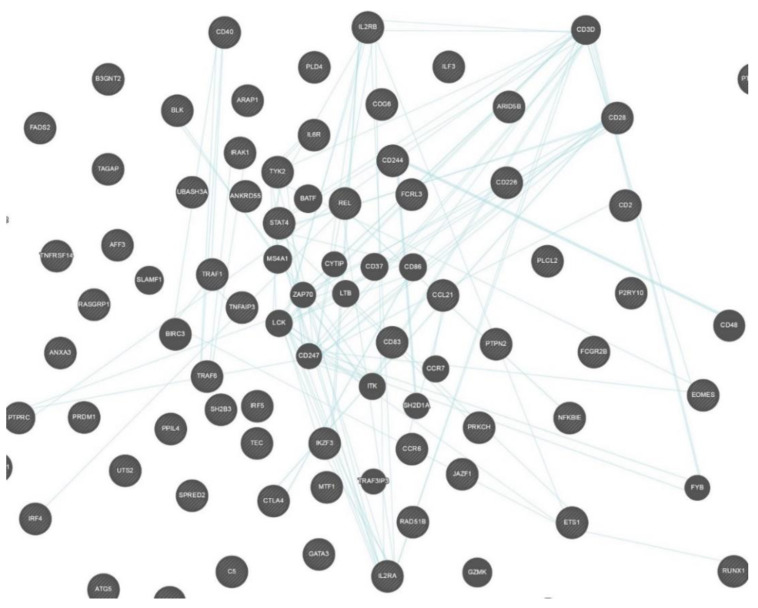
Gene–gene interaction network predicted by GeneMANIA showing only co-localized genes in pathways. This figure was downloaded as a high-quality image file from GeneMANIA v3.5.1 (https://genemania.org/, accessed on 25 February 2021).

STRING and GeneMANIA were used to analyze the gene–gene interactions of all the 75 genes with potential RA-associated SNPs. Both the tools were fed with gene symbols and the outcomes were recorded. The cutoff value used for both the tools was 0.1. The score ranged on a scale of 0 to 1, with 1 being the best. STRING predicted a total of 365 interactions between 60 genes (shown in Figure 4) and the details are provided in Table S4. The remaining 15 genes were not predicted to have any interaction with any of the investigated genes. A total of 18 genes were predicted to be core region genes, including *IL2RB*, *STAT4*, *CTLA4*, *PTPN22*, *TYK2*, *BLK*, *GATA3*, *TNFAIP3*, *IRF4*, *EOMES*, *IL6R*, *IRAK1*, *TRAF6*, *CD28*, *CD40*, *TRAF1*, *PTPRC*, and *IL2RA.* The GeneMANIA predictions included co-expression (71.46%), co-localization (12.75%), physical interactions (7.83%), predicted theoretical interactions (3.21%), shared protein domains (1.82%), pathways (1.66%), and genetic interactions (1.27%) (Figure 5). Additional genes which were predicted to have role in the pathogenesis of RA were *IL2RB*, *CD3D*, *CD28*, *CD2*, *CD226*, *CD244*, *CCL21*, *CCR6*, *REL*, *BLK*, *IRAK1*, *TYK2*, *STAT4*, *TRAF1*, *TRAF6*, *PTPRC*, *CD247*, *TNFAIP3*, *CD83*, *ITK*, *IL6R*, *EOMES*, *ETS1*, and *IL2RA* (Figure 6).

## 3. Discussion

SNPs hold a significant role in the pathogenesis of a disease. *In-silico* characterization underlines the possible functional significance of SNPs in both the coding and non-coding regions of a gene, for example through alteration of mRNA stability, promoter activity, and miRNA sites [14,15,16]. However, the actual underlying genetic mechanism concerning the SNPs has not been determined. This is similarly the case in RA-associated SNPs. Previously, some studies were carried out to identify the association of SNPs with RA [14,15,16,17], but *in-silico* characterizations of these SNPs are scarce.

In our study, we characterized 152 RA-associated SNPs. Of these SNPs, 76 (50%) were intronic, 40 (26.31%) were intergenic, 23 (15.13%) were missense, 6 (3.94%) were in the 3′UTR, 5 (3.28%) were in the 5′UTR, and 1 (0.67%) each belonged to the splice site and coding region, respectively. From these observations, we concluded that RA has a significant association with intronic SNPs (50%), although a large number (26.31%) of SNPs were also observed in intergenic regions. Besides, the role of SNPs in regulation of genes may be less significant (7.24%, 3′ + 5′UTRs SNPs). 

nsSNPs are sometimes damaging and have a significant impact on disease pathogenesis [18]. They also contribute to altered drug responses when occurring in the active site of the drug’s target [19]. In our study, PhD-SNP and SNPs&GO predicted none of the nsSNPs to be damaging, while PolyPhen2, PROVEAN, and SIFT predicted 5 (21.74%), 4 (17.39%), and 3 (13.04%) of the nsSNPs, respectively, to be damaging (Table S3). The nsSNP *TYK2 rs34536443* had the most damaging effect, with a SIFT score of 0.007, a PROVEAN score of −6.755, and a PolyPhen2 score of 1.00. The nsSNPs which were predicted to be damaging by at least two of the five tools were cross-checked for consistency using the Ensembl genome browser, MetalR, Mutation Assessor, REVEL, and CADD. The selected nsSNPs included *PTPN22* rs2476601, *TNFAIP3* rs5029941 and rs2230926, and *TYK2* rs34536443. Notably, among these SNPs, *TYK2* rs34536443 was predicted to be the most damaging, with a CADD score of 26, a REVEL score of 0.586 (0.5 threshold), a MetalR score of 0.336 (0.5 threshold), and a Mutation Assessor score of 0.36 (0.5 threshold). CADD scores of 10, 20, and 30 corresponded to 10%, 1%, and 0.1% of the most damaging SNPs, respectively. This result shows that out of 23 nsSNPs, the *TYK2* rs34536443 might be the most significant in terms of functional impairment. According to I-Mutant predictions, all the nsSNPs had a deteriorating effect on protein stability, except for two SNPs (rs2233433 and rs5029941). It should be noted that the predictions of I-Mutant were based on the sequence of proteins and not on their structure. Among the predicted destabilizing nsSNPs, *PTPN22* rs2476601 had the highest change in energy at −6.98 kcal/mol, followed by *PADI4* rs11203366 and *TNFAIP3* rs2230926, with energy change values of −5.91 kcal/mol and −4.58 kcal/mol, respectively. *TYK2* rs34536443, which was found to have a significant damaging effect on protein function, was not only predicted as stabilizing but also had very high energy change of 6.73 kcal/mol. MutPred predicted only one nsSNP (*TNFAIP3* rs2230926) to have functional effects. Changes were shown in eukaryotic linear motif (ELM) sites, with in a gain in intrinsic disorder and the loss of the allosteric site at position R123 in the TNFAIP3 protein. The ELM motif is a resource database which is dedicated to short linear motifs (SLiMs) [15]. These are small proteins, considered as functional modules, which play an important role in the modifications of protein sequences and protein–protein interactions (PPI) [16,17]. Many important cellular processes, such as cell signaling, protein stability, trafficking, molecular mechanism switching, and cell cycle progression are mediated by SLiMs [17,18,19,20]. Six different ELM motifs were predicted to be affected by *TNFAIP3* rs2230926, including ELME000053 (GSK3 phosphorylation site), ELME000064 (CK2 phosphorylation site), ELME000106 (cyclin docking motif), ELME000146 (PCSK cleavage site), ELME000220 (FHA phosphopeptide ligands), and ELME000239 (USP7 binding motif). None of the other nsSNPs was predicted to cause any functional effect on the ELM motifs or gain or loss of any other site. ConSurf was then used to generate the conservation profiles of all the proteins, where nsSNPs were located (Figure S1). ConSurf uses solvent accessibility along with evolutionary conservation data to predict the functional and structural effect of nsSNPs that may cause human health problems [21]. Only two nsSNPs, *FCGR2B* rs1050501 and *TNFAIP3* rs5029941, were predicted to be located at buried residues, while three nsSNPs (*PTPN22* rs33996649 and rs2476601 and *TYK2* rs34536443) were predicted to be located at highly exposed structural and functional residues. All the other nsSNPs were predicted to be exposed. This explains that most of the RA-associated nsSNPs (91.30%) are located at exposed residues. In order to model the protein structures and study amino acid residue changes resulting from these nsSNPs, the comparative homology modeling tool MODELER v9.22 was used. MODELLER is the most widely used tool for the comparative homology modeling of proteins. We first searched for the highest identical templates with the maximum coverage percentages. For all the proteins, the templates had >30% identity, except for PLD4, where the templates 2ZE4, 4GGJ, 2ZE9, and 1BYR had identity values of 26.52%, 26.32%, 25.97%, and 24.70%, respectively. For all the proteins, many templates were available as a result of the NCBI BLAST search. Of these, only four templates with the highest identity and maximum coverage were selected, except for YDJC, for which only one template (2I5I with 37.23%) was selected. MODELLER v9.22 predicted five structures for each of the proteins, with slight differences. The structure with the lowest discrete optimized protein energy (DOPE) value and highest GA341 score was selected. DOPE calculates the energy of proteins. It is based upon the non-interacting atoms of the modeled protein, with the radius dependent on the template [22]. GA341 scores are calculated on the basis of template and modeled structure identity percentages. Scores range from 0 to 1, where 1 shows the best-modeled structure [23]. The structures modeled with MODELLER v9.22 were then viewed and studied in Chimera v1.11, and mutant protein structures were developed. Root-mean-square deviation (RMSD) values were calculated for each of the mutant structures. RMSD values are used for the calculation of differences between the alpha-carbon backbone of wild-type and mutant protein structures [24,25]. For all the wild-type proteins, the RMSD values were 0.00, meaning that these nsSNPs may not have significant effect on any alteration in the protein’s carbon backbone. For the validation of the protein structures, Ramachandran plot analysis was carried out using RAMPAGE, which predicted all the structures to be valid. For the PLD4 protein, the templates had <30% similarities, but its modeled structure had only 4.3% outlier residues, which validated its structure. 

*In-silico* characterization of the nsSNPs suggested that the SNPs rs33996649 and rs2476601 (*PTPN22)*, rs5029941 and rs2230926 (*TNFAIP3),* and rs34536443 (*TYK2)* have prominent functional effects on the protein structure and function. We cross-checked these five nsSNPs with the literature and found evidence for only two nsSNPs (*TNFAIP3* rs2230926 and *TYK2* rs34536443). A recent study showed that *TNFAIP3* rs2230926 decreased the activity of *NF-κB* by two-fold [26], but the exact molecular mechanism of how this decrease happened was unknown. For *TYK2* rs34536443, both *in-silico* and in vivo studies have shown a decrease in the enzymatic activity of tyrosine kinase 2 (*TYK2*) due to this SNP [27,28,29]. For the two SNPs (*TNFAIP3* rs2230926 and *TYK2* rs34536443), our predictions are in agreement with the previous studies [26,27,28,29], while there was no literature-based evidence for the other three nsSNPs *(PTPN22* rs33996649 and rs2476601 and *TNFAIP3* rs5029941). 

The only known functional effect of the SNPs located in the intronic regions on the gene or its product is the effect of these intronic SNPs on the splicing phenomenon. Intronic SNPs may create or abolish the interaction sites for human SR proteins. The creation of a new site for a human SR protein with higher value may result in alternative splicing, which causes the alteration of the protein. Human SR protein family members, including SRSF1, SRSF2, SRSF5, and SRSF6, have been found to have an important role in splicing mechanisms [30,31,32,33]. The binding of the U1 snRNP to the 5′ splice site and the binding of U2 snRNP to the 3′ splice site are promoted by these SR proteins, as well as the events in pre-spliceosome, and mature spliceosome are also linked with these SR proteins [34,35,36,37]. Of the 76 investigated intronic SNPs, 42 (55.26%) SNPs were predicted to cause functional changes in human SR protein binding. A total of 26 SNPs (34.21%) were predicted to demolish the SR protein binding site, while 20 SNPs (26.31%) were predicted to create a new SR protein binding site. Our results predicted 55.26% of the RA-associated intronic SNPs to cause changes in the SR protein binding sites, which may explain their functional significance in the pathogenesis of RA. However, upon a literature survey, we did not find any evidence of the characterization of any of these SNPs *in-silico*, in vivo, or in vitro. In vivo and in vitro studies are needed for the characterization of the functional importance of these SNPs and the association mechanism with RA and other autoimmune diseases. 

The splice site SNP rs2004640 (*IRF5)* was investigated using different tools for its potential effect on the splicing mechanism. As it is located at the splice site, it has a prime importance in splicing of *IRF5*. Many studies have demonstrated that rs2004640 increases *IRF5* mRNA to a level ~2-fold higher than the wild-type allele [38,39]. Clark and co-workers showed that this polymorphism decreased 1C and 1D exon usage but did not alter mRNA stability [38]. Hence, it is evident that this splice site SNP may cause alternative splicing of *IRF5* mRNA and may lead to increased IRF5 production. Our predicted results are also in accordance with the literature. The creation of a potential splice site 4 bp upstream of rs2004640 was predicted by both ESEfinder3.0 and HSF3.1, with high values of 85.64 (threshold 60) and 2.9 (threshold 1.867), respectively. Both the tools predicted the same enhancer motif SRSF2 (IgM-BRCA1) to be broken, with scores of 78.92 and 2.95 by HSF3.1 and ESEfinder3.0, respectively. Therefore, our findings support the previous studies and provide an insight into the mechanism of splicing alteration, which may be caused as a result of this SNP. 

Different hereditary diseases such as immune deficiency syndromes and cancer have been linked to mutations in the UTRs of the genes, which have been reported to have vital roles in the stability, translational efficiency, and localization of mRNA [40]. Both the 5′UTR and 3′UTR have key functions in mRNA stability and its expression. The processing and translation of mature mRNA can be severely affected by mutations in the UTR regions of the genes, which can lead to the changes in gene expression patterns [41]. The SNPs located in the 5′UTR are associated with the changes in mRNA stability, binding capacity to ribosomes, and translational regulation, affecting the RNA half-life. The localization, translational efficiency, polyadenylation, stability, and binding specificity of miRNA (microRNA) may be altered by SNPs in the 3′UTR, which effects the expression patterns of genes [15]. In our study, we analyzed 11 UTR SNPs, including six 3′UTR SNPs and five 5′UTR SNPs using UTRScan, the PolymiRTS database, and MicroSNiPer. UTRScan predicted only one SNP, rs1128334, located in 3′UTR of *ETS1,* to cause functional pattern changes in BRD-BOX. BRD-BOX is a seven-nucleotide motif, which, upon presence in the 3′UTR, controls the activation of gene. When it is lost from the UTR, it results in the hyper-activation of gene [42]. Our study suggested that the creation of BRD-BOX by rs1128334 (*ETS1)* would be protective against disease. Similarly, it has been shown that the presence of this SNP reduces susceptibility to autoimmune diseases [43,44]. The PolymiRTS database predicted that three 3′UTR SNPs (rs3811021 *(PTPN22)*, rs2070197 (*IRF5),* and rs1128334 (*ETS1)*) might affect miRNAs by creating or abolishing the miRNA target sites, therefore contributing to the up- and down-regulation of genes. Similarly, MicroSNiPer predicted five 3′UTR SNPs (Table 8) which affected the miRNA target sites by changing the seed length. However, none of the 5′UTR SNPs was found to have potential role in the pathogenesis of RA, while the five 3′UTR SNPs have been proven to change the functional expression pattern of genes by various means, including destroying or creating miRNA binding sites and creating BRD-BOX. 

Gene–gene interaction is an important phenomenon with a key role in the pathogenesis of multi-gene hereditary diseases. There are many genes that have been reported to have significant associations with RA, with known and unknown pathogenesis patterns. We used STRING and GeneMANIA for the prediction of different gene–gene interaction mechanisms. From STRING predictions, we found that 18 of the total 76 genes were in the core region (Figure 4), while GeneMANIA predicted type-specific interactions in which 24 genes were found to be interactive in pathways. Thirteen genes were common to both core-region genes and interactive pathway genes, including *IL2RB, STAT4, TYK2, BLK, TNFAIP3, EOMES, IL6R, IRAK1, TRAF6, CD28, TRAF1, PTPRC,* and *IL2RA.* These genes are important in the pathogenesis of RA and may be considered as potential targets for drug development. Previously, 10 genes out of these 13 were suggested for drug targeting in RA patients, as reviewed by Yamamoto and co-workers in 2015 [2]. Three genes (*BLK, EOMES,* and *TRAF1)* are predicted to be novel as potential drug targets. 

The *BLK* gene encodes a nonreceptor tyrosine-kinase of the src family of proto-oncogenes that are typically involved in cell proliferation and differentiation. The protein has a role in B-cell receptor signaling and B-cell development. The *BLK* risk haplotype was found to be associated with enhanced activation of BCR-stimulated B cells with an increase in T cell–B cell collaboration, at least in part due to differential up-regulation of CD86, and with attendant effects on the isotype distribution of the switched memory B cell repertoire. This is likely to reflect a common mechanism for *BLK*-mediated genetic risk in autoimmune diseases associated with *BLK* [45]. The *EOMES* gene belongs to the TBR1 (T-box brain protein 1) sub-family of T-box genes that share the common DNA-binding T-box domain. The encoded protein is a transcription factor which is crucial for embryonic development of the mesoderm and the central nervous system in vertebrates. The protein may also be necessary for the differentiation of effector CD8+ T cells which are involved in defense against viral infections. The protein expression of EOMES was increased in T cells from healthy donors homozygous for the *PTPN22* risk allele and correlated with a decreased number of naïve CD4^+^ T cells [46]. An accumulation of EOMES^+^CD4^+^ T cells was also observed in the synovial fluid of RA patients with a more pronounced production of perforin-1 in *PTPN22* risk allele carriers. The protein encoded by this *TRAF1* is a member of the TNF receptor (TNFR)-associated factor (TRAF) protein family. TRAF proteins associate with and mediate the signal transduction from various receptors of the TNFR superfamily. Genome-wide association studies have identified an association between SNPs in the 5′ untranslated region of the TRAF1 gene, with increased incidence and severity of rheumatoid arthritis and other rheumatic diseases. The loss of TRAF1 from chronically stimulated CD8 T cells results in desensitization of the 4-1BB signaling pathway, thereby contributing to T cell exhaustion during chronic infection. These apparently opposing roles of TRAF1 as both a positive and negative regulator of immune signaling have led to some confusion in the literature. Thus, through gene–gene interactions, we suggested 13 potential drug target sites, of which 3 were novel target genes. 

All of our statements are based on evidence from the literature combined with predictive results of the *in-silico* tools, which used different algorithms and statistical formulas for their predictions. Our study provides a detailed insight into the mechanism of effects of different SNPs belonging to different SNP categories and associated with RA. In order to further validate the effects of these SNPs as predicted by our study, in vitro and in vivo studies are needed. Model organisms with wild-type and mutated alleles, separately, are needed to be studied for further understanding.

## 4. Methodology

A workflow for the complete methodology is given in Figure 1.

### 4.1. SNP Retrieval

RA-associated SNPs were searched in PubMed and Web of Science. The data were mined from original research articles published in indexed journals. Retrieved SNPs were divided into four categories; intronic SNPs, non-synonymous SNPs, UTR SNPs, and intergenic SNPs. Information related to the mined SNPs, such as their global minor allele frequencies (MAF), nucleotide change, amino acid residual change for nsSNPs, FASTA sequences, etc., were retrieved for each SNP from NCBI dbSNP (accessed on 2 January 2021). 

### 4.2. Characterization of nsSNPs

The RA-associated nsSNPs were characterized as below:

#### 4.2.1. Most Damaging Prediction

Five different *in-silico* tools were used to predict the effect of nsSNPs on respective proteins. These tools included: Protein Variation Effect Analyzer (PROVEAN) (http://provean.jcvi.org/seq_submit.php, accessed on 5 January 2021) [47], Sorting Intolerant from Tolerant (SIFT) (https://sift.bii.a-star.edu.sg/www/SIFT_seq_submit2.html, accessed on 5 January 2021) [48,49], SNPs&GO (http://snps.biofold.org/snps-and-go/snps-and-go.html, accessed on 5 January 2021) [50], Predictor of Human Deleterious SNP (PhD-SNP) (http://snps.biofold.org/phd-snp/phd-snp.html, accessed on 5 January 2021) [51], and Polymorphism Phenotyping 2.0 (PolyPhen 2.0) (http://genetics.bwh.harvard.edu/pph2/, accessed on 5 January 2021) [52]. The FASTA sequences of the respective proteins along with their amino acid residue changes were submitted for each nsSNP.

#### 4.2.2. Protein Stability, Structural and Functional Effects, and Conservation Profile Prediction

To predict the effect of nsSNPs on the stability of protein, I-Mutant 2.0 (http://folding.biofold.org/i-mutant/i-mutant2.0.html, accessed on 8 January 2021) was used [53]. The predictions were made for the stability of mutated protein along with the reliability index (RI) on a scale of 0–10, where 0 and showed the minimum and maximum reliability index, respectively. The structural and functional effects of nsSNPs on protein were predicted using MutPred 1.2 (http://mutpred.mutdb.org/, accessed on 9 January 2021) [54]. This predicted the effect of substituted amino acids on proteins, with *p* values of <0.05 and <0.01 being considered as confident and very confident, respectively. The conservation profile of each protein was predicted with the help of the ConSurf tool (http://consurf.tau.ac.il/2016/, accessed on 15 January 2021) [55] using 50 different homologous sequences. 

### 4.3. Protein Modeling

Protein modeling was done using MODELLER v9.22 [56]. The templates for each protein to be modeled were searched using NCBI BLAST, and those with higher percentage identity and coverage were finally chosen. These templates were later downloaded from the RCSB Protein Data Bank (http://www.rcsb.org/, accessed on 2 February 2021). Command files for each of the protein modeling were prepared separately. For the mutants, the protein structures were modeled using the in-built feature in Chimera v1.11 and the amino acid residual changes were made manually and individually in their respective protein structures. After protein modeling, TM-align (https://zhanglab.ccmb.med.umich.edu/TM-align/, accessed on 5 February 2021) was used to calculate the root-mean-square deviation (RMSD) values for each mutant and wild-type protein. The RMSD values are associated with the functional effect of nsSNP on protein, therefore showing their role in pathogenesis. The higher the RMSD values, the greater the effect of nsSNPs on protein. Later on, the protein structures were validated using Ramachandran plot assessment (http://mordred.bioc.cam.ac.uk/~rapper/rampage.php, accessed on 14 February 2021). This plot showed the percentages of favored and allowed residues, as well as outlier regions, where the structures with residues less than 10% in the outlier regions were considered as good structures.

### 4.4. Characterization of Intronic SNPs

The effect of intronic SNPs could be detected either by demolishing or creating the splice site in respective genes. For this purpose, we used ESEfinder 3.0 (http://krainer01.cshl.edu/cgi-bin/tools/ESE3/esefinder.cgi, accessed on 16 February 2021) [57]. The FASTA sequences of all the intronic SNPs were retrieved from the dbSNP database. The FASTA sequences for wild-type and mutated sequences were submitted to ESEfinder 3.0, separately, for all the SNPs. ESEfinder3.0 predicted the potential exonic splicing enhancer (ESE) sites that could react with any of the 4 human SR proteins, which are SF1, SF2, SF5, and SF6.

### 4.5. Characterization of Splice Site SNP

Only 1 splice site SNP rs2004640, located in the *IRF5* gene, was reported to be associated with rheumatoid arthritis in Norwegian patients [58]. The characterization of this SNP was performed to investigate its possible effect in splicing. Four different tools were used for this purpose, which included NetGene2 (http://www.cbs.dtu.dk/services/NetGene2/, accessed on 20 February 2021) [59], the Alternative Splice Site Predictor (ASSP) (http://www.wangcomputing.com/assp/, accessed on 20 February 2021) [60], ESEfinder release 3.0 (http://krainer01.cshl.edu/cgi-bin/tools/ESE3/esefinder.cgi, accessed on 20 February 2021) [57], and Human Splicing Finder v3.1 (HSF v3.1) (http://www.umd.be/HSF3/, accessed on 20 February 2021) [61]. Default conditions were used in all the tools and DNA FASTA sequences were submitted to all the tools. Among these tools, HSF3.1 is the most advanced, as it not only predicts potential splice sites but also the exon splicing and silencing enhancers.

### 4.6. Characterization of UTR SNPs

SNPs in UTR regions have many important effects on mRNA stability and expression. To characterize 3′UTR and 5′UTR SNPs, UTRScan (http://itbtools.ba.itb.cnr.it/utrscan, accessed on 23 February 2021), the PolymiRTS Database 3.0 (http://compbio.uthsc.edu/miRSNP/, accessed on 23 February 2021) and MicroSNiPer (http://vm24141.virt.gwdg.de/services/microsniper/, accessed on 23 February 2021) were used. UTRScan used nucleotide sequences to identify the pattern motif in UTR regions. The DNA FASTA sequences with and without SNPs were submitted individually and the changes in the functional pattern were noted. The PolymiRTS database and MicroSNiPer identified the effect of 3′UTR SNPs on the miRNA seed region and the target site. A list of the SNPs IDs was submitted to both the tools and their effects were recorded. 

### 4.7. Gene–Gene Interaction of RA Associated Genes

The interaction of all the genes selected for this study was investigated using STRING (https://string-db.org/, accessed on 25 February 2021) and GeneMANIA (https://genemania.org/, accessed on 25 February 2021) [62,63]. STRING predictions were based on co-expression, co-occurrence, gene fusion, biochemical and experimental data, while predictions by GeneMANIA were based on co-expression, similarity of protein domains, co-localization, and pathways. A gene list containing the official symbols of all the genes was uploaded and the results were analyzed to find the core region genes. 

## 5. Conclusions

From our study, it was concluded that RA risk-associated SNPs play an important role in the pathogenesis of RA. They contribute to about 15% of RA heredity. Different types of SNPs have their own respective roles in RA. Missense SNPs are found to cause deleterious effect on proteins, thus leading to diseased protein. We analyzed all the RA-associated nsSNPs, and their role in RA pathogenesis was evaluated using several *in-silico* tools. Of the 23 nsSNPs, 4 nsSNPs (*PTPN22* rs2476601, *TNFAIP3* rs5029941 and rs2230926, and *TYK2* rs34536443) were found to be deleterious, 21 nsSNPs were reported to decrease protein stability, 1 nsSNP (*TNFAIP3* rs2230926) was reported to have functional importance (affecting ELM motifs and causing a loss of allosteric sites and a gain of intrinsic disorder), and 3 nsSNPs (*PTPN22* rs33996649 and rs2476601 and *TYK2* rs34536443) were reported to be located at highly conserved, functionally important, and exposed residues. Intronic SNPs represented 50% of the SNPs that are associated with RA, and our results showed that 42 of 76 intronic SNPs resulted in the alteration of human SR protein binding sites, which may contribute to the splicing mechanism. One splice site SNP (*IRF5* rs2004640) was analyzed and found to be splice site donor. Five UTR SNPs (*PTPN22* rs3811021, *TAGAP* rs4709267, *IRF5* rs2070197 and rs10954213, and *ETS1* rs1128334) were found to alter miRNA binding site. One SNP *ETS1* rs1128334 was found to create BRD-BOX, which may down regulate gene expression. Besides SNP characterization, we also predicted gene–gene interactions to predict RA pathogenesis and identified core region genes that may act as potential targets for the development of RA drugs. Importantly, we found 13 core region genes, including *IL2RB*, *STAT4*, *TYK2*, *BLK*, *TNFAIP3*, *EOMES*, *IL6R*, *IRAK1*, *TRAF6*, *CD28*, *TRAF1*, *PTPRC*, and *IL2RA.* These core region genes could be used as potential therapeutic sites for the treatment of RA. Although our study was in detail and provided a comprehensive analysis of all the SNPs, experimental studies are needed for validation. Mouse models carrying any of these SNPs would be ideal for such experiments.

## Figures and Tables

**Table 1:** nsSNPs along with amino acid change and global MAFs associated with RA.

Gene	SNP ID	Amino Acid Change	Global MAF *
*PTPN22*	rs33996649	R263Q	T = 0.0110
	rs2476601	R620W	A = 0.0274
*PADI4*	rs11203366	G55S	G = 0.4754
	rs11203367	V82A	T = 0.4667
*CTLA4*	rs231775	T17A	G = 0.4273
*TNFAIP3*	rs5029941	A125V	T = 0.0060
	rs2230926	F127S	G = 0.1396
*FCGR2A*	rs1801274	H167R	G = 0.4417
*FCGR2B*	rs1050501	I232T	C = 0.1859
*IRAK1*	rs1059703	S532L	G = 0.4832
	rs1059702	F196S	A = 0.3711
*IL6R*	rs2228145	D358A	C = 0.2931
*AIRE*	rs1800520	S278R	G = 0.2282
*TYK2*	rs34536443	P1104A	C = 0.0102
*RTKN2*	rs3125734	H462R	T = 0.4111
*PLD4*	rs2841280	E34Q	C = 0.4119
*NFKBIE*	rs2233434	V194A	G = 0.0669
	rs2233433	P175L	A = 0.0529
*SH2B3*	rs3184504	W262R	T = 0.1474
*CD226*	rs763361	S307G	C = 0.4694
*WDFY4*	rs7097397	R1816Q	A = 0.3586
*YDJC*	rs2298428	A263T	T = 0.2248
*PRKCH*	rs2230500	V374I	A = 0.0605

* MAF: minor allele frequency.

**Table 2:** SIFT, PROVEAN, PolyPhen2, SNP&GO, and PhD-SNP results for the selected nsSNPs.

Gene	SNP ID	PhD-SNP		SNP&GO		PolyPhen-2		PROVEAN		SIFT	
		Prediction	Score (Threshold 0.5)	Prediction	Score (Threshold 0.5)	Prediction	Score (0–1)	Prediction	Score (Threshold −2.5)	Prediction	TI Score (Threshold 0.05)
*PTPN22*	rs2476601	Neutral	0.473	Neutral	0.253	Benign	0.029	Deleterious	−5.099	Deleterious	0.03
*TNFAIP3*	rs5029941	Neutral	0.242	Neutral	0.071	Probably damaging	0.983	Neutral	−2.147	Deleterious	0.006
	rs2230926	Neutral	0.425	Neutral	0.222	Possibly damaging	0.515	Deleterious	−3.993	Tolerated	0.093
*TYK2*	rs34536443	Neutral	0.300	Neutral	0.094	Probably damaging	1.00	Deleterious	−6.755	Deleterious	0.007

**Table 3:** Ensembl results for the selected four nsSNPs.

Gene	SNP	CADD	REVEL	MetalR	Mutation Assessor
*PTPN22*	rs2476601	14	0.07	0.003	0.00
*TNFAIP3*	rs5029941	16	0.078	0.035	0.373
	rs2230926	18	0.153	0.025	0.294
*TYK2*	rs34536443	26	0.586	0.336	0.36

**Table 4:** Prediction of protein stability upon introduction of the nsSNP.

Gene	SNP ID	Stability	Torsion	Predicted ΔΔG (kcal/mol)
*PTPN22*	rs33996649	Destabilizing	Unfavorable	−0.116
	rs2476601	Destabilizing	Favorable	−6.98
*PADI4*	rs11203366	Destabilizing	Favorable	−5.91
	rs11203367	Destabilizing	Unfavorable	−0.46
*CTLA4*	rs231775	Destabilizing	Favorable	−1.04
*TNFAIP3*	rs5029941	Stabilizing	Unfavorable	2.78
	rs2230926	Destabilizing	Unfavorable	−4.58
*FCGR2A*	rs1801274	Destabilizing	Favorable	−1.19
*FCGR2B*	rs1050501	Destabilizing	Favorable	−0.91
*IRAK1*	rs1059703	Destabilizing	Unfavorable	−3.43
	rs1059702	Stabilizing	Unfavorable	0.16
*IL6R*	rs2228145	Stabilizing	Favorable	0.04
*AIRE*	rs1800520	Destabilizing	Favorable	−0.16
*TYK2*	rs34536443	Stabilizing	Favorable	6.73
*RTKN2*	rs3125734	Stabilizing	Unfavorable	1.79
*PLD4*	rs2841280	Stabilizing	Unfavorable	1.99
*NFKBIE*	rs2233434	Destabilizing	Favorable	−0.91
	rs2233433	Destabilizing	Favorable	−1.79
*SH2B3*	rs3184504	Stabilizing	Unfavorable	0.91
*CD226*	rs763361	Destabilizing	Favorable	−0.43
*WDFY4*	rs7097397	Destabilizing	Unfavorable	−0.09
*YDJC*	rs2298428	Stabilizing	Unfavorable	0.43
*PRKCH*	rs2230500	Destabilizing	Unfavorable	−0.91

**Table 5:** Results predicted by I-Mutant, MutPred, and ConSurf for the important nsSNPs.

Gene	SNP ID	I-Mutant (Stability)	MutPred		ConSurf Conservation Profile
			PROSITE and ELM Motifs	Molecular Mechanisms	
*PTPN22*	rs33996649	Decrease	None	None	Highly conserved, exposed, and functional residue
	rs2476601	Decrease	None	None	Highly conserved, exposed, and functional residue
*TNFAIP3*	rs2230926	Decrease	ELME000053, ELME000064, ELME000106, ELME000146, ELME000220, ELME000239,	1. Gain of intrinsic disorder 2. Loss of allosteric site at R123	Exposed
*TYK2*	rs34536443	Decrease	None	None	Highly conserved, exposed, and functional residue

**Table 6:** Percentage identity and coverage of the best-matching templates for query proteins.

Query Protein	Templates	Identity (%)	Coverage (%)	Query Protein	Templates	Identity (%)	Coverage (%)
PTPN22	3BRH	99.35	38	AIRE	2LRI	100	19
	4J51	100	37		1XWH	96.88	19
	3H2X	100	37		2KFT	100	17
	2P6X	99.67	37		4ZQL	49.18	21
TNFAIP3	3DKB	100	46	RTKN	4XH3	22.28	56
	5LRX	100	46		1UPQ	31.63	16
	2VFJ	100	46		2Y7B	22.22	17
	3ZJD	99.73	46		1WJM	31.58	9
TYK2	4OLI	98.57	53	PLD4	2ZE4	26.52	34
	4PO6	100	47		4GGJ	26.32	22
	3ZON	100	50		2ZE9	25.97	34
	5C01	100	49		1BYR	24.70	30
CTLA4	2 × 44	94.44	72	NFKBIE	1K1A	45.74	44
	1I85	94.44	72		1IKN	37.99	45
	5XJ3	94.44	72		1NFI	40	45
	3OSK	94.44	72		1OY3	38.05	37
FCGR2A	1FCG	99.43	54	SH2B3	5W3R	74.04	18
	1H9V	99.42	54		2HDV	71.30	18
	3D5O	99.42	53		1RQQ	69.23	18
	3RY4	99.41	53		1RPY	68.27	18
FCGR2B	5OCC	100	56	CD226	6ISB	100	69
	3WJJ	100	55		6ISA	53.39	65
	2FCB	99.42	55		5B22	26.21	59
	1H9V	99.19	55		4FQM	26.21	59
IRAK1	6BFN	99.71	47	PRKCH	3TXO	99.43	51
	6EG9	34.74	45		4RA4	57.57	49
	2NRY	34.74	45		3IW4	57.27	49
	2NRU	34.24	45		2I0E	58.11	49
IL6R	1N26	100	69	PADI4	3APM	100	100
	5FUC	99.06	54		4X8C	99.85	100
	1P9M	100	42		4DKT	99.55	100
	2ARW	100	26		3APN	99.55	100
WDFY4	1T77	46.72	11	YDJC	2I5I	37.23	40
	1MI1	46.17	11				
	5A1U	23.70	6				
	6G6M	30.30	5				

**Table 7:** Ramachandran plot analysis percentages of favored, allowed, and outlier residues for the modeled structures.

Protein	Favored (%)	Allowed (%)	Outlier (%)
PTPN22	85	9.4	5.6
PADI4	91.8	5.1	3.0
CTLA4	96.5	2.9	0.6
TNFAIP3	84.3	8.5	7.2
FCGR2A	95.2	4.4	0.3
FCGR2B	93.8	4.5	1.6
IRAK1	93.7	3.5	2.8
IL6R	88.4	7.7	3.9
AIRE	84.9	9.6	5.4
TYK2	82.1	9.8	8.1
RTKN2	83.5	10.5	5.9
PLD4	90.8	4.9	4.3
NFKBIE	91.6	6.6	1.8
SH2B3	83.2	10.6	6.1
CD226	94.9	3.8	1.4
WDFY4	78.5	13.0	8.5
YDJC	78.5	13.0	8.5
PRKCH	88.0	8.4	3.7

**Table 8:** Intronic SNP effect on splicing site as predicted by ESEfinder 3.0.

Gene	SNP ID	Potential Splicing Site	Gene	SNP ID	Potential Splicing Site
*PTPN22*	rs3765598	SRFSF2→ No Site	*AIRE*	rs2075876	SRSF1, SRSF2, SRSF5 → No Site
	rs1217414	SRSF2, SRSF5 → No Site		rs933150	No Site → SRSF2, SRSF6
*FCRL3*	rs3761959	SRSF5 → No Site	*TNFRSF14*	rs3890745	No Site → SRF5
*TRAF1/C5*	rs3761847	SRSF1, SRSF5 → No Site	*RUNX1*	rs2268277	SRSF1 → No Site
	rs2900180	No Site → SRSF5	*RASGRP1*	rs8043085	SRSF1, SRSF2, SRSF5 → No Site
*TNFAIP3*	rs5029930	SRSF1, SRSF5 → No Site	*ILF3*	rs147622113	SRSF1, SRSF2 → No Site
	rs5029937	No Site → SRSF2	*COG6*	rs9603612	SRSF1, SRSF2 → SRSF6
	rs5029939	SRSF2 → No Site		rs7993214	No Site → SRSF6
*STAT4*	rs7574865	No Site → SRSF2	*UBASH3A*	rs11203203	No Site → SRSF5
*IL2RB*	rs3218253	SRSF1 → SRSF5		rs3788013	No Site → SRSF6
*CD40*	rs4810485	No Site → SRSF5	*TEC*	rs2089510	No Site → SRSF2
	rs1535045	SRSF1 → No Site	*SYNGR1*	rs909685	No Site → SRSF6
	rs3765459	SRSF5 → No Site	*RAD51B*	rs3784099	SRSF2 → No Site
*CD244*	rs3766379	No Site → SRSF5		rs911263	SRSF1, SRSF2, SRSF5 → No Site
*TRAF6*	rs540386	SRSF2, SRSF6 → No Site	*PRKCH*	rs912620	No Site → SRSF2
	rs13031237	SRSF6 → SRSF5		rs959728	SRSF5, SRSF6 → No Site
*CD28*	rs2140148	SRSF1, SRSF5 → No Site		rs3783782	SRSF2 → No Site
*ANKRD55*	rs9295089	No Site → SRSF1 SRSF2	*PPIL4*	rs9498368	SRSF1 → No Site
	rs212402	SRSF2 → No Site	*PLCL2*	rs4535211	No Site → SRSF6
*IL6R*	rs4537545	SRSF1, SRSF2, SRSF5 → No Site	*MTF1*	rs67704103	SRSF1, SRSF5 → No Site
	rs4329505	No Site → SRSF2 SRSF5	*GATA3*	rs3802604	SRSF5 → SRSF1

**Table 9:** ESEfinder3.0 and HSF3.1 prediction for SNP rs2004640 located in the *IRF5* gene.

Method	Silencer/Enhancer Protein (Potential Splice Sites)	Motifs		Result
		G Allele (Value 0–100)	T Allele (Value 0–100)	
Human Splicing Finder 3.1 (Threshold 60)		-	CGGgtgggt (85.64)	New site (position −4 bp)
	Enhancer motifs SF2/ASF (IgM-BRCA1)	CGGGGGG (78.92)	-	Site broken at position −4 bp
	Silencer motifs (Sironi et al.)	Motif 2 CTCGGGG (60.84)	-	Site broken at position −7 bp
		Motif 2 TCGGGGG (70.71)	-	Site broken at position −5 bp
		Motif 2 GGGGGTG (67.64)	-	Site broken at position −1 bp
		-	Motif 2 TGGGTGC (60.69)	New site at SNP position
	Silencer IIEs motifs (Zhang et al.)	CGGGGG	-	Site broken at −4 bp position
ESEfinder 3.0 (Threshold 1.867)	SRSF2 (IgM-BRCA1)	CGGGGGG (2.95482)	-	Site broken at position −4 bp

**Table 10:** UTR SNPs associated with RA and their predictive regulatory role.

Gene	SNPs	UTRScan	PolymiRTS Database	MicroSNiPer
PTPN22	rs3811021	-	hsa-miR-4275 → hsa-miR-548ad	hsa-miR-4275
TAGAP	rs4709267	-	-	hsa-miR-4696, hsa-miR-548u
IRF5	rs2070197	-	hsa-miR-3136-3p, hsa-miR-7155-3p → no site	hsa-miR-3136-3p, hsa-miR-1295b-5p
	rs10954213	-	-	hsa-miR-181b-5p, hsa-miR-181d, hsa-miR-181a-5p, hsa-miR-181c-5p
ETS1	rs1128334	No site → BRD-BOX	hsa-miR-300, hsa-miR-381-3p, hsa-miR-6882-5p → hsa-miR-382-5p, hsa-miR-495-5p	hsa-miR-4528

## Data Availability

All the data are presented in this manuscript, either in the main article or in the Supplementary Materials.

## Supplementary Materials

The following are available online at https://www.mdpi.com/article/10.3390/biology10060501/s1, Figure S1: ConSurf results for all the proteins with missense RA-associated SNPs. These figures were downloaded as a PDF file from the ConSurf web server (https://consurf.tau.ac.il/, accessed on 15 January 2021). Figure S2: Guidelines for the in-depth understanding of STRING results. Table S1: List of SNPs associated with rheumatoid rrthritis. Table S2: Clinical associations of reported SNPs with RA patients.
