# Supplementary material for: Characterization of Rheumatoid Arthritis Risk-Associated SNPs and Identification of Novel Therapeutic Sites Using an In-Silico Approach
Source: Biology (Basel). 2021 Jun 4;10(6):501. doi: 10.3390/biology10060501 (PMC8227790; doi:10.3390/biology10060501)
Supplement: Supplementary file 1 [file biology-10-00501-s001.zip › S1 fig.pdf]

# ConSurf Results

```
1          11         21         31         41
MATDAALRRL LRLHRTEIAV AVDSAFPLLH ALADHDVVPE DKFQETLHLK
eeeeeebeeb bebeeeebbb bbbebbebbe bbeeeeebee eebeeebeee
 f f   f   s   fffs   sssf sfssf  sfffff        ff

51         61         71         81         91
EKEGCPQAFH ALLSWLLTQD STAILDFWRV LFKDYNLERY GRLQPILDSF
eeeebeebbe bbbbbbbeee bebbbbbbeb bbeeeebeeb bebeebeeeb
  ff ffs f  ss  ss  f       ss   s ffff ffs  fs

101        111        121        131        141
PKDVDLSQPR KGRKPPAVPK ALVPPPRLPT KRKASEEARA AAPAALTPRG
eeebeeeeee eeeeeeeeee beeeeeeeee eeeeeeeeee eeeeeeeeee
f f f        f                   ff    f

151        161        171        181        191
TASPGSQLKA KPPKKPESSA EQQRLPLGNG IQTMSASVQR AVAMSSGDVP
eeeeeeeeee eeeeeeeeee eeeebeeeeb bebbbeebee bbbbbeeebe
  f f   f  f   f                  f    fs f      f

201        211        221        231        241
GARGAVEGIL IQQVFESGGS KKCIQVGGEF YTPSKFEDSG SGKNKARSSS
ebebbbebbb beebbeeeee eebbeeeeeb eeeeeeeeee eeeeeeeeee
   s  f      f   f         ff          f

251        261        271        281        291
GPKPLVRAKG AQGAAPGGGE ARLGQQGSVP APLALPSDPQ LHQKNEDECA
ebeeebeeee eeeeeeeeee eeeeeeeeee eeeeeeeeee eeeeeeeebb
  f   f     f                                 f ffffs

301        311        321        331        341
VCRDGGELIC CDGCPRAFHL ACLSPPLREI PSGTWRCSSC LQATVQEVQP
bbeeeeebbb beeeeebbbb bbbeeebeeb eebebebeeb beeeeeeeee
 s ffffs s sfffffs ss  s  ff     ffs sfs  s

351        361        371        381        391
RAEEPRPQEP PVETPLPPGL RSAGEEVRGP PGEPLAGMDT TLVYKHLPAP
eeeeeeeeee eeeeebbebb eeeeeebeee eeeeeeeeeb ebbbeebeee

401        411        421        431        441
PSAAPLPGLD SSALHPLLCV GPEGQQNLAP GARCGVCGDG TDVLRCTHCA
eeeeebeebe eeebeebbbe eeeeeeeeee eeebbebeee eebbebbebb
    f

451        461        471        481        491
AAFHWRCHFP AGTSRPGTGL RCRSCSGDVT PAPVEGVLAP SPARLAPGPA
eebebebebe eeeeeeeeeb ebeebeeeee eeeeeeeeee eeeeeeeeee

501        511        521        531        541
KDDTASHEPA LHRDDLESLL SEHTFDGILQ WAIQSMARPA APFPS
eeeeeeeeeb beeeebeebb eeebbebbbb bbbeebeeee eeeee
             f fsffss  f   fsss   s ffs ff
```

**The conservation scale:**

| 1 | 2 | 3 | 4 | 5 | 6 | 7 | 8 | 9 |
|---|---|---|---|---|---|---|---|---|

Variable Average Conserved

e - An exposed residue according to the neural-network algorithm.

b - A buried residue according to the neural-network algorithm.

f - A predicted functional residue (highly conserved and exposed).

s - A predicted structural residue (highly conserved and buried).

X - Insufficient data - the calculation for this site was performed on less than 10% of the sequences.

# ConSurf Results

```
1                    11                   21                   31                   41
M D Y P T L L L A L  L H V Y R A L C E E  V L W H T S V P F A  E N M S L E C V Y P  S M G I L T Q V E W
e e e b e b b b b b  b e b b e e b b e e  e b e e b b b e b b  e e b e b e b b b e  e e e e b e e b e b
f                              f     f          f                      s   s                  f f   f s

51                   61                   71                   81                   91
F K I G T Q Q D S I  A I F S P T H G M V  I R K P Y A E R V Y  F L N S T M A S N N  M T L F F R N A S E
b e e b e e e e b b  b b b b b e b b b b  b e e e b e e e b b  b b e b e e e e e e  b b b b b e e b e e
  f                          s                    s                f                    s   s   f   f

101                  111                  121                  131                  141
D D V G Y Y S C S L  Y T Y P Q G T W Q K  V I Q V V Q S D S F  E A A V P S N S H I  V S E P G K N V T L
e e b b b b b b b b  b b b e e e e b e e  b b e b b e e e e b  e b e e e e e e e b  b e e e e e e b e b
  f   s   s   s            f   f       f        s           s                               f   f   f

151                  161                  171                  181                  191
T C Q P Q M T W P V  Q A V R W E K I Q P  R Q I D L L T Y C N  L V H G R N F T S K  F P R Q I V S N C S
e b e e e e e e e b  e e b e b e e b e e  e e b e b b b b b e  e e e e e e e e e e  b e e e b b e e b e
                             s f   s f      f   f         s f                  f                    s

201                  211                  221                  231                  241
H G R W S V I V I P  D V T V S D S G L Y  R C Y L Q A S A G E  N E T F V M R L T V  A E G K T D N Q Y T
e e b e b b b b b b  b b b b e e e e b b  e b e b e e e e e e  e e e b b b e b b b  e e e e e e e e b b
                             f f f f   s  f s             f        f

251                  261                  271                  281                  291
L F V A G G T V L L  L L F V I S I T T I  I V I F L N R R R R  R E R R D L F T E S  W D T Q K A P N N Y
b b b b b b b b b b  b b b b b b b b b b  b b b b b e e e e e  e e e e e e b e e e  e e e e e b e e e e
          s                                                 f  f                            f       f

301                  311                  321                  331
R S P I S T S Q P T  N Q S M D D T R E D  I Y V N Y P T F S R  R P K T R V
e e e e e e e e e e  e e e e e e e e e e  b b b e b e e b e e  e e e e e e
    f                    f           f f    s s f   f            f
```

## The conservation scale:

| 1 | 2 | 3 | 4 | 5 | 6 | 7 | 8 | 9 |
|---|---|---|---|---|---|---|---|---|

Variable | Average | Conserved

e - An exposed residue according to the neural-network algorithm.
b - A buried residue according to the neural-network algorithm.
f - A predicted functional residue (highly conserved and exposed).
s - A predicted structural residue (highly conserved and buried).
X - Insufficient data - the calculation for this site was performed on less than 10% of the sequences.

# ConSurf Results

```
1                      11                     21                     31                     41
M A C L G F Q R H K    A Q L N L A T R T W    P C T L L F F L L F    I P V F C K A M H V    A Q P A V V L A S S
e e e e e b e e e e    b e b e e b e e e b    e b b b b b b b b b    b b b b b e b b e b    b b e b b b b b e e
f                                                                                      s      s f

51                     61                     71                     81                     91
R G I A S F V C E Y    A S P G K A T E V R    V T V L R Q A D S Q    V T E V C A A T Y M    M G N E L T F L D D
e e b b e b b b e b    e e e e e b e e b e    b b b b e e e e e e    b e e b b b b e b e    e e e e b e b e e e
  f   s       s                      f   f      s s   f                      s s

101                    111                    121                    131                    141
S I C T G T S S G N    Q V N L T I Q G L R    A M D T G L Y I C K    V E L M Y P P P Y Y    L G I G N G T Q I Y
e e b e e e e e e e    e b e b b b e b b e    b e e e e b b b b e    b e e b b e b e b b    b b e b e e b e b b
    s   f                s f   s     s            f   f   s   s        f f     f s f s            s f f s   s

151                    161                    171
V I A K E K K P S Y    N R G L C E N A P N    R A R M
b b e e e e e e e e    e e e e b e e b e e    e e e e
s       f                f   f   f f s        f f f
```

## The conservation scale:

| 1 | 2 | 3 | 4 | 5 | 6 | 7 | 8 | 9 |
|---|---|---|---|---|---|---|---|---|
| Variable | | | | Average | | | Conserved | |

e - An exposed residue according to the neural-network algorithm.

b - A buried residue according to the neural-network algorithm.

f - A predicted functional residue (highly conserved and exposed).

s - A predicted structural residue (highly conserved and buried).

X - Insufficient data - the calculation for this site was performed on less than 10% of the sequences.

# ConSurf Results

```
1                     11                    21                    31                    41
M T M E T Q M S Q N   V C P R N L W L L Q   P L T V L L L L A S   A D S Q A A A P P K   A V L K L E P P W I
e e e e e e b b e e   b e e e e b b b b b   e b b b b b b b b e   e e e e e e e b e e   b b b e b e e e b b
f f                                                                                 f   s           f f

51                    61                    71                    81                    91
N V L Q E D S V T L   T C Q G A R S P E S   D S I Q W F H N G N   L I P T H T Q P S Y   R F K A N N N D S G
e b b e e e e b e b   e b e e e e e e e e   e e e e e b e e b e   e b e e e e e e e b   e b e b e e e e e e
  s       f   s f s     s   f                   f   f   f f               f       f                   f f

101                   111                   121                   131                   141
E Y T C Q T G Q T S   L S D P V H L T V L   S E W L V L Q T P H   L E F Q E G E T I M   L R C H S W K D K P
e b e b e e e e e e   e e e e b e b e b b   b e b b b b e b b e   b e b e e e e e b b   b b b e e e e e e e
  s   s               f f f f s   s   s         s s   s f s                 f           s s s f f f

151                   161                   171                   181                   191
L V K V T F F Q N G   K S Q K F S H L D P   T F S I P Q A N H S   H S G D Y H C T G N   I G Y T L F S S K P
b b e b b b b e e e   e e e e b e e e e e   e b b b e e b e e e   e e e e e e b e b e   e b e e e e e e e e
    f           f                                 s     s f       f f f   f   s                       f

201                   211                   221                   231                   241
V T I T V Q V P S M   G S S S P M G I I V   A V V I A T A V A A   I V A A V V A L I Y   C R K K R I S A N S
b e b b b e e e e e   e e e b b b b b b b   b b b b b b b b b b   b b b b b b b b b e   b e e e e e e e e e
s   s

251                   261                   271                   281                   291
T D P V K A A Q F E   P P G R Q M I A I R   K R Q L E E T N N D   Y E T A D G G Y M T   L N P R A P T D D D
e e e e e e e e e e   e e e e b b b b b e   e e e e e e e e e e   e e e e e e e e b e   b e e e e e e e e e
                                                                          f         f                     f

301                   311
K N I Y L T L P P N   D H V N S N N
e e b b b b b e e e   e e e e e e e
  f
```

**The conservation scale:**

| 1 | 2 | 3 | 4 | 5 | 6 | 7 | 8 | 9 |
|---|---|---|---|---|---|---|---|---|
| Variable | | | | Average | | | | Conserved |

e - An exposed residue according to the neural-network algorithm.

b - A buried residue according to the neural-network algorithm.

f - A predicted functional residue (highly conserved and exposed).

s - A predicted structural residue (highly conserved and buried).

X - Insufficient data - the calculation for this site was performed on less than 10% of the sequences.

# ConSurf Results

```
1                    11                   21                   31                   41
M G I L S F L P V L  A T E S D W A D C K  S P Q P W G H M L L  W T A V L F L A P V  A G T P A A P P K A
e e b e e b b b b b  e e e e e e b e b e  e e e e e b b b b b  b b b b b b b b e e  e e e e e e b e e b
f                                                                                                   f

51                   61                   71                   81                   91
V L K L E P Q W I N  V L Q E D S V T L T  C R G T H S P E S D  S I Q W F H N G N L  I P T H T Q P S Y R
b b e b e e e e b b e  b b e e e e b e b e  b e e e e e e e e e  e e e b b e e e e e  b e e e e e e e e b
          f                  f   s f s      s   f                f f   s   f f

101                  111                  121                  131                  141
F K A N N N D S G E  Y T C Q T G Q T S L  S D P V H L T V L S  E W L V L Q T P H L  E F Q E G E T I V L
b e b e e e e e e e  e e b e e e e e e e  e e e b e b e b b e  e b b b b e b b e e  e b e e e e e b b b
              f f    f   s                f   f     s   s        s s   s f                f f         s

151                  161                  171                  181                  191
R C H S W K D K P L  V K V T F F Q N G K  S K K F S R S D P N  F S I P Q A N H S H  S G D Y H C T G N I
e b e e b e e e e b  b e b b b b e e e e  e e e b e e e e e e  b e b e e b e e e e  e b e e e b e b e e
f s f       f                                                      s     s       f    s   f   s

201                  211                  221                  231                  241
G Y T L Y S S K P V  T I T V Q A P S S S  P M G I I V A V V T  G I A V A A I V A A  V V A L I Y C R K K
e e e e e e e e e b  e b b b e e e e e e  e b b b b b b b b b  b b b b b b b b b b  b b b b b e b e e e
            f

251                  261                  271                  281                  291
R I S A L P G Y P E  C R E M G E T L P E  K P A N P T N P D E  A D K V G A E N T I  T Y S L L M H P D A
e e e e e e e e e e  e e e e e e e e e e  e e e e b e e e e e  b e e e e e e e e b  b b b b b e b e e e
                       f             f                                      f f      s s s s s   s f

301
L E E P D D Q N R I
e e e e e e e e e e
                  f
```

**The conservation scale:**

| 1 | 2 | 3 | 4 | 5 | 6 | 7 | 8 | 9 |
|---|---|---|---|---|---|---|---|---|

Variable    Average    Conserved

- e - An exposed residue according to the neural-network algorithm.
- b - A buried residue according to the neural-network algorithm.
- f - A predicted functional residue (highly conserved and exposed).
- s - A predicted structural residue (highly conserved and buried).
- X - Insufficient data - the calculation for this site was performed on less than 10% of the sequences.

# ConSurf Results

```
1            11           21           31           41
MLAVGCALLA   ALLAAPGAAL   APRRCPAQEV   ARGVLTSLPG   DSVTLTCPGV
eeebebbbbb   bbbebeeeee   eeeebeeeeb   eeebbbeeee   bebbbebeee
f                                               f       s s

51           61           71           81           91
EPEDNATVHW   VLRKPAAGSH   PSRWAGMGRR   LLLRSVQLHD   SGNYSCYRAG
eeeeeebebb   bbeeeeeeee   eeeeeeebee   bbbeebeeee   eeeebbeeee
         s                               s      f   ff f s

101          111          121          131          141
RPAGTVHLLV   DVPPEEPQLS   CFRKSPLSNV   VCEWGPRSTP   SLTTKAVLLV
eeeebbebbb   eeeeeeeebe   beeeeeeeeb   bbeeeeeeee   eeebebbbbb
   f   s s     f f f      s f f         sff      f  f  s s s

151          161          171          181          191
RKFQNSPAED   FQEPCQYSQE   SQKFSCQLAV   PEGDSSFYIV   SMCVASSVGS
eeeeeeeeee   eeeebeeeee   eeebebebee   eeeeeebbbb   bbbbbeeeee
                 s f         s s          f           s  f  f

201          211          221          231          241
KFSKTQTFQG   CGILQPDPPA   NITVTAVARN   PRWLSVTWQD   PHSWNSSFYR
eeeeeeebeb   eebbeeeeee   ebebebbeee   eeebebebee   eeeeeeebbe
  f               ffff    f  s         f  s s s     f fff   s

251          261          271          281          291
LRFELRYRAE   RSKTFTTWMV   KDLQHHCVIH   DAWSGLRHVV   QLRAQEEFGQ
bebebeeeee   eeeebbebeb   eeeeeebbbe   bbbeebeebb   ebebeeebee
s sf fffff                        s    ss  f  f s   f fs ff

301          311          321          331          341
GEWSEWSPEA   MGTPWTESRS   PPAENEVSTP   MQALTTNKDD   DNILFRDSAN
bebeebbeeb   eeeeeeeeee   eeeeeeeeee   eeeeeeeeee   eeeeeeeeee
s  f  s f     f ff

351          361          371          381          391
ATSLPVQDSS   SVPLPTFLVA   GGSLAFGTLL   CIAIVLRFKK   TWKLRALKEG
eeeeeeeeee   eeebbbbbbb   bbbbbbbbbb   bbbbbbebee   ebeeebeeee
                          s s   s  s         f

401          411          421          431          441
KTSMHPPYSL   GQLVPERPRP   TPVLVPLISP   PVSPSSLGSD   NTSSHNRPDA
eeeeeeeebb   eeebeeeeee   eebebbbbeb   eeeeeeeeee   eeeeeeeeee
f     f       f   f f f        s  f      f  f  f

451          461
RDPRSPYDIS   NTDYFFPR
eeeeeeeebe   eeeeeeee
       f f   f ffff
```

**The conservation scale:**

| 1 | 2 | 3 | 4 | 5 | 6 | 7 | 8 | 9 |
|---|---|---|---|---|---|---|---|---|
| Variable | | | | Average | | | | Conserved |

e - An exposed residue according to the neural-network algorithm.

b - A buried residue according to the neural-network algorithm.

f - A predicted functional residue (highly conserved and exposed).

s - A predicted structural residue (highly conserved and buried).

# ConSurf Results

```
1           11          21          31          41
MAGGPGPGEP  AAPGAQHFLY  EVPPWVMCRF  YKVMDALEPA  DWCQFAALIV
eeeeeeeeee  eeeebeebbe  ebeeebbbeb  bebbeebeeb  ebbebbbebb
                          f      s     sffs     fs  sss s 

51          61          71          81          91
RDQTELRLCE  RSGQRTASVL  WPWINRNARV  ADLVHILTHL  QLLRARDIIT
eeebebebbe  eeeeeeeebb  bebbeeeeeb  bebbebbeeb  eebebeebbb
 ff   fs        ff        s  fff s    s   s  s     f  f   

101         111         121         131         141
AWHPPAPLPS  PGTTAPRPSS  IPAPAEAEAW  SPRKLPSSAS  TFLSPAFPGS
ebeeeeeeee  eeeeeeeeee  eeeeeeeeee  eeeeeeeeee  ebebeebeee
                   f                                      

151         161         171         181         191
QTHSGPELGL  VPSPASLWPP  PPSPAPSSTK  PGPESSVSLL  QGARPFPFCW
eeeeeeeeee  eeeeeeeeee  eeeeeeeeee  eeeeeeeebb  eeeeeeebbb
                                                         s

201         211         221         231         241
PLCEISRGTH  NFSEELKIGE  GGFGCVYRAV  MRNTVYAVKR  LKENADLEWT
ebeebeeeee  ebeeebebee  ebbbbbbebe  beebeebbee  beeebeeebe
   f   ff    sf     ff  fsss ss s     fs fssf    f     f  

251         261         271         281         291
AVKQSFLTEV  EQLSRFRHPN  IVDFAGYCAQ  NGFYCLVYGF  LPNGSLEDRL
ebeebbeeeb  eebeebebee  bbbbbbbbee  eebbbbbbbb  beeeebeeeb
    s  ffs    sf  fsff  s s  s          ssss    s ffsff  s

301         311         321         331         341
HCQTQACPPL  SWPQRLDILL  GTARAIQFLH  QDSPSLIHGD  IKSSNVLLDE
eeeeeeeeee  ebeeebebbe  bbbebbebbe  eeeeebbebe  bebbebbbee
         f      f         s s f sf        sfsf   fssfsssf 

351         361         371         381         391
RLTPKLGDFG  LARFSRFAGS  SPSQSSMVAR  TQTVRGTLAY  LPEEYIKTGR
ebeeebeebb  bbebbeeeee  eeeeeeeebe  eeebebbbee  beeebbeeee
f  ffsffss  ssf             f       f f fsssff  sf fs f f 

401         411         421         431         441
LAVDTDTFSF  GVVVLETLAG  QRAVKTHGAR  TKYLKDLVEE  EAEEAGVALR
bbbebbbbbb  bbbbbebbee  eebbeeeeee  eebbeebbee  eeeeeeeeee
s    s  ss  sss sf sff  f s            sffs               

451         461         471         481         491
STQSTLQAGL  AADAWAAPIA  MQIYKKHLDP  RPGPCPPELG  LGLGQLACCC
eeeeebeeeb  eeebebbebb  eebbeeebee  eeeeeeeebb  eebbebbbeb
                                f                     s  s

501         511         521         531         541
LHRRAKRRPP  MTQVYERLEK  LQAVVAGVPG  HSEAASCIPP  SPQENSYVSS
beeeeeeeee  beebbeebee  beeebeeeee  eeeeeeeeee  eeeeeeeeee
s  f f ff   s  s   s                             fff f  f 

551         561         571         581         591
TGRAHSGAAP  WQPLAAPSGA  SAQAAEQLQR  GPNQPVESDE  SLGGLSAALR
eeeeeeeeee  eeeeeeeeee  eeebeeeeee  eeeeeeeeee  eeeebbbbbb
                                        fffff   f         
```

| Position | Residues | Exposure | Function/Structure |
|---|---|---|---|
| 601 | S W H L T P S C P L | b b e b e e e e e e | |
| 611 | D P A P L R E A G C | e e e e e e e e e e | |
| 621 | P Q G D T A G E S S | e e e e e e e e e e | |
| 631 | W G S G P G S R P T | e e e e e e e e e e | |
| 641 | A V E G L A L G S S | e e e e e e e e e e | |
| 651 | A S S S S E P P Q I | e e e e e e e e e b | s (I) |
| 661 | I I N P A R Q K M V | b b b e b e e e b b | _ _ s f _ f |
| 671 | Q K L A L Y E D G A | e e b b b e e e e b | _ f _ _ _ f _ _ f |
| 681 | L D S L Q L L S S S | b e b b e b b e e e | s _ s _ f s _ f f |
| 691 | S L P G L G L E Q D | e b e e e e e e e e | |
| 701 | R Q G P E E S D E F | e e e e e e e e e e | _ _ _ f f f f f f |
| 711 | Q S | e e | _ f |

**The conservation scale:**

| 1 | 2 | 3 | 4 | 5 | 6 | 7 | 8 | 9 |
|---|---|---|---|---|---|---|---|---|
| Variable | | | | Average | | | | Conserved |

e - An exposed residue according to the neural-network algorithm.
b - A buried residue according to the neural-network algorithm.
f - A predicted functional residue (highly conserved and exposed).
s - A predicted structural residue (highly conserved and buried).
X - Insufficient data - the calculation for this site was performed on less than 10% of the sequences.

# ConSurf Results

```
1           11          21          31          41
MNQRRSESRP  GNHRLQAYAE  PGKGDSGGAG  PLSGSARRGR  GGGGAIRVRR
eeeeeeeeee  eebebbbbbe  eeeeeeeeee  beeeeeeebe  eeeeeeebeb

51          61          71          81          91
PCWSGGAGRG  GGPAWAVRLP  TVTAGWTWPA  LRTLSSLRAG  PSEPHSPGRR
eeebeebbee  ebeebbeebe  ebbeeeeeeb  beeeeeeeee  eeeeeeeeee

101         111         121         131         141
PPRAGRPLCQ  ADPQPGKAAR  RSLEPDPAQT  GPRPARAAGM  SEARKGPDEA
eeeeeeeeee  eeeeeeeeee  eeeeeeeeee  eeeeeeeeee  eeeeeeeeee
                 f

151         161         171         181         191
EESQYDSGIE  SLRSLRSLPE  STSAPASGPS  DGSPQPCTHP  PGPVKEPQEK
eeeebeeeee  bbeebeeeee  eeeeeeeeee  eeeeeeeeee  eeeeeeeeee
     fff       f

201         211         221         231         241
EDADGERADS  TYGSSSLTYT  LSLLGGPEAE  DPAPRLPLPH  VGALSPQQLE
eeeeeeebee  eeeeeebbeb  beebeeeeee  eeeeeeeeee  eeeeeeeeee
     ff ff   f fff

251         261         271         281         291
ALTYISEDGD  TLVHLAVIHE  APAVLLCCLA  LLPQEVLDIQ  NNLYQTALHL
bbebbeeeee  bbbbbbbbee  eeebbebbbe  ebeeebbebe  eebeebbbeb
     fffff  s  sss sf                 f   sf f  ffs f  sfs

301         311         321         331         341
AVHLDQPGAV  RALVLKGASR  ALQDRHGDTA  LHVACQRQHL  ACARCLLEGR
bbbbeeeebb  ebbbeeebee  ebeeeeeeeb  bbbbbeeeee  ebbeebeeee
s s  f   s    s   f f     fff fff    s ssf f     s   sf

351         361         371         381         391
PEPGRGTSHS  LDLQLQNWQG  LACLHIATLQ  KNQPLMELLL  RNGADIDVQE
eeeeeeeeee  eebebeebee  bbbbbbbebe  eeeebbebbb  eeebebebee
             f   ff ff      s sf            s      sf f ff

401         411         421         431         441
GTSGKTALHL  AVETQERGLV  QFLLQAGAQV  DARMLNGCTP  LHLAAGRGLM
eeeeebbbeb  bbeeeeeebb  ebbbeeebeb  ebebbbbbbb  bbbbbbeeee
fffffs sf   ssf ff   s    ss  fs s  fs s ss s   ssss sf f

451         461         471         481         491
GISSTLCKAG  ADSLLRNVED  ETPQDLTEES  LVLLPFDDLK  ISGKLLLCTD
ebbebbbeee  beebbeebee  eebeebbeeb  bebbebeebe  beeeebebee
     s   f  sf    f ff  ffsff  f        fsffsf  sffff    f
```

**The conservation scale:**

| 1 | 2 | 3 | 4 | 5 | 6 | 7 | 8 | 9 |
|---|---|---|---|---|---|---|---|---|

Variable Average Conserved

e - An exposed residue according to the neural-network algorithm.
b - A buried residue according to the neural-network algorithm.
f - A predicted functional residue (highly conserved and exposed).
s - A predicted structural residue (highly conserved and buried).
X - Insufficient data - the calculation for this site was

**performed on less than 10% of the sequences.**

# ConSurf Results

```
1
MAQGTLIRVT
eeeeebbebe
ff

11
PEQPTHAVCV
eeeeeebbbb

21
LGTLTQLDIC
bbbebbbebe

31
SSAPEDCTSF
eeeeeebeeb
   f

41
SINASPGVVV
ebeeeeeebeb

51
DIAHGPPAKK
ebbeeeeeee

61
KSTGSSTWPL
eeeeeeebee
         f

71
DPGVEVTLTM
eeebebbbeb

81
KVASGSTGDQ
eeeeeeeeee
   f

91
KVQISYYGPK
ebebebeeee
f

101
TPPVKALLYL
eeeeebbbbb

111
TGVEISLCAD
bbbebbbebe
s     s  f

121
ITRTGKVKPT
beeeeeeeee
  f f

131
RAVKDQRTWT
eeeeeeeeee
        f

141
WGPCGQGAIL
ebeeeebbbb
fs  f ss

151
LVNCDRDNLE
bbbbeeeeee
ssssf

161
SSAMDCEDDE
eeeeeeeeee
    f

171
VLDSEDLQDM
beeeeebeeb
     f  fs

181
SLMTLSTKTP
bebbbeeeee
s   s   ff

191
KDFFTNHTLV
eebbeebebb

201
LHVARSEMDK
bebbeeeeee

211
VRVFQATRGK
bebbbbeeee
  s

221
LSSKCSVVLG
eeeebeebbe
        s

231
PKWPSHYLMV
eeeeeeebee

241
PGGKHNMDFY
eeeeeebebb

251
VEALAFPDTD
bebbebeeee
 f  sff

261
FPGLITLTIS
bebbbbbbbb
s ss

271
LLDTSNLELP
bbeeeeeebe
s

281
EAVVFQDSVV
ebebbeeebb
    sff s

291
FRVAPWIMTP
bbbbbbbbbe
s  ss sssf

301
NTQPPQEVYA
eeeeeeebbb
ff  f    s

311
CSIFENEDFL
bebeeeeebb
     f  s

321
KSVTTLAMKA
eebeebbeee
         f

331
KCKLTICPEE
ebebebbeee

341
ENMDDQWMQD
eeeeeebbee
 f  ffs ff

351
EMEIGYIQAP
ebebbbbeee
f f ss  ff

361
HKTLPVVFDS
eeebebbbbe
f   fss sf

371
PRNRGLKEFP
eeeeebeebe
ff

381
IKRVMGPDFG
beebebeebe
    fsff f

391
YVTRGPQTGG
bbeeeeeeee
ssf

401
ISGLDSFGNL
beebeebeeb
    ffsffs

411
EVSPPVTVRG
eeeeebebee
fffffs

421
KEYPLGRILF
eeeebbbbbb
  ffssss

431
GDSCYPSNDS
bbbebeeeee
s    f   f

441
RQMHQALQDF
eebeebbeeb
f s   s  s

451
LSAQQVQAPV
beeeebeeeb
s ff sf f

461
KLYSDWLSVG
ebbbebbbbb
   sfss  s

471
HVDEFLSFVP
bbbebbebbe
s sfs  s f

481
APDRKGFRLL
eeeeeebebb
  f f sf s

491
LASPRSCYKL
bbbeebbbeb
 ssf     s

501
FQEQQNEGHG
beeeeeeeee
       f f

511
EALLFEGIKK
ebebbeebee

521
KKQQKIKNIL
eeeeebeebb
         s

531
SNKTLREHNS
eeeebeeeee
        f

541
FVERCIDWNR
ebeebbebbe
  f ssf sf

551
ELLKRELGLA
ebbeeebeee
  sf fsff

561
ESDIIDIPQL
eeebbebeeb
  fs f ffs

571
FKLKEFSKAE
bebeeeeebe
s       s

581
AFFPNMVNML
bbbeebbbbb
s sff s

591
VLGKHLGIPK
bbbeebebee
ss   sfsff
```

601

| | | | | | | | | | |
|---|---|---|---|---|---|---|---|---|---|
| P | F | G | P | V | I | N | G | R | C |
| e | e | e | e | e | b | e | b | e | b |
| f | f | f | f | | | | | | s |

611

| | | | | | | | | | |
|---|---|---|---|---|---|---|---|---|---|
| C | L | E | E | K | V | C | S | L | L |
| b | b | e | e | e | b | e | e | b | b |
| | s | f | | | | | | s | |

621

| | | | | | | | | | |
|---|---|---|---|---|---|---|---|---|---|
| E | P | L | G | L | Q | C | T | F | I |
| e | e | b | e | b | e | b | b | b | b |
| | f | s | f | | | s | | s | |

631

| | | | | | | | | | |
|---|---|---|---|---|---|---|---|---|---|
| N | D | F | F | T | Y | H | I | R | H |
| b | e | b | b | e | b | b | e | e | e |
| | f | | | | s | s | | | |

641

| | | | | | | | | | |
|---|---|---|---|---|---|---|---|---|---|
| G | E | V | H | C | G | T | N | V | R |
| b | e | b | b | b | e | e | e | b | e |
| s | | | s | | f | f | f | s | |

651

| | | | | | | | | | |
|---|---|---|---|---|---|---|---|---|---|
| R | K | P | F | S | F | K | W | W | N |
| e | e | e | b | e | b | e | b | b | e |
| f | | f | | | | | s | s | |

661

| | | |
|---|---|---|
| M | V | P |
| e | e | e |
| | | f |

**The conservation scale:**

| 1 | 2 | 3 | 4 | 5 | 6 | 7 | 8 | 9 |
|---|---|---|---|---|---|---|---|---|
| Variable | | | | Average | | | Conserved | |

e - An exposed residue according to the neural-network algorithm.
b - A buried residue according to the neural-network algorithm.
f - A predicted functional residue (highly conserved and exposed).
s - A predicted structural residue (highly conserved and buried).
X - Insufficient data - the calculation for this site was performed on less than 10% of the sequences.

# ConSurf Results

```
1          11         21         31         41
MPGCPVQMLK PLWKAAVAPT WPCSMPPRRP WDREAGTLQV LGALAVLWLG
eeeeeeeebe ebeebeeeee bebeeeeeee eeeeeeebeb bebbbbbbbb

51         61         71         81         91
SVALICLLWQ VPRPPTWGQV QPKDVPRSWE HGSSPAWEPL EAEARQQRDS
bbbbbbbbee beeeeebeee eeeeeeeeee eeeeeeeeee eeeeeeeeee

101        111        121        131        141
CQLVLVESIP QDLPSAAGSP SAQPLGQAWL QLLDTAQESV HVASYYWSLT
bebbbbeebe eebebeeeee eeeebbebbb ebbeebeeeb ebbbbbbbbe
      ff f                        s   s

151        161        171        181        191
GPDIGVNDSS SQLGEALLQK LQQLLGRNIS LAVATSSPTL ARTSTDLQVL
eeeeeeeeee eeebeebbee beebeeeebe bebbeeeeee eeeeeebeeb
              s                      ff          f   s

201        211        221        231        241
AARGAHVRQV PMGRLTRGVL HSKFWVVDGR HIYMGSANMD WRSLTQVKEL
eeeeeebeeb ebeebbeebb bbebbbbeee bbbbbbbebe bebbeebeeb
    f s           f   ssf    f   s s sssf f sf sff ff

251        261        271        281        291
GAVIYNCSHL AQDLEKTFQT YWVLGVPKAV LPKTWPQNFS SHFNRFQPFH
bbbbeebebb beebeebbeb bbbbeeeeee beeeeeeebe eebeeeeebe
s    fsf     f  f s   s           f  ff     f  f   f

301        311        321        331        341
GLFDGVPTTA YFSASPPALC PQGRTRDLEA LLAVMGSAQE FIYASVMEYF
bebeeeebeb bbeeeeeebb eeeeeeebeb bbebbeebee bbbbbbbbbb
   f         ffff        f  f           s        ss s

351        361        371        381        391
PTTRFSHPPR YWPVLDNALR AAAFGKGVRV RLLVGCGLNT DPTMFPYLRS
eebeeeeeee bbeebeebbe ebbbeeebeb ebbbbbbeee eeebbebbeb
f           sf sf   f                 s    f          s

401        411        421        431        441
LQALSNPAAN VSVDVKVFIV PVGNHSNIPF SRVNHSKFMV TEKAAYIGTS
beebeeeeee bebebebbbb eeeeeeebeb bebeeeebbb eeebbbbbbb
s                     f   f       f ffff ss ff     sss

451        461        471        481        491
NWSEDYFSST AGVGLVVTQS PGAQPAGATV QEQLRQLFER DWSSRYAVGL
eeeeeebbee bebbbbbeee eeeeeeeeeb eeebeebbee ebeeebbeeb
fff ffs  f  f                      f    s f fs f

501        511
DGQAPGQDCV WQG
eeeeeeeeee eee
       f
```

**The conservation scale:**

| 1 | 2 | 3 | 4 | 5 | 6 | 7 | 8 | 9 |
|---|---|---|---|---|---|---|---|---|

Variable — Average — Conserved

e - An exposed residue according to the neural-network algorithm.

b - A buried residue according to the neural-network algorithm.

f - A predicted functional residue (highly conserved and exposed).

s - A predicted structural residue (highly conserved and buried).

X - Insufficient data - the calculation for this site was performed on less than 10% of the sequences.

# ConSurf Results

```
1           11          21          31          41
MSSGTMKFNG  YLRVRIGEAV  GLQPTRWSLR  HSLFKKGHQL  LDPYLTVSVD
eeeeebebeb  ebebebbebe  ebeeeeeeee  ebeeeeeeee  bbebbbbebe
       s s         fs    s  f    f                 s     f

51          61          71          81          91
QVRVGQTSTK  QKTNKPTYNE  EFCANVTDGG  HLELAVFHET  PLGYDHFVAN
eeeeeeeeee  eeeeeeebee  ebeeebeeee  ebebbbbeeb  ebeeeebbbe
      f       f  f                                  f   s

101         111         121         131         141
CTLQFQELLR  TTGASDTFEG  WVDLEPEGKV  FVVITLTGSF  TEATLQRDRI
bebebeebbe  eeeeeeebeb  bbebeeeeeb  bbbbebeeee  eeeeeeeeee
                           sff f

151         161         171         181         191
FKHFTRKRQR  AMRRRVHQIN  GHKFMATYLR  QPTYCSHCRE  FIWGVFGKQG
eeeeeeeeee  bbeeebeebe  eeebbbebbe  eeebbeebee  bbbbbbeeee
       ff   s ff  f  f  fffs sf     fff sffs    s       f

201         211         221         231         241
YQCQVCTCVV  HKRCHHLIVT  ACTCQNNINK  VDSKIAEQRF  GINIPHKFSI
eebeebebeb  eeebeeebbe  ebeeeeeeee  eeeeeeeeeb  ebebeeebeb
     sf f   fffsf        s                  fs    f ff s

251         261         271         281         291
HNYKVPTFCD  HCGSLLWGIM  RQGLQCKICK  MNVHIRCQAN  VAPNCGVNAV
eebeeeebbb  ebbbbbbbbb  eeebebebbe  eebeeebeee  ebeebebebe
f     fss   fsss s s     ff fs  s    f f fs  f  f   sf f

301         311         321         331         341
ELAKTLAGMG  LQPGNISPTS  KLVSRSTLRR  QGKESSKEGN  GIGVNSSNRL
ebbeebeebe  beeeeeeeee  eeeeeeeeee  eeeeeeeeee  eeeeeeeeee

351         361         371         381         391
GIDNFEFIRV  LGKGSFGKVM  LARVKETGDL  YAVKVLKKDV  ILQDDDVECT
ebeebebbeb  beeeebbebb  bbeeeeeeeb  bbbebbeeeb  bbeeeebebe
        fs  sffffssfs   ss           s f sff    s ffffs sf

401         411         421         431         441
MTEKRILSLA  RNHPFLTQLF  CCFQTPDRLF  FVMEFVNGGD  LMFHIQKSRR
beeeebbebb  eebebbeebb  bbbeeeeebb  bbbbbbbbbe  bbbebeeeee
 ffffss s     sf sf       sff  f    ssss   ssf  ss fsf   f

451         461         471         481         491
FDEARARFYA  AEIISALMFL  HDKGIIYRDL  KLDNVLLDHE  GHCKLADFGM
eeeeebebbb  bebbbbbbbb  eeeebbbeeb  ebeebbbeee  eebebbebeb
f f f  ss   sf   ss  s  f     sffs  fsff  sf    ff f sfsfs

501         511         521         531         541
CKEGICNGVT  TATFCGTPDY  IAPEILQEML  YGPAVDWWAM  GVLLYEMLCG
beeeeeeeee  eeebbeeeee  bbeebbeeee  eebebbbbbb  bbbbbbbbee
sff         f fssfffff  ssffs  f    f  fss ss   sss  ss  f

551         561         571         581         591
HAPFEAENED  DLFEAILNDE  VVYPTWLHED  ATGILKSFMT  KNPTMRLGSL
ebeeeeeeee  ebbebbbeee  bbbbbbbeee  beebbeebbe  eeeeeebbbe
fsffff fff  fss  s  f      s                    f    fss
```

```
601                  611                  621                  631                  641
T Q G G E H A I L R  H P F F K E I D W A  Q L N H R Q I E P P  F R P R I K S R E D  V S N F D P D F I K
e e e e e e b b e e  b b b b e e b e b e  e b e e e e e e e e  b e e e b e e e e e  e e e b e e e b e e
              s      s   s s         s      s             f f      f             f      f s f     s f

651                  661                  671                  681
E E P V L T P I D E  G H L P M I N Q D E  F R N F S Y V S P E  L Q P
e e e e b e e e e e  e b b e e b e e e e  b e e b e b e e e e  e e e
f         f                        f      s     s f                f
```

**The conservation scale:**

| 1 | 2 | 3 | 4 | 5 | 6 | 7 | 8 | 9 |
|---|---|---|---|---|---|---|---|---|
| Variable | | | | Average | | | Conserved | |

e - An exposed residue according to the neural-network algorithm.
b - A buried residue according to the neural-network algorithm.
f - A predicted functional residue (highly conserved and exposed).
s - A predicted structural residue (highly conserved and buried).
X - Insufficient data - the calculation for this site was performed on less than 10% of the sequences.

# ConSurf Results

```
1           11          21          31          41
MDQREILQKF  LDEAQSKKIT  KEEFANEFLK  LKRQSTKYKA  DKTYPTTVAE
eeeeebbeeb  beeeeeeeee  eeebbeebbe  eeeeeeeeee  eeeeeeeeee
ffff        s     f           f     ff ff   f   ff f f  ff

51          61          71          81          91
KPKNIKKNRY  KDILPYDYSR  VELSLITSDE  DSSYINANFI  KGVYGPKAYI
eeeebeeeeb  eebbeeeebe  bebbbbbeee  ebebbebebb  ebbebeebbb
 f f ffffs  ffssf ffs   s ss    ff  fs  sfsf s  fssf f sss

101         111         121         131         141
ATQGPLSTTL  LDFWRMIWEY  SVLIIVMACM  EYEMGKKKCE  RYWAEPGEMQ
beeeeeeebb  bbbbbbbbeb  bbbbbbbbbb  ebeeeeeebe  eebeeeeeee
sfffff  s    sssss sf       ssssss  f ffffffsf  ffs f

151         161         171         181         191
LEFGPFSVSC  EAEKRKSDYI  IRTLKVKFNS  ETRTIYQFHY  KNWPDHDVPS
eebeebbbbb  eeeeeeeebb  bebbebebee  ebeebbbbeb  eebeeeebee
   ffs      f f  f  s   sf sf   f     ff   sfs   fsffff ff

201         211         221         231         241
SIDPILELIW  DVRCYQEDDS  VPICIHCSAG  CGRTGVICAI  DYTWMLLKDG
bbebbbebbb  ebebeeeeee  bebbbbbbee  beeebbbbbb  bbbbebbeee
s fsssf     f f ff       f s sssff  sfffss ss   ss   ss f

251         261         271         281         291
IIPENFSVFS  LIREMRTQRP  SLVQTQEQYE  LVYNAVLELF  KRQMDVIRDK
bbeeebbbbb  bbeebeeeee  ebeeeeeebe  bbbebbbebb  eebbebbeee
s f fss      sffsfffff  f ffffffsf  sss s  fss   f

301         311         321         331         341
HSGTESQAKH  CIPEKNHTLQ  ADSYSPNLPK  STTKAAKMMN  QQRTKMEIKE
eeeeebeeee  eeeeeeeeee  eeeeeeebee  eeeeeeeeee  eeebeeeeee

351         361         371         381         391
SSSFDFRTSE  ISAKEELVLH  PAKSSTSFDF  LELNYSFDKN  ADTTMKWQTK
eeeeebeeee  eeeeeeeeee  ebeeeeebeb  bebebebeee  beeeeeeeee
                              f        f

401         411         421         431         441
AFPIVGEPLQ  KHQSLDLGSL  LFEGCSNSKP  VNAAGRYFNS  KVPITRTKST
bbeeeeeebe  eeeeeebbeb  bbeeeeeeee  eeeeeeeeee  eeebeeeeee
         f  f  f                                      f f

451         461         471         481         491
PFELIQQRET  KEVDSKENFS  YLESQPHDSC  FVEMQAQKVM  HVSSAELNYS
ebebbeeeee  eeeeeeeeee  ebeeeeeeeb  eeeeeeeeee  ebeeeebeee
f f                                               f

501         511         521         531         541
LPYDSKHQIR  NASNVKHHDS  SALGVYSYIP  LVENPYFSSW  PPSGTSSKMS
beeeeeeeee  eeeeeeeeee  eeeeebbbbb  bbeeeebeee  eeeeeeeeeb
                                      fff sf    f

551         561         571         581         591
LDLPEKQDGT  VFPSSLLPTS  STSLFSYYNS  HDSLSLNSPT  NISSLLNQES
eebeeeeeee  ebebeeeeee  eeeebeeeee  eeeeeeeeee  eeeeeeeeeb
```

```
601
A V L A T A P R I D
e e b e e b e e e e

611
D E I P P P L P V R
e e e e e e e e e e
  f   f f f f f   f

621
T P E S F I V V E E
e e e b b b b b e e
f f f s   s s

631
A G E F S P N V P K
e e e b e e e e e e

641
S L S S A V K V K I
e e e e e e e e e e

651
G T S L E W G G T S
e b b e e b e e e e
    s   f

661
E P K K F D D S V I
e e e e e e e e b e

671
L R P S K S V K L R
b e e e e e e e e e
  f           f

681
S P K S E L H Q D R
e e e e e e e e e e

691
S S P P P L P E R
e e e e e e e e e e
      f f f f f f f

701
T L E S F F L A D E
e e e b b b b b e e
f     s       s

711
D C M Q A Q S I E T
e b e e e e e e e e
      f

721
Y S T S Y P D T M E
e e e e e e e e e e

731
N S T S S K Q T L K
e e e e e e e e e e
        f

741
T P G K S F T R S K
e e e e e e e e e e
f     f   f f f f f

751
S L K I L R N M K K
b b e b b e e b e e
s s f s   f f     f

761
S I C N S C P P N K
e b b e e e e e e e
        f

771
P A E S V Q S N N S
e e e e e e e e e e

781
S S F L N F G F A N
e b b b e b b b b e
                  f

791
R F S K P K G P R N
e e e e e e e e e e
f f f f f f f f f

801
P P P T W N I
e e e e e e e
f f     f
```

**The conservation scale:**

| 1 | 2 | 3 | 4 | 5 | 6 | 7 | 8 | 9 |
|---|---|---|---|---|---|---|---|---|
| Variable | | | | Average | | | | Conserved |

e - An exposed residue according to the neural-network algorithm.
b - A buried residue according to the neural-network algorithm.
f - A predicted functional residue (highly conserved and exposed).
s - A predicted structural residue (highly conserved and buried).

# ConSurf Results

```
1                     11                    21                    31                    41
M E G P S L R G P A   L R L A G L P T Q Q   D C N I Q E K I D L   E I R M R E G I W K   L L S L S T Q K D Q
e e e e e e e e e e   e e b e e b e e e e   e e e b e e e b e b   e b b b e e b b b e   b b b e b e e e e e
f                                                       f         f   s   f   s     f   s s   f   f f     f

51                    61                    71                    81                    91
V L H A V K N L M V   C N A R L M A Y T S   E L Q K L E E Q I A   N Q T G R C D V K F   E S K E R T A C K G
b b e b b e e b b b   b e b e b e b b b e   e b e e e e e e e e   e e e e e e e e e e   e e e e e e e b e b
      s   f f     s         f                                                                           s

101                   111                   121                   131                   141
K I A I S D I R I P   L M W K D S D H F S   N K E R S R R Y A I   F C L F K M G A N V   F D T D V V N V D K
e b b b b b b b b b   b b b e e e e e b e   e e e e e e e b b b   b b b b e b e b e b   b e b e b b b b b e
    s   s     s   s   s     f   f     f s       f             s   s s                     f s           s s

151                   161                   171                   181                   191
T I T D I C F E N V   T I F N E A G P D F   Q I K V E V Y S C C   T E E S S I T N T P   K K L A K K L K T S
e b b b b b b e e b   b b b e e b e e e b   e b e b e b b b b b   e e e e e b e e e e   e e b b e e b e e e
f     s s   s s f         s           f s           f     s         f           f f f     f s s   f       f

201                   211                   221                   231                   241
I S K A T G K K I S   S V L Q E E D D E M   C L L L S S A V F G   V K Y N L L A H T T   L T L E S A E D S F
e e e e e e e e b e   e e b e e e e e e e   b b e e e e e e e e   b e b b b b b b b b   b e b e e b e e e b
  f         f                                                 f             s s         s   s             s

251                   261                   271                   281                   291
K T H N L S I N G N   E E S S F W L P L Y   G N M C C R L V A Q   P A C M A E D A F A   G F L N Q Q Q M V E
e e e e b b b b e e   e e b e b b b b b b   b b b b b e b b e e   e e b b b e e b b e   e b b e e e e e b e
f f f   s                   f         s s   s       s s     f f   f   s s               f   s     f

301                   311                   321                   331                   341
G L I S W R R L Y C   V L R G G K L Y C F   Y S P E E I E A K V   E P A L V V P I N K   E T R I R A M D K D
e b b e b e e b b b   b b e e b e b b b b   b e e e e b e e e b   e e e b e b e b e e   e b e b e b b e e e
                        s                         f f     f         f             f       s f

351                   361                   371                   381                   391
A K K R I H N F S V   I N P V P G Q A I T   Q I F A V D N R E D   L Q K W M E A F W Q   H F F D L S Q W K H
e e e e b e e b b b   b b b e e e e e b b   b b b b e e e e e e   b e e b b e b b b e   b b e e e e e b e e
                                                                        s           f   s     f   f f s f f

401                   411                   421                   431                   441
C C E E L M K I E I   M S P R K P P L F L   T K E A T S V Y H D   M S I D S P M K L E   S L T D I I Q K K I
b b e e b b b e b b   e e e e e e e b b b   e e e b e e b b e e   b b b e b e e e b e   b b b e b b e e e b
  s       s   s         f f   f f f           f   s f f s s   f       s   s f   f               s     f

451                   461                   471                   481                   491
E E T N G Q F L I G   Q H E E S L P P P W   A T L F D G N H Q M   V I Q K K V L Y P A   S E P L H D E K G K
e e e e b e b b b e   e e e e e e e e e b   b b b b e e b e e e   b b e e e e e e e e   e e e e e e e e e e
                                                  s   f                                                   f

501                   511                   521                   531                   541
K R Q A P L P P S D   K L P F S L K S Q S   N T D Q L V K D N W   G K T S V S Q T S S   L D T K L S T L M H
e e e e e e e e e e   e e e e e e e e e e   e e e e e e e e e e   e e e e e e e e e e   b e e e b e e e e e
f f f f f   f f

551                   561                   571                   581                   591
H L Q K P M A A P R   K L L P A R R N R L   S D G E H T D T K T   N F E A K P V P A P   R Q K S I K D I L D
e b e e e e e e e e   e e e e e e e e e e   e e e e e e e e e e   e e e e e e e e e e   e e e e b e e e b b
                                                                                        f
```

```
601
  P R S W L Q A Q V
  e e e e b e e e e
  f f           f f
```

**The conservation scale:**

| 1 | 2 | 3 | 4 | 5 | 6 | 7 | 8 | 9 |
|---|---|---|---|---|---|---|---|---|

**Variable** **Average** **Conserved**

**e - An exposed residue according to the neural-network algorithm.**
**b - A buried residue according to the neural-network algorithm.**
**f - A predicted functional residue (highly conserved and exposed).**
**s - A predicted structural residue (highly conserved and buried).**

# ConSurf Results

```
1    MNGPALQPSS
     eeeeeeeeee
        f  f

11   PSSAPSASPA
     eeeeeeeeee

21   AAPRGWSEFC
     eeeeebeebb
          s  ss

31   ELHAVAAARE
     ebbbbbebee
     f ss   s f

41   LARQYWLFAR
     bbeebbebbe
     ss      s

51   EHPQHAPLRA
     eeeeeeebee
       f

61   ELVSLQFTDL
     eebebebbeb
        f  s f

71   FQRYFCREVR
     beebbeeebe
     sf  s  f

81   DGRAPGRDYR
     eeeeeeeeee
            f f

91   DTGRGPPAKA
     eeeeeeeeee

101  EASPEPGPGP
     eeeeeeeeee

111  AAPGLPKARS
     eeeeeeeeee

121  SEELAPPRPP
     eeebeeeeee

131  GPCSFQHFRR
     eebbbeebee

141  SLRHIFRRRS
     bbeebbeeee
       f    f

151  AGELPAAHTA
     eeebeeeeeb
     f

161  AAPGTPGEAA
     beeeeeeeee

171  ETPARPGLAK
     eeeeeeebbe

181  KFLPWSLARE
     ebbeeeeeee
             f

191  PPPEALKEAV
     eeeebeeeeb
           ff

201  LRYSLADEAS
     bebbbbeeeb
           ff

211  MDSGARWQRG
     eeeeeebeeb
      f       s

221  RLALRRAPGP
     ebbbeebeee
     fs  f

231  DGPDRVLELF
     eeeebbbebe
           sfs

241  DPPKSSRPKL
     eeeeeeeeeb
     ffff f ff

251  QAACSSIQEV
     eeebeebeeb
        s    f

261  RWCTRLEMPD
     eebeebebee
     f sf sfsff

271  NLYTFVLKVK
     ebebbbbebe
     f fsss fs

281  DRTDIIFEVG
     eeeebbbebe
        f   f

291  DEQQLNSWMA
     eeeebeebbe
     ffff   s

301  ELSECTGRGL
     ebeebbeeee

311  ESTEAEMHIP
     eeeeeeeeee

321  SALEPSTSSS
     eeeeeeeeee

331  PRGSTDSLNQ
     eeeeeeeeee
        f

341  GASPGGLLDP
     eeeeeeeeee

351  ACQKTDHFLS
     eeeeeeebbb
             s

361  CYPWFHGPIS
     bbebbbbebb
       f sssfss

371  RVKAAQLVQL
     ebebbebbee
     f  ss ssf

381  QGPDAHGVFL
     eeeeeebbbb
      f  ffssss

391  VRQSETRRGE
     beeeeeeeee
     sfffffffff

401  YVLTFNFQGI
     bbbbbebeee
      ssssf ff

411  AKHLRLSLTE
     beebebbbee
     sffsfs  f

421  RGQCRVQHLH
     eeebebeebe
      ffsfsff

431  FPSVVDMLHH
     bbbbbebbbb
     s s   ss s

441  FQRSPIPLEC
     beebebebeb
     sf  fsfsf

451  GAACDVRLSS
     ebbbebebbb
     f    s s

461  YVVVVSQPPG
     bbbbbeeeee

471  SCNTVLFPFS
     eeeebbbbbb

481  LPHWDSESLP
     beebeeebee
         f

491  HWGSELGLPH
     ebeeebebbe
        f

501  LSSSGCPRGL
     beeeebeeee

511  SPEGLPGRSS
     eeeeeeeeee

521  PPEQIFHLVP
     eeeebbebbe

531  SPEELANSLQ
     eeeebeeebe

541  HLEHEPVNRA
     eeeeeeeeee

551  RDSDYEMDSS
     eeeeeebeee
      fffff

561  SRSHLRAIDN
     eeebeebbee
      f   fs ff

571  QYTPL
     eeeee
     fffff
```

**The conservation scale:**

| 1 | 2 | 3 | 4 | 5 | 6 | 7 | 8 | 9 |
|---|---|---|---|---|---|---|---|---|

**Variable** **Average** **Conserved**

**e** - An exposed residue according to the neural-network algorithm.
**b** - A buried residue according to the neural-network algorithm.
**f** - A predicted functional residue (highly conserved and exposed).
**s** - A predicted structural residue (highly conserved and buried).
**X** - Insufficient data - the calculation for this site was performed on less than 10% of the sequences.

# ConSurf Results

```
1                      11                     21                     31                     41
M A E Q V L P Q A L    Y L S N M R K A V K    I R E R T P E D I F    K P T N G I I H H F    K T M H R Y T L E M
e e e e e b e e b b    b b e e b e e b b e    b e e e b e e e b b    e e e e e b b b b b    e b b b e b b b e b
f         s f     s        f f     f s   f    s f           f                        s        s s f   s   f

51                     61                     71                     81                     91
F R T C Q F C P Q F    R E I I H K A L I D    R N I Q A T L E S Q    K K L N W C R E V R    K L V A L K T N G D
b e b b e b b e e b    e e b b e e b b b e    e b b e e e e e e e    e e e e b b e e b e    e b b e b e e e e e
          s       s                      f          f     f f            f f     f f          f s f f f f f

101                    111                    121                    131                    141
G N C L M H A T S Q    Y M W G V Q D T D L    V L R K A L F S T L    K E T D T R N F K F    R W Q L E S L K S Q
e e b b b b b b b e    b b b b b e b b e b    b b e e b b e e b b    e e e e e e e b e b    e b e e e e b e e e
f f   s   s s                s   f s   f s      s f f           s          f f     s        f   f   f       f f

151                    161                    171                    181                    191
E F V E T G L C Y D    T R N W N D E W D N    L I K M A S T D T P    M A R S G L Q Y N S    L E E I H I F V L C
e b e e e b b b b e    e e e b e e e b e e    b b e b b b e e e e    e b e e e b e b e b    b e e b b b b b b b
  s     f   s          f   f s     f s              s   s                                     f f s s s s   s

201                    211                    221                    231                    241
N I L R R P I I V I    S D K M L R S L E S    G S N F A P L K V G    G I Y L P L H W P A    Q E C Y R Y P I V L
b b b e b b b b b b    b e e b b e b b e e    e e e b b e b e b b    b b b b b b e b e e    e e b e e e b e b b
s s s f s s              f       f s     f      f f     f            s   s s s s   s              f f s f     s

251                    261                    271                    281                    291
G Y D S H H F V P L    V T L K D S G P E I    R A V P L V N R D R    G R F E D L K V H F    L T D P E N E M K E
b b e e e e b b b b    b b b e e e e e e b    e b b b b b b e e e    e e b e e b e b b b    b e e e e e e e e e
  s f   f f s   s s            f   f f f      f s   s s                    f       s s    s       f       f

301                    311                    321                    331                    341
K L L K E Y L M V I    E I P V Q G W D H G    T T H L I N A A K L    D E A N L P K E I N    L V D D Y F E L V Q
e b b e e b b e b b    e b b e b b b b e e    e b b b b e b b e b    e e e e b e e e b e    b b e e b b e b b e
  s       s s            s s   f                          s s   s    f f   f s f f     f    s s f f s   f s s f

351                    361                    371                    381                    391
H E Y K K W Q E N S    E Q G R R E G H A Q    N P M E P S V P Q L    S L M D V K C E T P    N C P F F M S V N T
e e b e e b e e e e    e e e e e e e e e e    e e e e e e b e e b    b b b e b e b e e e    e b e b b b b e e e
f f       s f f                                               f s    s         f s f f        s         s f   f

401                    411                    421                    431                    441
Q P L C H E C S E R    R Q K N Q N K L P K    L N S K P G P E G L    P G M A L G A S R G    E A Y E P L A W N P
e e e b e e b b e e    e e e e e e e e e e    e e e e e e e e e e    e e e e e e e e e e    e b e e e e e e e e
f f   s f f s   f

451                    461                    471                    481                    491
E E S T G G P H S A    P P T A P S P F L F    S E T T A M K C R S    P G C P F T L N V Q    H N G F C E R C H N
e e e e e e e e e e    e e e b e b b b b b    b e e e e b e b e e    e e e e b e b e b e    e e e b b e e b e e
            f   f f    f f f   f s            s f f f f s f s f f    f   f f s f   f          f f   s f f s

501                    511                    521                    531                    541
A R Q L H A S H A P    D H T R H L D P G K    C Q A C L Q D V T R    T F N G I C S T C F    K R T T A E A S S S
b e e e e e e e e e    e e e e e e e e e e    b e e b e e e b e e    e b e b b b b e b b    e e e e e e e e e e
                                              s     s   f       f    f s f s   s     s      f f

551                    561                    571                    581                    591
L S T S L P P S C H    Q R S K S D P S R L    V R S P S P H S C H    R A G N D A P A G C    L S Q A A R T P G D
e e e e e e e e e e    e e e e e e e e e b    b e e e e e e e b e    e e e e e e e e e b    e e e e e e e e e e
                       f   f
```

```
601          611          621          631          641
RTGTSKCRKA   GCVYFGTPEN   KGFCTLCFIE   YRENKHFAAA   SGKVSPTASR
eeeeeebeee   ebebeeeeee   eebbbbbbbe   eeeeeeeeee   eeeeeeeeee
      sff        fff       fs s s      f  f                  

651          661          671          681          691
FQNTIPCLGR   ECGTLGSTMF   EGYCQKCFIE   AQNQRFHEAK   RTEEQLRSSQ
beebbebeee   eeeeebeebb   eeebeebbbe   eeeeebeebe   eeeeebeeee
  f   s       f   sf      fffs fs      ff  f                  

701          711          721          731          741
RRDVPRTTQS   TSRPKCARAS   CKNILACRSE   ELCMECQHPN   QRMGPGAHRG
eeeeeeeeee   eeeeebeeee   beeebeeebe   ebbeebeeee   eeeeeeeeee
                            f            s  s                 

751          761          771          781
EPAPEDPPKQ   RCRAPACDHF   GNAKCNGYCN   ECFQFKQMYG
eeeeeeeeee   ebeeebbeee   eeeeeeeebe   ebeebeeeee
        ff    s f  s ff   ff ff ffsf   fs    f ff
```

**The conservation scale:**

| 1 | 2 | 3 | 4 | 5 | 6 | 7 | 8 | 9 |
|---|---|---|---|---|---|---|---|---|

Variable    Average    Conserved

e - An exposed residue according to the neural-network algorithm.
b - A buried residue according to the neural-network algorithm.
f - A predicted functional residue (highly conserved and exposed).
s - A predicted structural residue (highly conserved and buried).
X - Insufficient data - the calculation for this site was performed on less than 10% of the sequences.

# ConSurf Results

```
1                    11                   21                   31                   41
M P L R H W G M A R  G S K P V G D G A Q  P M A A M G G L K V  L L H W A G P G G G  E P W V T F S E S S
e e e b e e e e e e  e e e e e e e e b e  e e e e e e e b e b  b b b b e b e e e e  e e e b e b e e e e
f   f

51                   61                   71                   81                   91
L T A E E V C I H I  A H K V G I T P P C  F N L F A L F D A Q  A Q V W L P P N H I  L E I P R D A S L M
b e b e e b b b e b  b e e b e b b b b b  b b b b b b b e e e  e e b b b e e e e e  b e b e e e b e b e
  f s f              s             s   s      s s   s              s     f f

101                  111                  121                  131                  141
L Y F R I R F Y F R  N W H G M N P R E P  A V Y R C G P P G T  E A S S D Q T A Q G  M Q L L D P A S F E
b b b b b b b b b b  b b e e e e e e e e  e b b e b e e e e e  e e e e e e e e e e  b e b b e e b b b e
          s   s s    s s f f                    f                                     f     f     s s

151                  161                  171                  181                  191
Y L F E Q G K H E F  V N D V A S L W E L  S T E E E I H H F K  N E S L G M A F L H  L C H L A L R H G I
b b b e e e e e e b  b e e b b e b e e e  e e e e e b e e b e  e e b b b b b b b b  b b b b b b e e e e
s       f f f     s                                         f  f f s s s s s s s s  s

201                  211                  221                  231                  241
P L E E V A K K T S  F K D C I P R S F R  R H I R Q H S A L T  R L R L R N V F R R  F L R D F Q P G R L
e b e e b b e e b e  b e e b b e e b b e  e e b e e e e b b b  e b b b e e b b e e  b b e e b e e e e b
                  f          s f     s        s       f     s      s         s      s       s

251                  261                  271                  281                  291
S Q Q M V M V K Y L  A T L E R L A P R F  G T E R V P V C H L  R L L A Q A E G E P  C Y I R D S G V A P
e e e e b e b e b b  b e b e e b b e e b  e e e e b e b b b b  e b e e e e e e e e  e e b e e e e e e e
          f   f s      f s f   s          f   f

301                  311                  321                  331                  341
T D P G P E S A A G  P P T H E V L V T G  T G G I Q W W P V E  E E V N K E E G S S  G S S G R N P Q A S
e e e e e e e e e e  e e e b e b b b b b  e e e b e b e e e e  e e e e e e e e e e  e e e e e e e e e e
                                       s      f s     f

351                  361                  371                  381                  391
L F G K K A K A H K  A V G Q P A D R P R  E P L W A Y F C D F  R D I T H V V L K E  H C V S I H R Q D N
e e e e e e e e e e  e e e e e e e e e e  e e e b e b b b e b  e e b b e b b b e e  b e b e b e e e e e
                                                          f s          f                s         f f

401                  411                  421                  431                  441
K C L E L S L P S R  A A A L S F V S L V  D G Y F R L T A D S  S H Y L C H E V A P  P R L V M S I R D G
e e b e b e b e e e  e e b b b b b b b b  b b b b e b b b e b  e e b b b e e e b e  e e b b e e b e e b
                         s           s    s s s   f s s   f      f   s s     f s f

451                  461                  471                  481                  491
I H G P L L E P F V  Q A K L R P E D G L  Y L I H W S T S H P  Y R L I L T V A Q R  S Q A P D G M Q S L
b e b e b b e e b b  b e e b e e e e b b  b b b e b b b e e b  e e b b b b b b e e  e e e e e e e e e e
  f s f                  f s         s          f   s

501                  511                  521                  531                  541
R L R K F P I E Q Q  D G A F V L E G W G  R S F P S V R E L G  A A L Q G C L L R A  G D D C F S L R R C
e b e e b e b e e e  e e e b e b e e e e  e e e e e b e e b b  e e b e e e e b e e  e e e e b e b e e b
    f f s   s                      f                      s                  s   f

551                  561                  571                  581                  591
C L P Q P G E T S N  L I I M R G A R A S  P R T L N L S Q L S  F H R V D Q K E I T  Q L S H L G Q G T R
e e e e e e e e b e  b b b b e e e e e e  e e e e e b e e b e  b e e b e e e e b e  e e e e e e e e e e
    f             f  s       f                                                            f f f   f f
```

601
T N V Y E G R L R V
e e b b e b e e e e
f f s     s   f

611
E G S G D P E E G K
e e e e e e e e e e

621
M D D E D P L V P G
e e e e e e e e e e

631
R D R G Q E L R V V
e e e e e e b e b b
                s

641
L K V L D P S H H D
b e b b e e e e e e
  f           f

651
I A L A F Y E T A S
b b e b b b e b b e
    f       f   s f

661
L M S Q V S H T H L
b b e e b b e e b b
    f f   s f   s

671
A F V H G V C V R G
b b b b b b b b e e
        s     s

681
P E N I M V T E Y V
e e e b b b e e b b
    f     s f f

691
E H G P L D V W L R
e e b e b e b b b e
    s f   f     s

701
R E R G H V P M A W
e e e e e b e b b b
                  s

711
K M V V A Q Q L A S
e b e b b e e b b e
f     s     f s s

721
A L S Y L E N K N L
b b e e b e e e e e
s s f   s f   f

731
V H G N V C G R N I
e e b e b b b e e b
  f s f s     f f

741
L L A R L G L A E G
b b b e e e e e e e
s     f   f

751
T S P F I K L S D P
e e e b b e b e e e
    f s   f s f f f

761
G V G L G A L S R E
e b b b b b b e e e
f           s

771
E R V E R I P W L A
e b b e e b e e b b
f       f s f f

781
P E C L P G G A N S
e e b b e e e e e e
f f

791
L S T A M D K W G F
b b e b b e e e e b
          f f f

801
G A T L L E I C F D
b b b b b e b b e e
s   s   s f s

811
G E A P L Q S R S P
e e e e e e e e e e
f     f

821
S E K E H F Y Q R Q
e e e e e b e e e e
    f     s f

831
H R L P E P S C P Q
e e e e e e e b e e
    f     f       f

841
L A T L T S Q C L T
b b e b b e e b b e
s s           s

851
Y E P T Q R P S F R
e e e e e e e b b e
f         f f   s f

861
T I L R D L T R L Q
b b b e e b e e b e
      f     f

871
P H N L A D V L T V
e e e e e e b e e e

881
N P D S P A S D P T
e e e e e e e e e e
              f f

891
V F H K R Y L K K I
e b e e e b b e e b
      f f   s

901
R D L G E G H F G K
e e e e e e e b b e
f   f f f f f s s f

911
V S L Y C Y D P T N
b e b b e e e e e e
s f       f f f   f

921
D G T G E M V A V K
e e e e e b b b b e
f   f f f   s s s f

931
A L K A D C G P Q H
e b e e e e e e e e
  s f

941
R S G W K Q E I D I
e e e b e e e b e b
            f s   s

951
L R T L Y H E H I I
b e e b e e e e b b
s     s   f   f s s

961
K Y K G C C E D Q G
e b e b b e e e e e
f s f s   f   f   f

971
E K S L Q L V M E Y
e e b b e b b b e b
          s   s f

981
V P L G S L R D Y L
b b e e e b e e e b
  s   f f s f

991
P R H S I G L A Q L
e e e e e e b e e b

1001
L L F A Q Q I C E G
b e e b b e b b e b
    f     f s s   s

1011
M A Y L H A Q H Y I
e e b b b b e e b b
f   s s       f

1021
H R D L A A R N V L
b e b b b b e e b b
s f s s s s f f   s

1031
L D N D R L V K I G
b e e e e e b e b b
s f   f     s f s s

1041
D F G L A K A V P E
e b e b b e e e e e
f s f s   f   f f

1051
G H E Y Y R V R E D
e e e e b e b e e e
      f s   s   f

1061
G D S P V F W Y A P
e e e e b b b b b b
  f f f s   s   s

1071
E C L K E Y K F Y Y
e b b e e b e b e b
f s s         f s f

1081
A S D V W S F G V T
b e b b b b b b b b
  f s   s s s s s s

1091
L Y E L L T H C D S
b b e b b e e b e e
  s f     f

1101
S Q S P P T K F L E
e e e e e e e b b e
    f f f

1111
L I G I A Q G Q M T
b b e e e e e e b b

1121
V L R L T E L L E R
b b e b b e b b e e
      s       s

1131
G E R L P R P D K C
e e e e e e e e e e
    f f f   f     f

1141
P C E V Y H L M K N
e e e b e e b b e e
f              s

1151
C W E T E A S F R P
b e e e e e e e e e
  f             f f

1161
T F E N L I P I L K
e b e e b b e e b e
  s     s

1171
T V H E K Y Q G Q A
e b e e e e e e e e

1181
P S V F S V C
e e e b e e e
      s

**The conservation scale:**

| 1 | 2 | 3 | 4 | 5 | 6 | 7 | 8 | 9 |
|---|---|---|---|---|---|---|---|---|

**Variable** **Average** **Conserved**

- **e** - An exposed residue according to the neural-network algorithm.
- **b** - A buried residue according to the neural-network algorithm.
- **f** - A predicted functional residue (highly conserved and exposed).
- **s** - A predicted structural residue (highly conserved and buried).
- **X** - Insufficient data - the calculation for this site was performed on less than 10% of the sequences.

# ConSurf Results

```
1                     11                    21                    31                    41
M E A E D L S K A E   D R N E D P G S K N   E G Q L A A V Q P D   V P H G G Q S S S P   T A L W D M L E R K
e e e e e e e e e e   e e e e e e e e e e   e e e e e e e e e e   e e e e e e e e e e   e b b b e e b e e e
f f

51                    61                    71                    81                    91
F L E Y Q Q L T H K   S P I E R Q K S L L   S L L P L F L K A W   E H S V G I I C F P   S L Q R L A E D V S
b e e e e e e e e e   e e e e e e e e b e   e b b e b b b e b b   e e e b e b b b b e   e b e e b b e e b b
                                                s                     f                               s

101                   111                   121                   131                   141
D Q L A Q Q L Q K A   L V G K P A E Q A R   L A A G Q L L W W K   G D V D Q D G Y L L   L K S V Y V L T G T
e e b b e e b e e e   b e e e e e e e b e   e e b e e b b e e e   e e e e e e b b b b   b e b b b b b b e e
                                f f   s f                                           s   s f

151                   161                   171                   181                   191
D S E T L G R V A E   S G L P A L L L Q C   L Y L F F V F P L D   K D E L L E S D L Q   V Q K M F V Q M L L
e e e b b b e b b e   e e b e e b b b e b   b e b b b b b e e e   e e e e e e e e e e   b e e b b b e b b b
                          s f         f s   s f s s s     f                               f

201                   211                   221                   231                   241
N I C S D S Q G L E   G L L S G S E L Q S   L L I A T T C L R E   H S C C F W K E P T   F C V L R A I S K A
e b b e e e e e b e   e b b e e e e b e b   b b b b b b b b e e   e b e e e b e e e b   b e b e b b b e e e
                  f               f               s           f             s f               s       f

251                   261                   271                   281                   291
Q N L S I I Q Y L Q   A T D C V R L S L Q   N L S R L T D T L P   A P E V S E A V S L   I L G F V K D S Y P
e e e e b b e b b e   b e e b b e b b b e   e b e e b e e e b e   e e e b b e b b b b   b b b b b e e e e e
                                      f   f                               f                   s   f f f f

301                   311                   321                   331                   341
V S S A L F L E F E   N S E G Y P L L L K   V L L R Y D G L T Q   S E V D P H L E E L   L G L V V W L T T C
b b e b b b b e b e   e e e e b e b b b e   b b e e b e e e e e   e e e e e e b e e b   b e b b b b b b e b
        s     f s f         f s                                                   f                   s

351                   361                   371                   381                   391
G R S E L K V F D S   I T Y P Q L E G F K   F H H E A S G V T V   K N L Q A F Q V L Q   N V F H K A S D S V
e e e e b e b b e e   b e b e e e e e b e   b e e e e e e b e b   e e b e b b e b b e   e b b e e e e e e e
f     f s f s     f         f f   f   s                 f         f f     s     s   f       s

401                   411                   421                   431                   441
L C I Q V L S V I R   T M W A W N A R N F   F L L E W T L Q P I   S Q F V E I M P L K   P A P V Q E H F F Q
b b b e b b e b b e   e b b e e e e e b b   b b b e b b b e e b   e e b b e b b e e e   e e e b e e b b b e
                                      s s   s   s f s     f         f                 f         s f

451                   461                   471                   481                   491
L L E A L V F E L H   Y V P H E I L R K V   Q H L I K E S P G P   S C T L M A L Q S I   L S I A G G D P L F
b b e b b b b e b e   b b e e e b b e e b   e e b b e e e e e e   e b b b b b b e b b   b e b b e e e b b b
    f                     f f                                                                     f

501                   511                   521                   531                   541
T D I F R D S G L L   G L L L A Q L R K Q   A K I M R K S G N K   V S T P G V Q D P E   R E L T C V M L R I
b e b b e e b e b b   e b b b e e b e e e   b e b b e e e e e e   e e e e e e e e e e   e e b b b b b b e b
  f s   f f   f           s s     s f       s f     f f                                   s       s s

551                   561                   571                   581                   591
V V T L L K G S V R   N A V V L K D H G M   V P F I K I F L D D   E C Y R E A S L S I   L E Q L S A I N A E
b b b b b e e e b e   e b b b b e e e e b   b b b b e b b b e e   e e e e e b b b e b   b e e b b e b e b e
s             f   f   f         f f   f s     s     f s     f         f       s   s     f f         f   f
```

601
E Y M S I I V G A L
e b b b b b b b b b
f   s s       s s

611
C S S T Q G E L Q L
b e e e e e e b e b
s f f f f   f

621
K L D L L K S L L R
e e e b b e e b b e
f   f   s   f

631
I L V T P K G R A A
b b e e e e e e e b
  s               s

641
F R V S S G F N G L
b e e b e b b e b b
s f     f s s

651
L S L L S D L E G S
b b b b b e b e e e
  s       f s f f f

661
L Q E P P L Q A W G
b e e e e e e b b e
s

671
A V S P R Q T L E L
e e e e e e b b e b

681
V L Y T L C A V S A
b b e b b b b b b b

691
A L H W D P V N G Y
b b e b b e b e e e
s       s     f

701
F F R R N G L F E K
b b e e e e b b e e
  s             f

711
L A E D L C L L G C
b b e e b b e b b b
      f         s s

721
F G A L E E E G N L
b e e e e e e e e e
s

731
L R S W V D T K A R
e e e e e e e e e e

741
P F A D L L G T A F
e b e e b b e b b e

751
S S S G S L P P R I
e e e e e b e e e b
            f

761
Q S C L Q I L G F L
e e b b e b b e b b
    s             s

771
D S M A S G T L H L
e e b b e b e b e b

781
R G D L K E S L R T
e e e b e e e e e e

791
K Q G P V V D V Q K
e e e e e e e e e e

801
G E T G S D P Q R N
e e e e e e e e e e

811
F K Q W P D L E E R
e e e e e e e e e e

821
M D E G D A A I M H
e e e e e b b b b b
              s

831
P G V V C I M V R L
b b b b b b b b e b
s                 s

841
L P R L Y H E D H P
b e e b e e e e e e
  f

851
Q L S E E I Q C S L
e b e e e b e b b b
    f   f   f

861
A S H I Q S L V K S
b e b b e e b b e e
        f     s f

871
E K N R Q V M C E A
e e e e e b b b e e
f   f f f     s

881
G L L G T L M A S C
e b b e b b b e e b
f

891
H R A L V T S G S P
e e b b e e e e e e

901
L H S R L I R I F E
b e b e b b e b b e
  f         f     f

911
K L A S Q A I E P D
e b b e e e b e e e
f s   f f   s

921
V L R Q F L G L G I
e b e e b b e b e b
    f

931
P S S L S A T T K I
e e e e e e e e e e

941
L D S S H T H R G N
e e e e e e e e e e

951
P G C S G S Q T A Q
e e e e e e e e e e

961
G L A E G P W P A A
e e e e e e e e e e

971
P D A G L H P G V T
e e e e e e e e b b

981
Q A P Q P L G E S Q
e e e e e e e e e e

991
D S T T A L Q T A L
e e e e b b e b b b
            f

1001
S L I S M T S P R N
b b b b b b b e e e
  s   s   s s f f

1011
L Q P Q R A A L A P
b e e e e e e e b e
                  f

1021
S F V E F D M S V E
b b b e b e b e e e
  s   f s f s     f

1031
G Y G C L F I P T L
b b b b b b b b b b
s   s   s     s

1041
S T V M G T S T E Y
b e b e e e b e e e
  f     f       f

1051
S V S G G I G T G A
e b b b b b b b b e
    f s s   s

1061
T R P F P P P G G L
e e e e e e e e e b
  f   f f f     f

1071
T F S C W F L I S R
b b b b b b b b b e
    s   s

1081
H G A A T E G H P L
b b e b b e e b e b
              s f

1091
R F L T L V R H L A
e b b b b b e e b e
    s     s f f

1101
R T E Q P F V C F S
e e e e e b b b b b
f f f           s

1111
V S L C P D D L S L
b b b b e e e b b b
            f     s

1121
V V S T E E K E F Q
b b b e e e e e b e
    s f f f f     f

1131
P L D V M E P E D D
e b e b e e e e e e
    f   f f f f

1141
S E P S A G C Q L Q
e e e e e e e e b e

1151
V R C G Q L L A C G
b e b e e b b b e b

1161
Q W H H L A V V V T
e b e b b b b b b b
f s f s s

1171
K E M K R H C T V S
e e b e e e b e b b
f f   f

1181
T C L D G Q V I G S
b b b e b e b e e b
      f         f

1191
A K M L Y I Q A L P
b e b e b b e e b e
  f     s   f     f

```
1201        1211        1221        1231        1241
GPFLSMDPSA  FVDVYGYIAT  PRVWKQKSSL  IWRLGPTYLF  EEAISMETLE
eebbbbeeeb  bbbbbbbbbb  eebbeeebeb  bbebbeebbb  eebbeeebbe
f    s f      s      s  f      s s   sf sf      ff  f

1251        1261        1271        1281        1291
VINKLGPRYC  GNFQAVHVQG  EDLDSEATPF  VAEERVSFGL  HIASSSITSV
bbbeebeeeb  eebebbeeee  eeeeeebbeb  bbeeebebbb  bbbbbebbbb
    fsf f   ff                        ff   fs

1301        1311        1321        1331        1341
ADIRNAYNEV  DSRLIAKEMN  ISSRDNAMPV  FLLRNCAGHL  SGSLRTIGAV
bebeeebeeb  ebebbbeebe  bebeeebbbb  bbbeebeeeb  eeebeebbbb
   f   ffs  f f ssff      sff   s       f f fs   f  ff ss

1351        1361        1371        1381        1391
AVGQLGVRVF  HSSPAASSLD  FIGGPAILLG  LISLATDDHT  MYAAVKVLHS
bbbbbbbebb  eeeeebebbe  bbbbebbbbb  bbbbbeeeee  bbbbbebbbe
  s  s f s   f  f    f    ssf   s    s s ff     ss  f   f

1401        1411        1421        1431        1441
VLTSNAMCDF  LMQHICGYQI  MAFLLRKKAS  LLNHRIFQLI  LSVAGTVELG
bbeeeebbee  ebeebebbeb  bbbbbeeeee  ebebebbebb  bbbbbbbebe
 s ff       fs f  ss       ss  f      f f  fs

1451        1461        1471        1481        1491
FRSSAITNTG  VFQHILCNFE  LWMNTADNLE  LSLFSHLLEI  LQSPREGPRN
beeeebeebe  bbebbbbebe  bbeebeeebe  bbbbeebbeb  beeeeeeeee
       f             f   s      s        f        f f    f

1501        1511        1521        1531        1541
AEAAHQAQLI  PKLIFLFNEP  SLIPSKISTI  IGILACQLRG  HFSTQDLLRI
bebbeebebb  eebbbbbeee  eeeeeebebb  bbbbbbbbee  ebebeebbeb
                                                     f  f

1551        1561        1571        1581        1591
GLFVVYTLKP  SSVNERQICM  DGALDPSLPA  GSQTSGKTIW  LRNQLLEMLL
bbbbbbbbee  eeeeeeeebb  eeeeeeeeee  eeeeeeebbb  beeebbebbb
  s   s      f  f                       f       sfff s

1601        1611        1621        1631        1641
SVISSPQLHL  SSESKEEMFL  KLGPDWFLLL  LQGHLHASTT  VLALKLLLYF
ebbeeeeeeb  eeeeeeebbe  ebbeebbbbb  beeeeeebbb  ebbbebbbbb
    f                    ss fs s     f   f  s

1651        1661        1671        1681        1691
LASPSLRTRF  RDGLCAGSWV  ERSTEGVDIV  MDNLKSQSPL  PEQSPCLLPG
beeeebeeeb  eeebbebebb  eebeeebebb  beebeeeeee  eeeeebebeb
s        s                            f                  s

1701        1711        1721        1731        1741
FRVLNDFLAH  HVHIPEVYLI  VSTFFLQTPL  TELMDGPKDS  LDAMLQWLLQ
bebbeebbee  bbebbebbbb  bbbbbbeeee  eeeeeeeeee  beebbeebbe
   s   s

1751        1761        1771        1781        1791
RHHQEEVLQA  GLCTEGALLL  LEMLKATMSQ  PLAGSEDGAW  AQTFPASVLQ
eeeeeeeeee  ebbeebbbbb  bebbebbbee  eeeeeeeeeb  eeebeeebbe
                f
```

```
1801
FLSLVHRTYP
bbbbbbeebe
s

1811
QDPAWRAPEF
eeeeeebeeb
 f  f

1821
LQTLAIAAFP
bebbbebbbe
s  s     f

1831
LGAQKGVGAE
beeeeeeeee

1841
STRNTSSPEA
eeeeeeeeeb

1851
AAEGDSTVEG
eeeeeeeeee

1861
LQAPTKAHPA
eeeeeeeeee

1871
RRKLREFTQL
eeebeebbeb
   s

1881
LLRELLLGAS
bbeebbeebe
   f s

1891
SPKQWLPLEV
eeeeeeebeb

1901
LLEASPDHAT
bbebbeeeee
  f

1911
SQQKRDFQSE
eeeeeebeee
  f    ff

1921
VLLSAMELFH
bbebbbbbbb

1931
MTSGGDAAMF
bbeeeeeeee

1941
RDGKEPQPSA
eeeeeeeeee

1951
EAAAAPSLAN
eeebbebbee
         f

1961
ISCFTQKLVE
bbbbbeebbe

1971
KLYSGMFSAD
ebeeebbeee

1981
PRHILLFILE
eeebbbbbbe
      s  f

1991
HIMVVIETAS
bbbbbbeeee
 s

2001
SQRDTVLSTL
eeeeebbebb

2011
YSSLNKVILY
bebbeebbbb
s   f   s

2021
CLSKPQQSLS
bbeeeeeeee
 sf f

2031
ECLGLLSILG
eeeebbebbe

2041
FLQEHWDVVF
bbeeebebbb
      f

2051
ATYNSNISFL
bbbeeebebb
s  f f  s

2061
LCLMHCLLLL
bbbbbbbbbb
 s   s

2071
NERSYPEGFG
eeeeeeeeee
   f   f

2081
LEPKPRMSTY
eeeeeeeeee

2091
HQVFLSPNED
ebbbbbeeee

2101
VKEKREDLPS
eeeeeeeeee

2111
LSDVQHNIQK
beeeeeebee

2121
TVQTLWQQLV
bbeebbeebb
     s

2131
AQRQQTLEDA
eeeeeebeeb
  f   sff

2141
FKIDLSVKPG
bebebeeeee
 f f f

2151
EREVKIEEVT
eeebebeebe
         f

2161
PLWEETMLKA
ebbeebbeeb
f  ff   f

2171
WQHYLASEKK
beebbeeeee
s     ff

2181
SLASRSNVAH
eeeeeeeeee

2191
HSKVTLWSGS
eeeeeebbeb
 ff

2201
LSSAMKLMPG
bbebbebbee
  f

2211
RQAKDPECKT
eeeeeeeeeb

2221
EDFVSCIENY
eebbebbeeb

2231
RRRGQELYAS
eeebeebbee

2241
LYKDHVQRRK
beeeebeeee
      f

2251
CGNIKAANAW
beeeebbeeb
         s

2261
ARIQEQLFGE
bebeeebbee
   f  s  f

2271
LGLWSQGEET
eebbbeeeee
ff

2281
KPCSPWELDW
eeeeebeeeb
       f

2291
REGPARMRKR
eeeeeebeee
 fff fs  f

2301
IKRLSPLEAL
beeeeeeeee
 f

2311
SSGRHKESQD
eeeeeeeeee

2321
KNDHISQTNA
eeeeeeeeee

2331
ENQDELTLRE
eeeeeeeeee

2341
AEGEPDEVGV
eeeeeeeeee

2351
DCTQLTFFPA
ebeebebbee
     f  f

2361
LHESLHSEDF
beeeeeeeeb

2371
LELCRERQVI
eeebeeeebb

2381
LQELLDKEKV
beebeeeeeb
 ff    f

2391
TQKFSLVIVQ
eeebbbbbbb
        s
```

```
2401
G H L V S E G V L L
b b b b b e b b b b
          f     s s

2411
F G H Q H F Y I C E
b b e e b b b b b e
                  f

2421
N F T L S P T G D V
e b e b e e e e e b
    f   f     f f

2431
Y C T R H C L S N I
e b e e e e e e e b
        f     f

2441
S D P F I F N L C S
e e e b b b b b b e
  f

2451
K D R S T D H Y S C
e e e e e e e e e b
                  s

2461
Q C H S Y A D M R E
e e e e e e e b e e
        f   f

2471
L R Q A R F L L Q D
b e e b e b b b e e
        f   s   f

2481
I A L E I F F H N G
b b b e b b b e e e
  s   f         f f

2491
Y S K F L V F Y N N
e b e b b b b e e e
  s     s s     f

2501
D R S K A F K S F C
e e e e b b e e b b
f f         f

2511
S F Q P S L K G K A
e b e e e b e e e e
            f

2521
T S E D T L S L R R
b e e e b e e b e e
    f

2531
Y P G S D R I M L Q
e e e e e e e b b e

2541
K W Q K R D I S N F
e b e e e e b e e b
  s f   f     f f s

2551
E Y L M Y L N T A A
e b b b b b b b b b
f s s s   s s s   s

2561
G R T C N D Y M Q Y
e e b b e e b b e b
f f s   f f   s f s

2571
P V F P W V L A D Y
e b b b b b b b e b
f s   s s     s f s

2581
T S E T L N L A N P
e e e e b e b e e e
  f       f

2591
K I F R D L S K P M
e e b e e b b e e e
  f s f f s s f f f

2601
G A Q T K E R K L K
e e e e e e e e e e
f f f f   f f     f

2611
F I Q R F K E V E K
b b e e b e e e e e
s     f       f

2621
T E G D M T V Q C H
e e e e b e b e b b
    f           s s

2631
Y Y T H Y S S A I I
b b e b b b b b b b
    f s s s s s s s

2641
V A S Y L V R M P P
b b b b b b e b e e
    s   s s f   f f

2651
F T Q A F C A L Q G
b e e b b b e b e e
s f f         s f f

2661
G S F D V A D R M F
e e b e b b e e b b
f f   f     f f s s

2671
H S V K S T W E S A
e b b e e e b e e b
  s         s   f s

2681
S R E N M S D V R E
b e e e b e e b e e
s   f f s f f s f f

2691
L T P E F F Y L P E
b b e e b b e b b e
s   f f s   f     f

2701
F L T N C N G V E F
b b e e b e e b e b
s     f   f

2711
G C M Q D G T V L G
b b e e e e e e b e
s   f f f f f     f

2721
D V Q L P P W A D G
e b e b b e e e e e
f s   s s f f f f

2731
D P R K F I S L H R
e e e e b b e b b e
f     f s   f   s f

2741
K A L E S D F V S A
e b b e e e b b b b
f s s f f f   s s

2751
N L H H W I D L I F
b b e b b b b b b b
  s f   s s s s s s

2761
G Y K Q Q G P A A V
b b e e e e e e b b
s     f   f   f s

2771
D A V N I F H P Y F
e b b b e b b b b b
      s f   s s s s

2781
Y G D R M D L S S I
b b e e e e e e e b
s

2791
T D P L I K S T I L
e e e b b e b b b b
  f f         s

2801
G F V S N F G Q V P
b b b b e b e e e e
s       f s f f f f

2811
K Q L F T K P H P A
e e e e e e e e e e
f f   f   f f f f

2821
R T A A G K P L P G
e e e e e e e e e e
f

2831
K D V S T P V S L P
e e e e e e e e e e

2841
G H P Q P F F Y S L
e e e e e b b e e b

2851
Q S L R P S Q V T V
e e b e e e e e e b
              f

2861
K D M Y L F S L G S
e e b b b b b b e e
f

2871
E S P K G A I G H I
e e e e e e e b b b
f       f   f s   s

2881
V S T E K T I L A V
b b e e e e b b b b
        f

2891
E R N K V L L P P L
e e e e e b b b e b
f   f

2901
W N R T F S W G F D
b e e b b b b b b e
            s s

2911
D F S C C L G S Y G
e e e b b e e e e e
f   f

2921
S D K V L M T F E N
e e e b b b b b e b
f   f           f

2931
L A A W G R C L C A
b b e b b e b b b e
        s         f

2941
V C P S P T T I V T
e b e e e e b b b b
    f             s

2951
S G T S T V V C V W
b b e b b b b b b b
  s   s   s s s s s

2961
E L S M T K G R P R
e b b e e e e e e e
          f

2971
G L R L R Q A L Y G
e b e b e e e b e e
  s           s f f

2981
H T Q A V T C L A A
e e e b b b b b b b
f f     s s s

2991
S V T F S L L V S G
b e b b b b b b e b
s               f s
```

```
3001                 3011                 3021                 3031                 3041
S Q D C T C I L W D  L D H L T H V T R L  P A H R E G I S A I  T I S D V S G T I V  S C A G A H L S L W
e e e e b b b b b e  b e e b e e b b e b  e e e e e b b b b b  b b b e b e e e b b  b b b b b b b b b b
f   f   s s s   s f    f               s      f                    s     f f        s s               s

3051                 3061                 3071                 3081                 3091
N V N G Q P L A S I  T T A W G P E G A I  T C C C L M E G P A  W D T S Q I I I T G  S Q D G M V R V W K
e b e e e e b e b b  e e e e e e e b e b  b b b b b b e e e e  e e e e e b b b b b  b e e b b b e b b e
    f f                                               f          f     f       s s      f s     f   s

3101                 3111                 3121                 3131                 3141
T E D V K M S V P G  R P A G E E P P A Q  P P S P R G H K W E  K N L A L S R E L D  V S I A L T G K P S
e e e e e e e e e e  e e e e e e e e e e  e e e e e e e e b e  e b b b b b e e b e  e e b e b e e e e e
f f                                                            f             f s              f

3151                 3161                 3171                 3181
K T S P A V T A L A  V S R N H T K L L V  G D E R G R I F C W  S A D G
e e e e b b b b b b  b b e e e b e b b b  b e e e b e b b b b  e e e e
            s s          f f f   f     s  s f     s f       s        f
```

## The conservation scale:

| 1 | 2 | 3 | 4 | 5 | 6 | 7 | 8 | 9 |
|---|---|---|---|---|---|---|---|---|

Variable — Average — Conserved

e - An exposed residue according to the neural-network algorithm.
b - A buried residue according to the neural-network algorithm.
f - A predicted functional residue (highly conserved and exposed).
s - A predicted structural residue (highly conserved and buried).
X - Insufficient data - the calculation for this site was performed on less than 10% of the sequences.

# ConSurf Results

```
1
M S R P R M R L V V
e e e e e b e b b b
f             s

11
T A D D F G Y C P R
b b e e b e e e e e
  s f f   f f f

21
R D E G I V E A F L
e e e e b b e b b b
f f   f s

31
A G A V T S V S L L
e e e b b e b b b b
          f   s

41
V N G A A T E S A A
b e b e e b e e b b
  f             s

51
E L A R R H S I P T
e b b e e e e b e b

61
G L H A N L S E G R
b b b b e b e e e e
s   s   f s f f f

71
P V G P A R R G A S
e e b e e e e e e e
f

81
S L L G P E G F F L
e b e e e e b b b e

91
G K M G F R E A V A
b e e e b e e b b e
  f

101
A G D V D L P Q V R
e e e b e e e e b e
              f

111
E E L E A Q L S C F
e e b e e e b e e b
  f     f f

121
R E L L G R A P T H
e e b e e e e b e e
        f         f

131
A D G H Q H V H V L
b e e e e b b b b b
  f f f f s   s

141
P G V C Q V F A E A
e e b b e b b b e b
f

151
L Q A Y G V R F T R
b e e b e b e b b e
                  f

161
L P L E R G V G G C
b e e e e e b e e b
  f   f

171
T W L E A P A R A F
e b b e e e b e e b

181
A C A V E R D A R A
b e e b e e e b e e

191
A V G P F S R H G L
b e e b b e e e b b

201
R W T D A F V G L S
e b e e b b b b b b
              s

211
T C G R H M S A H R
b e b e e b e e e e
  f

221
V S G A L A R V L E
b e e b b e e b b e

231
G T L A G H T L T A
e e e e e e e b b b

241
E L M A H P G Y P S
e b b b b e e e e e
f   s   s f f

251
V P P T G G C G E G
e e e e e e e e e e
          f f f   f

261
P D A F S C S W E R
e e e b e e e e e e
f f   s f   f     f

271
L H E L R V L T A P
e e e b e e b e e e
    f       s

281
T L R A Q L A Q D G
e b e e e b e e e e

291
V Q L C A L D D L D
b e b b b b e e e e
                f

301
S K R P G E E V P C
e e e e e e e e e e

311
E P T L E P F L E P
e b e b e e b e e e

321
S L L
e e e
f
```

**The conservation scale:**

| 1 | 2 | 3 | 4 | 5 | 6 | 7 | 8 | 9 |
|---|---|---|---|---|---|---|---|---|

**Variable**    **Average**    **Conserved**

e - An exposed residue according to the neural-network algorithm.
b - A buried residue according to the neural-network algorithm.
f - A predicted functional residue (highly conserved and exposed).
s - A predicted structural residue (highly conserved and buried).
X - Insufficient data - the calculation for this site was performed on less than 10% of the sequences.
