# Supplementary material for: Characterization of Rheumatoid Arthritis Risk-Associated SNPs and Identification of Novel Therapeutic Sites Using an In-Silico Approach
Source: Biology (Basel). 2021 Jun 4;10(6):501. doi: 10.3390/biology10060501 (PMC8227790; doi:10.3390/biology10060501)
Supplement: Supplementary file 1 [file biology-10-00501-s001.zip › Supplementary files_2021.pdf]

Characterization of rheumatoid arthritis risk-associated SNPs and identification of novel therapeutic sites using an *in-silico* approach

Mehran Akhtar[1,2#], Yasir Ali[1#], Zia-ul Islam[1], Maria Arshad[3], Mamoona Rauf[4], Muhammad Ali[2], Suleman Khan[5], Saleh N. Maodaa[6], Saleh A. Al-Farraj[6], Hamed A El-Serehy[6], Fazal Jalil[*1]

[1]Department of Biotechnology, Abdul Wali Khan University, Mardan, Pakistan

[2]Department of Biotechnology, COMSATS University Islamabad, Abbottabad Campus, Abbottabad, Pakistan

[3]Attaur Rahman School of Applied Biosciences, NUST, Islamabad, Pakistan

[4]Department of Botany, Abdul Wali Khan University Mardan, Pakistan

[5]Consultant Rheumatologist, Lady Ready Hospital-MTI Peshawar, Pakistan

[6]Department of Zoology, College of Science, King Saud University, Riyadh I1451, Saudi Arabia

*Corresponding Author: Dr. Fazal Jalil, Department of Biotechnology, Abdul Wali Khan University, Mardan, Pakistan. Email: fazaljalil@awkum.edu.pk

[#]Mehran Akhtar and Yasir Ali are shared first author.

S1 Table: List of SNPs associated with Rheumatoid Arthritis

S.No	Gene	SNP ID	Chromosome Position	Nucleotide Change	Type	Amino acid change	Global MAF
	PTPN22	rs33996649	chr1:113852067	NC_000001.11:g.113852067C>T	missense	R263Q	T=0.0110
		rs3765598	chr1:113851841	NC_000001.11:g.113851841C>T	Intronic		T=0.1336
		rs3811021	chr1:113814041	NC_000001.11:g.113814041A>G	3'UTR		G=0.1334
		rs1217414	chr1:113870045	NC_000001.11:g.113870045G>A	Intronic		A=0.3329
		rs2476601	chr1:113834946	NC_000001.11:g.113834946A>G	missense	R620W	A=0.0274
	PADI4	rs11203366	chr1:17331039	NM_012387.2:c.163G>A	missense	G55S	G=0.4754
		rs11203367	chr1:17331121	NM_012387.2:c.245T>C	misense	V82A	T=0.4667
	FCRL3	rs7528684	chr1:157701026	NG_023241.1:g.4832T>C	Near 5'Gene		A=0.4493
		rs11264799	chr1:157700967	NG_023241.1:g.4891G>A	Near 5'Gene		T=0.2712
		rs945635	chr1:157700500	NG_023241.1:g.5358G>C	5'UTR		C=0.4567
		rs3761959	chr1:157699488	NG_023241.1:g.6370G>T	Intronic		T=0.4623
	CTLA4	rs231779	chr2:203869764	NG_011502.1:g.6979C>T	Intronic		T=0.4403
		rs231777	chr2:203868865	NG_011502.1:g.6080T>C	Intronic		T=0.1352
		rs231775	chr2:203867991	NG_011502.1:g.5206A>G	missense	T17A	G=0.4273
		rs5742909	chr2:203867624	NG_011502.1:g.4839C>T	Near 5'Gene		T=0.0517
	TRAF1/C5	rs10818488	chr9:120942809	NC_000009.12:g.120942809A>G	3'UTR		A=0.4742
		rs3761847	chr9:120927961	NG_023346.1:g.6213C>T	Intonic		G=0.4609
		rs10760130	chr9:120939712	NC_000009.12:g.120939712G>A			A=0.4521
		rs2900180	chr9:120944104	NC_000009.12:g.120944104T>A	Intronic		T=0.3043
	TNFAIP3	rs5029941	chr6:137874923	NG_032761.1:g.12480C>T	missense	A125V	T=0.0060
		rs2230926	chr6:137874929	NG_032761.1:g.12486T>G	missense	F127S	G=0.1396
		rs5029930	chr6:137869547	NG_032761.1:g.7104A>C	Intronic		C=0.2059
		rs5029937	chr6:137874014	NG_032761.1:g.11571G>T	Intronic		T=0.1396
		rs5029939	chr6:137874586	NG_032761.1:g.12143C>G	Intronic		G=0.1396
		rs13207033	chr6:137644281	NC_000006.12:g.137644281G>A			A=0.2053
		rs10499194	chr6:137681500	NC_000006.12:g.137681500C>T			T=0.1915
		rs6920220	chr6:137685367	NC_000006.12:g.137685367G>A			A=0.0944
	STAT4	rs7574865	chr2:191099907	NG_012852.1:g.56293A>C	Intronic		T=0.2554
	IL2-IL21	rs6822844	chr4:122588266	NC_000004.12:g.122588266G>T			T=0.0557
		rs17388568	chr4:122408207	NC_000004.12:g.122408207G>A	Intronic		A=0.1406

		rs907715	chr4:122613898	NG_031966.1:g.12160G>T	Intronic		T=0.3660
	IL2RB	rs743777	chr22:37155567	NC_000022.11:g.37155567A>G	Intronic		G=0.2768
		rs3218253	chr22:37148770	NC_000022.11:g.37148770G>A	Intronic		A=0.1472
	IL2RA	rs2104286	chr10:6057082	NG_007403.1:g.10228A>G	Intronic		C=0.1306
	CD40	rs1883832	chr20:46118343	NG_007279.1:g.5077T>C	5'UTR		T=0.2288
		rs4810485	chr20:46119308	NG_007279.1:g.6042T>A	Intronic		T=0.2384
		rs1535045	chr20:46119460	NG_007279.1:g.6194C>T	Intronic		T=0.1961
		rs3765459	chr20:46128768	NG_007279.1:g.15502G>A	Intronic		A=0.1875
	CCL21	rs2812378	chr9:34710263	NC_000009.12:g.34710263G>A	Near 5' Gene		G=0.3013
		rs13293020	chr9:34768696	NC_000009.12:g.34768696C>T			T=0.0903
	AFF3	rs10865035	chr2:100219272	NC_000002.12:g.100219272A>G	Intronic		G=0.3932
		rs1160542	chr2:100215693	NC_000002.12:g.100215693G>A	Intronic		A=0.4169
		rs11676922	chr2:100190478	NC_000002.12:g.100190478T>A			A=0.4864
		rs9653442	chr2:100208905	NC_000002.12:g.100208905C>T	Intronic		T=0.4311
	CD244	rs3766379	chr1:160837925	NG_015991.1:g.29978A>T	Intronic		T=0.4385
		rs6682654	chr1:160839213	NG_015991.1:g.28690C>A	Intronic		A=0.4038
	TRAF6	rs540386	chr11:36503743	NC_000011.10:g.36503743C>T	Intronic		T=0.1438
	TAGAP	rs4709267	chr6:159035343	NC_000006.12:g.159035343A>G	3'UTR		G=0.2210
		rs1738074	chr6:159044945	NC_000006.12:g.159044945T>C	5'UTR		C=0.4663
		rs394581	chr6:159061489	NC_000006.12:g.159061489C>T			C=0.2550
	REL	rs702873	chr2:60854407	NC_000002.12:g.60854407C>T	Intronic		T=0.2488
		rs13031237	chr2:60908994	NC_000002.12:g.60908994G>T	Intronic		T=0.1280
	CD28	rs1980422	chr2:203745673	NC_000002.12:g.203745673C>A			C=0.1715
		rs2140148	chr2:203707417	NG_029618.1:g.5943A>C	Intronic		C=0.0927
	BLK	rs2736340	chr8:11486464	NC_000008.11:g.11486464C>T			T=0.3614
		rs13277113	chr8:11491677	NG_023543.1:g.2666G>A			A=0.3576
		rs2248932	chr8:11534141	NG_023543.1:g.45130A>G	Intronic		G=0.4934
	ATG5/PRDM1	rs548234	chr6:106120159	NC_000006.12:g.106120159C>T			C=0.2029
	PTPRC	rs10919563	chr1:198731313	NG_007730.1:g.97218G>A	Intronic		A=0.2728
	FCGR2A	rs1801274	chr1:161509955	NG_012066.2:g.9541A>G	missense	H167R	G=0.4417
	CD2	rs11586238	chr1:116720516	NC_000001.11:g.116720516C>G			G=0.1520

		rs624988	chr1:116721168	NC_000001.11:g.116721168T>A			T=0.4794
		rs798036	chr1:116766208	NG_050908.1:g.16779T>A	Intronic		A=0.2109
	FCGR2B	rs1050501	chr1:161674008	NG_023318.1:g.15894T>C	missense	I232T	C=0.1859
	SPRED2	rs871974	chr2:65330153	NC_000002.12:g.65330153T>C	Intron		C=0.3608
	IRF5	rs10488631	chr7:128954129	NC_000007.14:g.128954129T>C	3' Near Gene		C=0.0591
		rs2004640	chr7:128938247	NG_012306.1:g.5308G>T	Splice Donor		T=0.4135
		rs2070197	chr7:128948946	NG_012306.1:g.16007T>C	3'UTR		C=0.0591
		rs10954213	chr7:128949373	NG_012306.1:g.16434G>A	3'UTR		G=0.4641
		rs729302	chr7:128928906	NC_000007.14:g.128928906A>C			C=0.2624
	CCR6	rs3093023	chr6:167120802	NC_000006.12:g.167120802G>A	Intronic		A=0.3209
		rs3093024	chr6:167119305	NC_000006.12:g.167119305A>G	Intronic		A=0.3816
		rs6907666	chr6:167109907	NC_000006.12:g.167109907A>G	5' Near Gene		A=0.4141
		rs1331301	chr6:167089150	NC_000006.12:g.167089150A>C			C=0.3423
		rs1556413	chr6:167111255	NC_000006.12:g.167111255G>A	5'Near Gene		A=0.3882
	ANKRD55	rs926657	chr6:159042420	NC_000006.12:g.159042420C>T	Intronic		T=0.1518
		rs9295089	chr6:159042932	NC_000006.12:g.159042932T>C	Intronic		C=0.1695
		rs212402	chr6:159051263	NC_000006.12:g.159051263G>A	Intronic		G=0.2680
	IRAK1	rs3027898	chrX:154010439	NC_000023.11:g.154010439C>A	3' Near gene		A=0.4840
		rs1059703	chrX:154013378	NC_000023.11:g.154013378G>A	missense	S532L	G=0.4832
		rs1059702	chrX:154018741	NC_000023.11:g.154018741A>G	missense	F196S	A=0.3711
	IL6R	rs2228145	chr1:154454494	NG_012087.1:g.54302A>C	missense	D358A	C=0.2931
		rs4537545	chr1:154446403	NG_012087.1:g.46211C>T	Intronic		T=0.4491
		rs4845617	chr1:154405422	NG_012087.1:g.5230G>A	5'UTR		A=0.3804
		rs4329505	chr1:154459944	NG_012087.1:g.59752T>C	Intronic		C=0.2175
	IKZF3	rs2872507	chr17:39884510	NC_000017.11:g.39884510G>A			A=0.3261
	GATA3	rs569421	chr10:8066629	NG_015859.1:g.16926T>C	Intronic		C=0.2953
		rs444929	chr10:8068061	NG_015859.1:g.18358C>G	Intronic		C=0.1358
		rs3802604	chr10:8060309	NG_015859.1:g.10606G>A	Intronic		G=0.4778
	CDF2	rs657075	chr5:132094425	NC_000005.10:g.132094425G>A			A=0.1252
	CD83	rs12529514	chr6:14096427	NC_000006.12:g.14096427T>C			C=0.0857

	B3GNT2	rs11900673	chr2:62225526	NC_000002.12:g.62225526C>T			T=0.1731
		rs4672495	chr2:62294109	NC_000002.12:g.62294109G>T			G=0.1951
	ARID5B	rs10821944	chr10:62025330	NG_030027.1:g.129077G>T	Intronic		G=0.3590
	ARAP1	rs3781913	chr11:72662452	NC_000011.10:g.72662452T>G	Intronic		G=0.4734
	AIRE	rs2075876	chr21:44289270	NG_009556.1:g.8391G>A	Intronic		A=0.2626
		rs933150	chr21:44292705	NG_009556.1:g.11826G>A	Intronic		A=0.2452
		rs760426	chr21:44295931	NG_034033.1:g.898A>G	Intronic		G=0.1989
		rs878081	chr21:44288394	NG_009556.1:g.7515C>T	Syn Codon		T=0.1332
		rs1800520	chr21:44290023	NG_009556.1:g.9144C>G	missense	S278R	G=0.2282
	ANXA3	rs2867461	chr4:78592061	NC_000004.12:g.78592061A>G	Intronic		A=0.4475
	TYK2	rs34536443	chr19:10352442	NG_007872.1:g.33131C>G	missense	P1104A	C=0.0102
	TNFRSF 14	rs3890745	chr1:2622185	NC_000001.11:g.2622185T>C	Intronic		C=0.4846
		rs6684865	chr1:2614790	NC_000001.11:g.2614790G>A	Intronic		A=0.4780
		rs11585747	chr1:2533656	NC_000001.11:g.2533656C>T			T=0.3458
	RTKN2	rs3125734	chr10:62198353	NC_000010.11:g.62198353T>C	missense	H462R	T=0.4111
	RUNX1	rs2268277	chr21:34809752	NC_000021.9:g.34809752G>C	Intronic		C=0.4255
	RASGRP 1	rs8043085	chr15:38535939	NC_000015.10:g.38535939G>T	Intronic		T=0.2853
	PTPN2	rs2847297	chr18:12797695	NC_000018.10:g.12797695A>G	Intronic		G=0.3782
		rs2542151	chr18:12779948	NC_000018.10:g.12779948G>T			G=0.1741
	PLD4	rs2841277	chr14:104924668	NC_000014.9:g.104924668C>T	5'Near Gene		T=0.4271
		rs2841280	chr14:104927219	NC_000014.9:g.104927219G>C	missense	E34Q	C=0.4119
	NFKBIE	rs2233434	chr6:44265183	NC_000006.12:g.44265183A>G	missense	V194A	G=0.0669
		rs2233433	chr6:44265240	NC_000006.12:g.44265240G>A	Missense	P175L	A=0.0529
	IRF8	rs2280381	chr16:85985027	NC_000016.10:g.85985027C>T			C=0.2684
		rs13330176	chr16:85985481	NC_000016.10:g.85985481T>A			A=0.3043
	SH2B3	rs3184504	chr12:111446804	NG_021216.1:g.45857T>A	missense	W262R	T=0.1474
	ILF3	rs147622113	chr19:10661265	NC_000019.10:g.10661265C>T	Intronic		T=0.0048
	FADS2	rs968567	chr11:61828092	NC_000011.10:g.61828092C>T	Intronic		T=0.0527
		rs968567	chr11:61828092	NC_000011.10:g.61828092C>T	5'UTR		T=0.0527
	FADS3	rs76599700	chr11:61877235	NC_000011.10:g.61877235C>T	Intronic		T=0.0833
	UTS2	rs228702	chr1:7885460	NC_000001.11:g.7885460A>G	5'Near Gene		A=0.3804

	CDK5RAP2	rs12379034	chr9:120400388	NC_000009.12:g.120400388A>G	Intronic		G=0.2226
	JAZF1	rs864745	chr7:28140937	NC_000007.14:g.28140937T>C	Intronic		C=0.3027
	IRF4	rs9328192	chr6:434364	NC_000006.12:g.434364A>G			G=0.4377
	ETV7	rs879036	chr6:36382113	NC_000006.12:g.36382113T>C	Intronic		T=0.1603
	ETS1	rs1128334	chr11:128459064	NC_000011.10:g.128459064C>T	3'UTR		T=0.1713
		rs73013527	chr11:128627057	NC_000011.10:g.128627057C>T			T=0.3271
	EOMES	rs9880772	chr3:27736288	NC_000003.12:g.27736288G>A			G=0.2961
	COG6	rs9603612	chr13:39760715	NC_000013.11:g.39760715C>G	Intronic		G=0.2716
		rs7993214	chr13:39776775	NC_000013.11:g.39776775T>C	Intronic		T=0.2985
	CDK6	rs42041	chr7:92617430	NC_000007.14:g.92617430C>G	Intronic		G=0.1480
	CD226	rs763361	chr18:69864406	NC_000018.10:g.69864406T>C	missense	S307G	C=0.4694
	ZNF438	rs793108	chr10:31126177	NC_000010.11:g.31126177C>T	3'Near Gene		T=0.3413
	WDFY4	rs7097397	chr10:48817351	NC_000010.11:g.48817351G>A	missense	R181G	A=0.3586
	YDJC	rs2298428	chr22:21628603	NC_000022.11:g.21628603C>T	missense	A263T	T=0.2248
	UBE2L3	rs5754217	chr22:21585386	NC_000022.11:g.21585386G>T	Intronic		T=0.4141
	UBASH3A	rs11203203	chr21:42416077	NC_000021.9:g.42416077G>A	Intronic		A=0.1979
		rs3788013	chr21:42421219	NC_000021.9:g.42421219C>A	Intronic		A=0.3964
	TEC	rs4694890	chr4:48224250	NC_000004.12:g.48224250A>C	Intronic		C=0.4030
		rs2089510	chr4:48174206	NC_000004.12:g.48174206G>A	Intronic		G=0.2444
	SYNGR1	rs909685	chr22:39351666	NC_000022.11:g.39351666T>A	Intronic		T=0.4938
	RAD51B	rs3784099	chr14:68283210	NC_000014.9:g.68283210G>A	Intronic		A=0.3968
		rs911263	chr14:68286876	NC_000014.9:g.68286876C>T	Intronic		C=0.4000
	PRKCH	rs912620	chr14:61410346	NC_000014.9:g.61410346T>G	Intronic		G=0.4179
		rs959728	chr14:61467300	NC_000014.9:g.61467300C>T	Intronic		C=0.3478
		rs2230500	chr14:61457521	NC_000014.9:g.61457521G>A	missense	V374I	A=0.0605
		rs3783782	chr14:61473957	NC_000014.9:g.61473957G>A	Intronic		A=0.0655
	PPIL4	rs9498368	chr6:149513942	NC_000006.12:g.149513942G>A	Intronic		A=0.4173
	PLCL2	rs4535211	chr3:17031505	NC_000003.12:g.17031505A>G	Intronic		A=0.3536
	P2RY10	rs10465436	chrX:79216145	NC_000023.11:g.79216145G>A	Intronic		A=0.1881
	MTF1	rs67704103	chr1:37825665	NC_000001.11:g.37825665C>T	Intronic		T=0.1725

Table S2: Clinical associations of reported SNPs with RA patients.

Gene	SNP ID	Type	Association with anti-CCP	Association with RF factor	Other clinical symptoms	Ref.
PTPN22	rs33996649	missense	-ve	-ve	Nil	1
	rs3765598	Intronic	Nil	-ve	Nil	2
	rs3811021	3’UTR				
	rs1217414	Intronic				
	rs2476601	missense				
PADI4	rs11203366	missense	Nil	Nil	Nil	3
	rs11203367	misense				
FCRL3	rs7528684	Near 5’Gene	Nil	+ve	erosive	4
	rs11264799	Near 5’Gene				
	rs945635	5’UTR				
	rs3761959	Intronic				
CTLA4	rs231779	Intronic	+ve	Nil	Nil	5
	rs231777	Intronic				
	rs231775	missense				
	rs5742909	Near 5’Gene				
TRAF1/C5	rs10818488	3’UTR	Nil	Nil	Nil	6
	rs3761847	Intonic				
	rs10760130					
	rs2900180	Intronic				
TNFAIP3	rs5029941	missense	-ve	Nil	Nil	7
	rs2230926	missense				
	rs5029930	Intronic				
	rs5029937	Intronic				
	rs5029939	Intronic				
	rs13207033					
	rs10499194					
	rs6920220					
STAT4	rs7574865	Intronic	Nil	+ve	High ESR<br>High DAS28	8,9
IL2-IL21	rs6822844		Nil	Nil	Nil	10

	rs17388568	Intronic				
	rs907715	Intronic				
IL2RB	rs743777	Intronic	Nil	Nil	Erosion at one year	11
	rs3218253	Intronic				
IL2RA	rs2104286	Intronic	Nil	Nil	High sIL2Rα level	12
CD40	rs1883832	5’UTR	Nil	Nil	Nil	13
	rs4810485	Intronic				
	rs1535045	Intronic				
	rs3765459	Intronic				
CCL21	rs2812378	Near 5’ Gene	-ve	Nil	Nil	14
	rs13293020					
AFF3	rs10865035	Intronic	Nil	Nil	+ve with anti-TNF treatment	15
	rs1160542	Intronic				
	rs11676922					
	rs9653442	Intronic				
CD244	rs3766379	Intronic	Nil	Nil	Nil	16
	rs6682654	Intronic				
TRAF6	rs540386	Intronic	Nil	Nil	Local bone damage	17
TAGAP	rs4709267	3’UTR	Nil	Nil	Nil	18
	rs1738074	5’UTR				
	rs394581					
REL	rs702873	Intronic	Nil	Nil	Nil	19
	rs13031237	Intronic				
CD28	rs1980422		+ve	+ve	Nil	20
	rs2140148	Intronic				
BLK	rs2736340		Nil	Nil	Nil	21
	rs13277113					
	rs2248932	Intronic				
ATG5/PRDM1	rs548234		Nil	Nil	Nil	22
PTPRC	rs10919563	Intronic	Nil	Nil	Poor response to anti-TNF	23

FCGR2A	rs1801274	missense	Nil	Nil	associated with responsiveness to adalimumab	23
CD2	rs11586238		Nil	Nil	Nil	22
	rs624988					
	rs798036	Intronic				
FCGR2B	rs1050501	missense	Nil	Nil	RA remission	24
SPRED2	rs871974	Intron	Nil	Nil	Nil	25
IRF5	rs10488631	3’ Near Gene	+ve	+ve	Nil	20
	rs2004640	Splice Donor				
	rs2070197	3’UTR				
	rs10954213	3’UTR				
	rs729302					
CCR6	rs3093023	Intronic	Nil	Nil	Nil	18
	rs3093024	Intronic				
	rs6907666	5’ Near Gene				
	rs1331301					
	rs1556413	5’Near Gene				
ANKRD55	rs926657	Intronic	+ve	Nil	Nil	26
	rs9295089	Intronic				
	rs212402	Intronic				
IRAK1	rs3027898	3’ Near gene	Nil	Nil	Low disease activity -ve with erosion	27
	rs1059703	missense				
	rs1059702	missense				
IL6R	rs2228145	missense	Nil	Nil	Predictor of good response to LDA	28
	rs4537545	Intronic				
	rs4845617	5’UTR				
	rs4329505	Intronic				
GATA3	rs569421	Intronic	Nil	Nil	Nil	29
	rs444929	Intronic				
	rs3802604	Intronic				
CDF2	rs657075		+ve	Nil	Progressive joint destruction	30
CD83	rs12529514					

B3GNT2	rs11900673				Age of onset<br>Female +ve	
	rs4672495					
ARID5B	rs10821944	Intronic				
ARAP1	rs3781913	Intronic				
AIRE	rs2075876	Intronic	Nil	Nil	Nil	31
	rs933150	Intronic				
	rs760426	Intronic				
	rs878081	Syn Codon				
	rs1800520	missense				
ANXA3	rs2867461	Intronic	+ve	Nil	Progressive joint destruction<br>Age of onset<br>Female +ve	30
TYK2	rs34536443	missense	-ve	-ve	Nil	32
TNFRSF14	rs3890745	Intronic	Nil		Nil	33
	rs6684865	Intronic				
	rs11585747					
RTKN2	rs3125734	missense	Nil	Nil	Enhancement of NF-κB activity	34
RUNX1	rs2268277	Intronic	Nil	Nil	Nil	35
RASGRP1	rs8043085	Intronic	Nil	Nil	Nil	36
PTPN2	rs2847297	Intronic	+ve		Progressive joint destruction<br>Age of onset<br>Female +ve	30
	rs2542151					
PLD4	rs2841277	5’Near Gene				
	rs2841280	missense				
NFKBIE	rs2233434	missense				
	rs2233433	Missense				
IRF8	rs2280381		Nil	Nil	Nil	29
	rs13330176					
SH2B3	rs3184504	missense	Nil	Nil	SH2B3 expression level<br>Plasma levels of high-density lipoprotein cholesterol	37

					Low-density lipoprotein cholesterol<br>TC<br>Hypoxanthine<br>80 proteins	
ILF3	rs147622113	Intronic	Nil	Nil	Nil	38
FADS2	rs968567	Intronic				
	rs968567	5'UTR				
CDK5RAP2	rs12379034	Intronic	+ve	Nil	Nil	39
IRF4	rs9328192		Nil	Nil	Nil	38
ETS1	rs1128334	3'UTR	Nil	Nil	Nil	40
	rs73013527					
COG6	rs9603612	Intronic	Nil	Nil	Nil	41
	rs7993214	Intronic				
CDK6	rs42041	Intronic	Nil	Nil	Nil	42
CD226	rs763361	missense	Nil	Nil	+ve with ant-TNF treatment	15
ZNF438	rs793108	3'Near Gene	Nil			41
WDFY4	rs7097397	missense	+ve	Nil	+ve with CRP	40
UBE2L3	rs5754217	Intronic	Nil	Nil	Nil	40
UBASH3A	rs11203203	Intronic	+ve	Nil	+ve with Autoantibody profiles	43
	rs3788013	Intronic				
SYNGR1	rs909685	Intronic	Nil	Nil	DAS28<br>CRP<br>Erosion	44
RAD51B	rs3784099	Intronic	Nil	Nil	Erosion	45
	rs911263	Intronic				
PRKCH	rs912620	Intronic	Nil	Nil	Nil	46
	rs959728	Intronic				
	rs2230500	missense				
	rs3783782	Intronic				
PLCL2	rs4535211	Intronic	Nil	Nil	Nil	47

Note: -ve shows the absence of association, +ve shows the presence of an association and Nil means that the association was not study in the cited study.

References

1. Rodríguez-Rodríguez, L., Taib, W.R.W., Topless, R., Steer, S., González-Escribano, M.F., Balsa, A., Pascual-Salcedo, D., González-Gay, M.A., Raya, E., Fernandez-Gutierrez, B., González-Álvaro, I., Bottini, N., Witte, T., Viken, M.K., Coenen, M.J.H., van Riel, P.L.C.M., Franke, B., den Heijer, M., Radstake, T.R.D.J., Wordsworth, P., Lie, B.A., Merriman, T.R. and Martín, J. The *PTPN22* R263Q polymorphism is a risk factor for rheumatoid arthritis in Caucasian case–control samples. *Arthritis & Rheumatism*, **63**: 365-372. (2011)doi:10.1002/art.30145
2. Tang, L., Wang, Y., Zheng, S., Bao, M., Zhang, Q., Li, J., PTPN22 polymorphisms, butnot R620W, were associated with the genetic susceptibility of systemic lupus erythematosus and rheumatoid arthritisin a Chinese Han population. *Human Immunology* (2016), doi: http://dx.doi.org/10.1016/j.humimm.2016.04.021
3. C.J. Baños-Hernández et al.,PADI4 polymorphisms and the functional haplotype are associated with increased rheumatoid arthritis susceptibility: A replication study in a Southern Mexican population, *Hum. Immunol*. (2017). http://dx.doi.org/10.1016/j.humimm.2017.05.005.
4. Eyre S, Bowes J, Potter C, Worthington J, Barton A. Association of the FCRL3 gene with rheumatoid arthritis: a further example of population specificity? [published correction appears in Arthritis Res Ther. 2008;10(4):405]. *Arthritis Res Ther*.;**8**(4):R117. (2006) doi:10.1186/ar2006
5. Daha, N.A., Kurreeman, F.A.S., Marques, R.B., Stoeken-Rijsbergen, G., Verduijn, W., Huizinga, T.W.J. and Toes, R.E.M. (2009), Confirmation of *STAT4*, *IL2/IL21*, and *CTLA4* polymorphisms in rheumatoid arthritis. *Arthritis & Rheumatism*, 60: 1255-1260. doi:10.1002/art.24503
6. Nishimoto K, Kochi Y, Ikari K et al. Association study of TRAF1-C5 polymorphisms with susceptibility to rheumatoid arthritis and systemic lupus erythematosus in Japanese. *Ann Rheum Dis*. 2009;69(2):368-373. doi:10.1136/ard.2008.104315.
7. Kim S, Choe J, Bae J et al. TNFAIP3 gene polymorphisms associated with differential susceptibility to rheumatoid arthritis and systemic lupus erythematosus in the Korean population. *Rheumatology*. 2014;53(6):1009-1013. doi:10.1093/rheumatology/ket473.
8. 3. Shen L, Liu R, Zhang H, Huang Y, Sun R, Tang P. Replication study of STAT4 rs7574865 G/T polymorphism and risk of rheumatoid arthritis in a Chinese population. *Gene*. 2013;526(2):259-264. doi:10.1016/j.gene.2013.05.022
9. 4. El-Lebedy D, Raslan H, Ibrahim A, Ashmawy I, El-Aziz S, Mohammed A. Association of STAT4 rs7574865 and PTPN22 rs2476601 polymorphisms with rheumatoid arthritis and non-systemically reacting antibodies in Egyptian patients. *Clin Rheumatol*. 2017;36(9):1981-1987. doi:10.1007/s10067-017-3632-7

10. Hollis-Moffatt JE, Chen-Xu M, Topless R, et al. Only one independent genetic association with rheumatoid arthritis within the KIAA1109-TENR-IL2-IL21 locus in Caucasian sample sets: confirmation of association of rs6822844 with rheumatoid arthritis at a genome-wide level of significance. *Arthritis Res Ther*. 2010;12(3):R116. doi:10.1186/ar3053
11. Ruyssen-Witrand A, Lukas C, Nigon D, et al. Association of IL-2RA and IL-2RB genes with erosive status in early rheumatoid arthritis patients (ESPOIR and RMP cohorts). *Joint Bone Spine*. 2014;81(3):228-234. doi:10.1016/j.jbspin.2013.10.002
12. van Steenbergen HW, van Nies JA, Ruyssen-Witrand A, et al. IL2RA is associated with persistence of rheumatoid arthritis. *Arthritis Res Ther*. 2015;17(1):244. Published 2015 Sep 8. doi:10.1186/s13075-015-0739-6
13. García-Bermúdez M, González-Juanatey C, López-Mejías R, et al. Study of association of CD40-CD154 gene polymorphisms with disease susceptibility and cardiovascular risk in Spanish rheumatoid arthritis patients. *PLoS One*. 2012;7(11):e49214. doi:10.1371/journal.pone.0049214
14. Farragher TM, Plant D, Flynn E, et al. Association of a rheumatoid arthritis susceptibility variant at the CCL21 locus with premature mortality in inflammatory polyarthritis patients. *Arthritis Care Res (Hoboken)*. 2010;62(5):676-682. doi:10.1002/acr.20208
15. Tan RJ, Gibbons LJ, Potter C, et al. Investigation of rheumatoid arthritis susceptibility genes identifies association of AFF3 and CD226 variants with response to anti-tumour necrosis factor treatment [published correction appears in Ann Rheum Dis. 2011 Aug;70(8):1519]. *Ann Rheum Dis*. 2010;69(6):1029-1035. doi:10.1136/ard.2009.118406
16. Suzuki A, Yamada R, Kochi Y, et al. Functional SNPs in CD244 increase the risk of rheumatoid arthritis in a Japanese population. *Nat Genet*. 2008;40(10):1224-1229. doi:10.1038/ng.205
17. Hassine HB, Zemni R, Nacef IB, et al. A TRAF6 genetic variant is associated with low bone mineral density in rheumatoid arthritis. *Clin Rheumatol*. 2019;38(4):1067-1074. doi:10.1007/s10067-018-4362-1
18. Perkins EA, Landis D, Causey ZL, et al. Association of single-nucleotide polymorphisms in CCR6, TAGAP, and TNFAIP3 with rheumatoid arthritis in African Americans. *Arthritis Rheum*. 2012;64(5):1355-1358. doi:10.1002/art.33464
19. Zhang Y, Zhang H, Huang Y, Sun R, Liu R, Wei J. Human leukocyte antigen (HLA)-C polymorphisms are associated with a decreased risk of rheumatoid arthritis. *Mol Biol Rep*. 2014;41(6):4103-4108. doi:10.1007/s11033-014-3280-9
20. Vernerova L, Spoutil F, Vlcek M, et al. A Combination of CD28 (rs1980422) and IRF5 (rs10488631) Polymorphisms Is Associated with Seropositivity in Rheumatoid Arthritis: A Case Control Study. *PLoS One*. 2016;11(4):e0153316. Published 2016 Apr 19. doi:10.1371/journal.pone.0153316
21. Huang H, Huang SC, Hua DJ, Sun QQ, Cen H, Xin XF. Interaction analysis between BLK rs13277113 polymorphism and BANK1 rs3733197 polymorphism, MMEL1/TNFRSF14 rs3890745 polymorphism in determining susceptibility to rheumatoid arthritis. *Autoimmunity*. 2017;50(7):403-408. doi:10.1080/08916934.2017.1377191

22. Raychaudhuri S, Thomson BP, Remmers EF, et al. Genetic variants at CD28, PRDM1 and CD2/CD58 are associated with rheumatoid arthritis risk. *Nat Genet*. 2009;41(12):1313-1318. doi:10.1038/ng.479.
23. Lee YH, Bae SC. Associations between PTPRC rs10919563 A/G and FCGR2A R131H polymorphisms and responsiveness to TNF blockers in rheumatoid arthritis: a meta-analysis. *Rheumatol Int*. 2016;36(6):837-844. doi:10.1007/s00296-016-3476-5
24. Skapenko A, Smolen JS, Kavanaugh A, Arora V, Kupper H, Schulze-Koops H. Genetic markers associated with clinical and radiographic response in adalimumab plus methotrexate- or methotrexate-treated rheumatoid arthritis patients in OPTIMA. *Clin Exp Rheumatol*. 2019;37(5):783-790.
25. López Herráez D, Martínez-Bueno M, Riba L, et al. Rheumatoid arthritis in Latin Americans enriched for Amerindian ancestry is associated with loci in chromosomes 1, 12, and 13, and the HLA class II region. *Arthritis Rheum*. 2013;65(6):1457-1467. doi:10.1002/art.37923
26. Viatte S, Massey J, Bowes J, et al. Replication of Associations of Genetic Loci Outside the HLA Region With Susceptibility to Anti-Cyclic Citrullinated Peptide-Negative Rheumatoid Arthritis. *Arthritis Rheumatol*. 2016;68(7):1603-1613. doi:10.1002/art.39619
27. Shaker OG, El Boghdady NA, El Sayed AE. Association of MiRNA-146a, MiRNA-499, IRAK1 and PADI4 Polymorphisms with Rheumatoid Arthritis in Egyptian Population [published correction appears in Cell Physiol Biochem. 2018;47(6):2631]. *Cell Physiol Biochem*. 2018;46(6):2239-2249. doi:10.1159/000489592
28. Maldonado-Montoro M, Cañadas-Garre M, González-Utrilla A, Ángel Calleja-Hernández M. Influence of IL6R gene polymorphisms in the effectiveness to treatment with tocilizumab in rheumatoid arthritis. *Pharmacogenomics J*. 2018;18(1):167-172. doi:10.1038/tpj.2016.88
29. van Hamburg JP, Tas SW. Molecular mechanisms underpinning T helper 17 cell heterogeneity and functions in rheumatoid arthritis. *J Autoimmun*. 2018;87:69-81. doi:10.1016/j.jaut.2017.12.006
30. Suzuki T, Ikari K, Yano K, et al. PADI4 and HLA-DRB1 are genetic risks for radiographic progression in RA patients, independent of ACPA status: results from the IORRA cohort study. *PLoS One*. 2013;8(4):e61045. doi:10.1371/journal.pone.0061045
31. Shao S, Li XR, Cen H, Yin ZS. Association of AIRE polymorphisms with genetic susceptibility to rheumatoid arthritis in a Chinese population. *Inflammation*. 2014;37(2):495-499. doi:10.1007/s10753-013-9763-3
32. Mohamadhosseini A, Mansouri R, Javinani A, et al. Single Nucleotide Polymorphism of *TYK2* Gene and Susceptibility to Rheumatoid Arthritis in Iranian Population. *Avicenna J Med Biotechnol*. 2019;11(2):187-191.
33. Perdigones N, Vigo AG, Lamas JR, et al. Evidence of epistasis between TNFRSF14 and TNFRSF6B polymorphisms in patients with rheumatoid arthritis. *Arthritis Rheum*. 2010;62(3):705-710. doi:10.1002/art.27292
34. Myouzen K, Kochi Y, Okada Y, et al. Functional variants in NFKBIE and RTKN2 involved in activation of the NF-κB pathway are associated with rheumatoid arthritis in Japanese. *PLoS Genet*. 2012;8(9):e1002949. doi:10.1371/journal.pgen.1002949

35. Takata Y, Inoue H, Sato A, et al. Replication of reported genetic associations of PADI4, FCRL3, SLC22A4 and RUNX1 genes with rheumatoid arthritis: results of an independent Japanese population and evidence from meta-analysis of East Asian studies. *J Hum Genet*. 2008;53(2):163-173. doi:10.1007/s10038-007-0232-4
36. Ruiz-Larrañaga O, Uribarri M, Alcaro MC, et al. Genetic variants associated with rheumatoid arthritis patients and serotypes in European populations. *Clin Exp Rheumatol*. 2016;34(2):236-241.
37. Mo X, Guo Y, Qian Q, Fu M, Zhang H. Phosphorylation-related SNPs influence lipid levels and rheumatoid arthritis risk by altering gene expression and plasma protein levels. *Rheumatology (Oxford)*. 2020;59(4):889-898. doi:10.1093/rheumatology/kez466
38. Laufer VA, Tiwari HK, Reynolds RJ, et al. Genetic influences on susceptibility to rheumatoid arthritis in African-Americans. *Hum Mol Genet*. 2019;28(5):858-874. doi:10.1093/hmg/ddy395
39. Jiang L, Yin J, Ye L, et al. Novel risk loci for rheumatoid arthritis in Han Chinese and congruence with risk variants in Europeans [published correction appears in Arthritis Rheumatol. 2014 Jul;66(7):1881]. *Arthritis Rheumatol*. 2014;66(5):1121-1132. doi:10.1002/art.38353
40. Zhang Y, Bo L, Zhang H, Zhuang C, Liu R. E26 transformation-specific-1 (ETS1) and WDFY family member 4 (WDFY4) polymorphisms in Chinese patients with rheumatoid arthritis. *Int J Mol Sci*. 2014;15(2):2712-2721. Published 2014 Feb 17. doi:10.3390/ijms15022712
41. Thompson SD, Sudman M, Ramos PS, et al. The susceptibility loci juvenile idiopathic arthritis shares with other autoimmune diseases extend to PTPN2, COG6, and ANGPT1. *Arthritis Rheum*. 2010;62(11):3265-3276. doi:10.1002/art.27688
42. Orozco G, Eyre S, Hinks A, et al. Association of CD40 with rheumatoid arthritis confirmed in a large UK case-control study [published correction appears in Ann Rheum Dis. 2011 Aug;70(8):1519]. *Ann Rheum Dis*. 2010;69(5):813-816. doi:10.1136/ard.2009.109579
43. Yang XK, Liu J, Chen SY, et al. UBASH3A gene polymorphisms and expression profile in rheumatoid arthritis. *Autoimmunity*. 2019;52(1):21-26. doi:10.1080/08916934.2019.1581773
44. Liu D, Liu J, Cui G, Yang H, Cao T, Wang L. Evaluation of the association of *UBASH3A* and *SYNGR1* with rheumatoid arthritis and disease activity and severity in Han Chinese. *Oncotarget*. 2017;8(61):103385-103392. Published 2017 Oct 17. doi:10.18632/oncotarget.21875
45. Zhi L, Yao S, Ma W, et al. Polymorphisms of RAD51B are associated with rheumatoid arthritis and erosion in rheumatoid arthritis patients. *Sci Rep*. 2017;7:45876. Published 2017 Mar 31. doi:10.1038/srep45876
46. Zhuang Y, Di Y, Huang L, Zhu J. PRKCH polymorphism is associated with rheumatoid arthritis in a Chinese population. *Biosci Trends*. 2020;13(6):556-561. doi:10.5582/bst.2019.01247
47. Arismendi, M., Giraud, M., Ruzehaji, N. *et al.* Identification of *NF-κB* and *PLCL2* as new susceptibility genes and highlights on a potential role of *IRF8* through interferon signature modulation in systemic sclerosis. *Arthritis Res Ther* **17,** 71 (2015). https://doi.org/10.1186/s13075-015-0572-y

S3 Table:

Gene	SNP ID	Residual change	PhD-SNP	SNP & GO	PolyPhen-2	PROVEAN	SIFT
PTPN22	rs33996649	R263Q	Neutral	Neutral	Benign	Neutral	Tolerated
	rs2476601	R620W	Neutral	Neutral	Benign	Deleterious	Deleterious
PADI4	rs11203366	G55S	Neutral	Neutral	Benign	Neutral	Tolerated
	rs11203367	V82A	Neutral	Neutral	Benign	Neutral	Tolerated
CTLA4	rs231775	T17A	Neutral	Neutral	Benign	Neutral	Tolerated
TNFAIP 3	rs5029941	A125V	Neutral	Neutral	Probably Damaging	Neutral	Deleterious
	rs2230926	F127S	Neutral	Neutral	Possibly Damaging	Deleterious	Tolerated
FCGR2A	rs1801274	H167R	Neutral	Neutral	Benign	Deleterious	Tolerated
FCGR2B	rs1050501	I232T	Neutral	Neutral	Benign	Neutral	Tolerated
IRAK1	rs1059703	S532L	Neutral	Neutral	Benign	Neutral	Tolerated
	rs1059702	F196S	Neutral	Neutral	Benign	Neutral	Tolerated
IL6R	rs2228145	D358A	Neutral	Neutral	Benign	Neutral	Tolerated
AIRE	rs1800520	S278R	Neutral	Neutral	Benign	Neutral	Tolerated
TYK2	rs34536443	P1104A	Neutral	Neutral	Probably Damaging	Deleterious	Deleterious
RTKN2	rs3125734	H462R	Neutral	Neutral	Benign	Neutral	Tolerated
PLD4	rs2841280	E34Q	Neutral	Neutral	Benign	Neutral	Tolerated
NFKBIE	rs2233434	V194A	Neutral	Neutral	Benign	Neutral	Tolerated
	rs2233433	P175L	Neutral	Neutral	Benign	Neutral	Tolerated
SH2B3	rs3184504	W262R	Neutral	Neutral	Benign	Neutral	Tolerated
CD226	rs763361	S307G	Neutral	Neutral	Benign	Neutral	Tolerated
WDFY4	rs7097397	R1816Q	Neutral	Neutral	Possibly Damaging	Neutral	Tolerated
YDJC	rs2298428	A263T	Neutral	Neutral	Possibly Damaging	Neutral	Tolerated
PRKCH	rs2230500	V374I	Neutral	Neutral	Benign	Neutral	Tolerated

S4 Table:

node1	node2	node1 accession	node2 accession	score
AFF3	ANKRD55	ENSP00000386834	ENSP00000342295	0.559
AFF3	BLK	ENSP00000386834	ENSP00000259089	0.430
AFF3	CD226	ENSP00000386834	ENSP00000280200	0.414
AFF3	IL2RA	ENSP00000386834	ENSP00000369293	0.422
AFF3	PTPN2	ENSP00000386834	ENSP00000311857	0.419
AFF3	PTPN22	ENSP00000386834	ENSP00000352833	0.548
AFF3	SPRED2	ENSP00000386834	ENSP00000348753	0.433
AFF3	STAT4	ENSP00000386834	ENSP00000376134	0.473
AFF3	TNFAIP3	ENSP00000386834	ENSP00000481570	0.430
AFF3	TRAF1	ENSP00000386834	ENSP00000362994	0.457
AFF3	UTS2	ENSP00000386834	ENSP00000054668	0.536
AIRE	CD40	ENSP00000291582	ENSP00000361359	0.461
AIRE	CTLA4	ENSP00000291582	ENSP00000303939	0.530
AIRE	IRF8	ENSP00000291582	ENSP00000268638	0.542
AIRE	PTPN22	ENSP00000291582	ENSP00000352833	0.530
AIRE	PTPRC	ENSP00000291582	ENSP00000411355	0.416
ANKRD55	AFF3	ENSP00000342295	ENSP00000386834	0.559
ANKRD55	ARAP1	ENSP00000342295	ENSP00000377233	0.455
ANKRD55	BLK	ENSP00000342295	ENSP00000259089	0.452
ANKRD55	IRF5	ENSP00000342295	ENSP00000349770	0.424
ANKRD55	PTPN22	ENSP00000342295	ENSP00000352833	0.520
ANKRD55	SPRED2	ENSP00000342295	ENSP00000348753	0.475
ANKRD55	STAT4	ENSP00000342295	ENSP00000376134	0.438
ANKRD55	TAGAP	ENSP00000342295	ENSP00000356033	0.491
ANKRD55	TNFAIP3	ENSP00000342295	ENSP00000481570	0.502
ARAP1	ANKRD55	ENSP00000377233	ENSP00000342295	0.455
ARAP1	B3GNT2	ENSP00000377233	ENSP00000305595	0.474
ARAP1	JAZF1	ENSP00000377233	ENSP00000283928	0.543

ARAP1	PLD4	ENSP00000377233	ENSP00000376372	0.494
ARID5B	B3GNT2	ENSP00000279873	ENSP00000305595	0.451
ARID5B	GATA3	ENSP00000279873	ENSP00000368632	0.444
ATG5	TRAF6	ENSP00000358072	ENSP00000433623	0.489
ATG5	WDFY4	ENSP00000358072	ENSP00000320563	0.542
B3GNT2	ARAP1	ENSP00000305595	ENSP00000377233	0.474
B3GNT2	ARID5B	ENSP00000305595	ENSP00000279873	0.451
B3GNT2	PLD4	ENSP00000305595	ENSP00000376372	0.564
BLK	AFF3	ENSP00000259089	ENSP00000386834	0.430
BLK	ANKRD55	ENSP00000259089	ENSP00000342295	0.452
BLK	CD40	ENSP00000259089	ENSP00000361359	0.475
BLK	CTLA4	ENSP00000259089	ENSP00000303939	0.400
BLK	ETS1	ENSP00000259089	ENSP00000376436	0.400
BLK	FCGR2A	ENSP00000259089	ENSP00000271450	0.631
BLK	FCGR2B	ENSP00000259089	ENSP00000351497	0.530
BLK	IKZF3	ENSP00000259089	ENSP00000344544	0.482
BLK	IRF5	ENSP00000259089	ENSP00000349770	0.689
BLK	PTPN22	ENSP00000259089	ENSP00000352833	0.761
BLK	PTPRC	ENSP00000259089	ENSP00000411355	0.433
BLK	RUNX1	ENSP00000259089	ENSP00000300305	0.902
BLK	STAT4	ENSP00000259089	ENSP00000376134	0.707
BLK	TNFAIP3	ENSP00000259089	ENSP00000481570	0.623
BLK	TRAF1	ENSP00000259089	ENSP00000362994	0.468
BLK	UBE2L3	ENSP00000259089	ENSP00000400906	0.585
BLK	WDFY4	ENSP00000259089	ENSP00000320563	0.636
C5	CCL21	ENSP00000223642	ENSP00000259607	0.903
C5	TRAF1	ENSP00000223642	ENSP00000362994	0.473
CCL21	C5	ENSP00000259607	ENSP00000223642	0.903
CCL21	CD28	ENSP00000259607	ENSP00000324890	0.455
CCL21	CD40	ENSP00000259607	ENSP00000361359	0.575

CCL21	CD83	ENSP00000259607	ENSP00000368450	0.547
CCL21	CTLA4	ENSP00000259607	ENSP00000303939	0.531
CCL21	PTPRC	ENSP00000259607	ENSP00000411355	0.548
CCL21	TNFRSF14	ENSP00000259607	ENSP00000347948	0.463
CD2	CD226	ENSP00000358490	ENSP00000280200	0.533
CD2	CD244	ENSP00000358490	ENSP00000357012	0.687
CD2	CD28	ENSP00000358490	ENSP00000324890	0.980
CD2	CD40	ENSP00000358490	ENSP00000361359	0.548
CD2	CD83	ENSP00000358490	ENSP00000368450	0.428
CD2	CTLA4	ENSP00000358490	ENSP00000303939	0.842
CD2	FCGR2A	ENSP00000358490	ENSP00000271450	0.473
CD2	FCGR2B	ENSP00000358490	ENSP00000351497	0.483
CD2	IL2RA	ENSP00000358490	ENSP00000369293	0.677
CD2	IL2RB	ENSP00000358490	ENSP00000216223	0.779
CD2	IRF4	ENSP00000358490	ENSP00000370343	0.416
CD2	PTPRC	ENSP00000358490	ENSP00000411355	0.915
CD2	STAT4	ENSP00000358490	ENSP00000376134	0.409
CD2	TAGAP	ENSP00000358490	ENSP00000356033	0.595
CD226	AFF3	ENSP00000280200	ENSP00000386834	0.414
CD226	CD2	ENSP00000280200	ENSP00000358490	0.533
CD226	CD244	ENSP00000280200	ENSP00000357012	0.705
CD226	CD28	ENSP00000280200	ENSP00000324890	0.474
CD226	CTLA4	ENSP00000280200	ENSP00000303939	0.569
CD226	GATA3	ENSP00000280200	ENSP00000368632	0.496
CD226	IL2RA	ENSP00000280200	ENSP00000369293	0.508
CD226	IL2RB	ENSP00000280200	ENSP00000216223	0.489
CD226	PTPN22	ENSP00000280200	ENSP00000352833	0.475
CD226	PTPRC	ENSP00000280200	ENSP00000411355	0.415
CD226	SH2B3	ENSP00000280200	ENSP00000345492	0.476
CD226	STAT4	ENSP00000280200	ENSP00000376134	0.426

CD244	CD2	ENSP00000357012	ENSP00000358490	0.687
CD244	CD226	ENSP00000357012	ENSP00000280200	0.705
CD244	CD28	ENSP00000357012	ENSP00000324890	0.496
CD244	CTLA4	ENSP00000357012	ENSP00000303939	0.602
CD244	EOMES	ENSP00000357012	ENSP00000388620	0.465
CD244	FCGR2B	ENSP00000357012	ENSP00000351497	0.439
CD244	IL2RB	ENSP00000357012	ENSP00000216223	0.617
CD244	PADI4	ENSP00000357012	ENSP00000364597	0.412
CD244	PTPRC	ENSP00000357012	ENSP00000411355	0.736
CD244	WDFY4	ENSP00000357012	ENSP00000320563	0.403
CD28	CCL21	ENSP00000324890	ENSP00000259607	0.455
CD28	CD2	ENSP00000324890	ENSP00000358490	0.980
CD28	CD226	ENSP00000324890	ENSP00000280200	0.474
CD28	CD244	ENSP00000324890	ENSP00000357012	0.496
CD28	CD40	ENSP00000324890	ENSP00000361359	0.820
CD28	CD83	ENSP00000324890	ENSP00000368450	0.586
CD28	CTLA4	ENSP00000324890	ENSP00000303939	0.930
CD28	EOMES	ENSP00000324890	ENSP00000388620	0.547
CD28	FCGR2A	ENSP00000324890	ENSP00000271450	0.597
CD28	FCGR2B	ENSP00000324890	ENSP00000351497	0.610
CD28	GATA3	ENSP00000324890	ENSP00000368632	0.696
CD28	GPR29	ENSP00000324890	ENSP00000343952	0.662
CD28	IL2RA	ENSP00000324890	ENSP00000369293	0.708
CD28	IL2RB	ENSP00000324890	ENSP00000216223	0.672
CD28	IL6R	ENSP00000324890	ENSP00000357470	0.459
CD28	IRF4	ENSP00000324890	ENSP00000370343	0.576
CD28	PRDM1	ENSP00000324890	ENSP00000358092	0.557
CD28	PTPN22	ENSP00000324890	ENSP00000352833	0.408
CD28	PTPRC	ENSP00000324890	ENSP00000411355	0.860
CD28	RASGRP1	ENSP00000324890	ENSP00000310244	0.434

CD28	REL	ENSP00000324890	ENSP00000295025	0.564
CD28	STAT4	ENSP00000324890	ENSP00000376134	0.551
CD28	TNFRSF14	ENSP00000324890	ENSP00000347948	0.558
CD28	TRAF6	ENSP00000324890	ENSP00000433623	0.434
CD40	AIRE	ENSP00000361359	ENSP00000291582	0.461
CD40	BLK	ENSP00000361359	ENSP00000259089	0.475
CD40	CCL21	ENSP00000361359	ENSP00000259607	0.575
CD40	CD2	ENSP00000361359	ENSP00000358490	0.548
CD40	CD28	ENSP00000361359	ENSP00000324890	0.820
CD40	CD83	ENSP00000361359	ENSP00000368450	0.878
CD40	CTLA4	ENSP00000361359	ENSP00000303939	0.842
CD40	FCGR2A	ENSP00000361359	ENSP00000271450	0.678
CD40	FCGR2B	ENSP00000361359	ENSP00000351497	0.693
CD40	FCRL3	ENSP00000361359	ENSP00000357167	0.412
CD40	GATA3	ENSP00000361359	ENSP00000368632	0.442
CD40	GPR29	ENSP00000361359	ENSP00000343952	0.567
CD40	IL2RA	ENSP00000361359	ENSP00000369293	0.599
CD40	IL2RB	ENSP00000361359	ENSP00000216223	0.534
CD40	IL6R	ENSP00000361359	ENSP00000357470	0.431
CD40	IRAK1	ENSP00000361359	ENSP00000358997	0.549
CD40	IRF4	ENSP00000361359	ENSP00000370343	0.746
CD40	IRF5	ENSP00000361359	ENSP00000349770	0.475
CD40	IRF8	ENSP00000361359	ENSP00000268638	0.522
CD40	PRDM1	ENSP00000361359	ENSP00000358092	0.578
CD40	PTPN22	ENSP00000361359	ENSP00000352833	0.570
CD40	PTPRC	ENSP00000361359	ENSP00000411355	0.736
CD40	REL	ENSP00000361359	ENSP00000295025	0.801
CD40	STAT4	ENSP00000361359	ENSP00000376134	0.533
CD40	TNFAIP3	ENSP00000361359	ENSP00000481570	0.634
CD40	TRAF1	ENSP00000361359	ENSP00000362994	0.994

CD40	TRAF6	ENSP00000361359	ENSP00000433623	0.994
CD40	TYK2	ENSP00000361359	ENSP00000431885	0.417
CD83	CCL21	ENSP00000368450	ENSP00000259607	0.547
CD83	CD2	ENSP00000368450	ENSP00000358490	0.428
CD83	CD28	ENSP00000368450	ENSP00000324890	0.586
CD83	CD40	ENSP00000368450	ENSP00000361359	0.878
CD83	CTLA4	ENSP00000368450	ENSP00000303939	0.587
CD83	FCGR2A	ENSP00000368450	ENSP00000271450	0.533
CD83	FCGR2B	ENSP00000368450	ENSP00000351497	0.525
CD83	GPR29	ENSP00000368450	ENSP00000343952	0.475
CD83	IRF8	ENSP00000368450	ENSP00000268638	0.436
CD83	PTPRC	ENSP00000368450	ENSP00000411355	0.563
CD83	TNFAIP3	ENSP00000368450	ENSP00000481570	0.444
CDK5RAP2	REL	ENSP00000343818	ENSP00000295025	0.544
CDK6	RUNX1	ENSP00000265734	ENSP00000300305	0.963
COG6	RAD51B	ENSP00000397441	ENSP00000419471	0.449
COG6	UBASH3A	ENSP00000397441	ENSP00000317327	0.446
CTLA4	AIRE	ENSP00000303939	ENSP00000291582	0.530
CTLA4	BLK	ENSP00000303939	ENSP00000259089	0.400
CTLA4	CCL21	ENSP00000303939	ENSP00000259607	0.531
CTLA4	CD2	ENSP00000303939	ENSP00000358490	0.842
CTLA4	CD226	ENSP00000303939	ENSP00000280200	0.569
CTLA4	CD244	ENSP00000303939	ENSP00000357012	0.602
CTLA4	CD28	ENSP00000303939	ENSP00000324890	0.930
CTLA4	CD40	ENSP00000303939	ENSP00000361359	0.842
CTLA4	CD83	ENSP00000303939	ENSP00000368450	0.587
CTLA4	EOMES	ENSP00000303939	ENSP00000388620	0.583
CTLA4	FCGR2A	ENSP00000303939	ENSP00000271450	0.652
CTLA4	FCGR2B	ENSP00000303939	ENSP00000351497	0.654

CTLA4	FCRL3	ENSP00000303939	ENSP00000357167	0.554
CTLA4	GATA3	ENSP00000303939	ENSP00000368632	0.832
CTLA4	GPR29	ENSP00000303939	ENSP00000343952	0.677
CTLA4	IL2RA	ENSP00000303939	ENSP00000369293	0.759
CTLA4	IL2RB	ENSP00000303939	ENSP00000216223	0.709
CTLA4	IL6R	ENSP00000303939	ENSP00000357470	0.528
CTLA4	IRF4	ENSP00000303939	ENSP00000370343	0.618
CTLA4	IRF5	ENSP00000303939	ENSP00000349770	0.442
CTLA4	IRF8	ENSP00000303939	ENSP00000268638	0.405
CTLA4	PADI4	ENSP00000303939	ENSP00000364597	0.441
CTLA4	PRDM1	ENSP00000303939	ENSP00000358092	0.591
CTLA4	PTPN2	ENSP00000303939	ENSP00000311857	0.448
CTLA4	PTPN22	ENSP00000303939	ENSP00000352833	0.756
CTLA4	PTPRC	ENSP00000303939	ENSP00000411355	0.790
CTLA4	RASGRP1	ENSP00000303939	ENSP00000310244	0.463
CTLA4	REL	ENSP00000303939	ENSP00000295025	0.559
CTLA4	RUNX1	ENSP00000303939	ENSP00000300305	0.922
CTLA4	SH2B3	ENSP00000303939	ENSP00000345492	0.410
CTLA4	STAT4	ENSP00000303939	ENSP00000376134	0.681
CTLA4	TAGAP	ENSP00000303939	ENSP00000356033	0.469
CTLA4	TNFAIP3	ENSP00000303939	ENSP00000481570	0.461
CTLA4	TNFRSF14	ENSP00000303939	ENSP00000347948	0.651
CTLA4	TRAF1	ENSP00000303939	ENSP00000362994	0.514
CTLA4	TRAF6	ENSP00000303939	ENSP00000433623	0.453
CTLA4	TYK2	ENSP00000303939	ENSP00000431885	0.404
CTLA4	UBASH3A	ENSP00000303939	ENSP00000317327	0.425
EOMES	CD244	ENSP00000388620	ENSP00000357012	0.465
EOMES	CD28	ENSP00000388620	ENSP00000324890	0.547
EOMES	CTLA4	ENSP00000388620	ENSP00000303939	0.583
EOMES	ETS1	ENSP00000388620	ENSP00000376436	0.426

EOMES	GATA3	ENSP00000388620	ENSP00000368632	0.811
EOMES	GPR29	ENSP00000388620	ENSP00000343952	0.441
EOMES	IL2RA	ENSP00000388620	ENSP00000369293	0.448
EOMES	IL2RB	ENSP00000388620	ENSP00000216223	0.815
EOMES	IRF4	ENSP00000388620	ENSP00000370343	0.681
EOMES	PRDM1	ENSP00000388620	ENSP00000358092	0.681
EOMES	PTPRC	ENSP00000388620	ENSP00000411355	0.528
EOMES	STAT4	ENSP00000388620	ENSP00000376134	0.491
ETS1	BLK	ENSP00000376436	ENSP00000259089	0.400
ETS1	EOMES	ENSP00000376436	ENSP00000388620	0.426
ETS1	GATA3	ENSP00000376436	ENSP00000368632	0.744
ETS1	IKZF3	ENSP00000376436	ENSP00000344544	0.437
ETS1	IRF4	ENSP00000376436	ENSP00000370343	0.499
ETS1	PRDM1	ENSP00000376436	ENSP00000358092	0.585
ETS1	RUNX1	ENSP00000376436	ENSP00000300305	0.974
ETS1	STAT4	ENSP00000376436	ENSP00000376134	0.472
ETS1	TNFAIP3	ENSP00000376436	ENSP00000481570	0.432
ETS1	WDFY4	ENSP00000376436	ENSP00000320563	0.440
FCGR2A	BLK	ENSP00000271450	ENSP00000259089	0.631
FCGR2A	CD2	ENSP00000271450	ENSP00000358490	0.473
FCGR2A	CD28	ENSP00000271450	ENSP00000324890	0.597
FCGR2A	CD40	ENSP00000271450	ENSP00000361359	0.678
FCGR2A	CD83	ENSP00000271450	ENSP00000368450	0.533
FCGR2A	CTLA4	ENSP00000271450	ENSP00000303939	0.652
FCGR2A	FCGR2B	ENSP00000271450	ENSP00000351497	0.656
FCGR2A	IL2RA	ENSP00000271450	ENSP00000369293	0.410
FCGR2A	IL2RB	ENSP00000271450	ENSP00000216223	0.465
FCGR2A	IRF5	ENSP00000271450	ENSP00000349770	0.481
FCGR2A	PTPN22	ENSP00000271450	ENSP00000352833	0.478
FCGR2A	PTPRC	ENSP00000271450	ENSP00000411355	0.839

FCGR2A	STAT4	ENSP00000271450	ENSP00000376134	0.402
FCGR2B	BLK	ENSP00000351497	ENSP00000259089	0.530
FCGR2B	CD2	ENSP00000351497	ENSP00000358490	0.483
FCGR2B	CD244	ENSP00000351497	ENSP00000357012	0.439
FCGR2B	CD28	ENSP00000351497	ENSP00000324890	0.610
FCGR2B	CD40	ENSP00000351497	ENSP00000361359	0.693
FCGR2B	CD83	ENSP00000351497	ENSP00000368450	0.525
FCGR2B	CTLA4	ENSP00000351497	ENSP00000303939	0.654
FCGR2B	FCGR2A	ENSP00000351497	ENSP00000271450	0.656
FCGR2B	IL2RA	ENSP00000351497	ENSP00000369293	0.453
FCGR2B	IL2RB	ENSP00000351497	ENSP00000216223	0.487
FCGR2B	IRF8	ENSP00000351497	ENSP00000268638	0.510
FCGR2B	PTPRC	ENSP00000351497	ENSP00000411355	0.865
FCRL3	CD40	ENSP00000357167	ENSP00000361359	0.412
FCRL3	CTLA4	ENSP00000357167	ENSP00000303939	0.554
FCRL3	IL2RA	ENSP00000357167	ENSP00000369293	0.432
FCRL3	PADI4	ENSP00000357167	ENSP00000364597	0.598
FCRL3	PTPN22	ENSP00000357167	ENSP00000352833	0.722
FCRL3	STAT4	ENSP00000357167	ENSP00000376134	0.463
FCRL3	TRAF1	ENSP00000357167	ENSP00000362994	0.404
GATA3	ARID5B	ENSP00000368632	ENSP00000279873	0.444
GATA3	CD226	ENSP00000368632	ENSP00000280200	0.496
GATA3	CD28	ENSP00000368632	ENSP00000324890	0.696
GATA3	CD40	ENSP00000368632	ENSP00000361359	0.442
GATA3	CTLA4	ENSP00000368632	ENSP00000303939	0.832
GATA3	EOMES	ENSP00000368632	ENSP00000388620	0.811
GATA3	ETS1	ENSP00000368632	ENSP00000376436	0.744
GATA3	GPR29	ENSP00000368632	ENSP00000343952	0.598
GATA3	IKZF3	ENSP00000368632	ENSP00000344544	0.449
GATA3	IL2RA	ENSP00000368632	ENSP00000369293	0.559

GATA3	IL2RB	ENSP00000368632	ENSP00000216223	0.517
GATA3	IRF4	ENSP00000368632	ENSP00000370343	0.750
GATA3	PRDM1	ENSP00000368632	ENSP00000358092	0.544
GATA3	PTPRC	ENSP00000368632	ENSP00000411355	0.577
GATA3	RUNX1	ENSP00000368632	ENSP00000300305	0.949
GATA3	STAT4	ENSP00000368632	ENSP00000376134	0.819
GPR29	CCL21	ENSP00000343952	ENSP00000259607	0.695
GPR29	CD28	ENSP00000343952	ENSP00000324890	0.662
GPR29	CD40	ENSP00000343952	ENSP00000361359	0.567
GPR29	CD83	ENSP00000343952	ENSP00000368450	0.475
GPR29	CTLA4	ENSP00000343952	ENSP00000303939	0.677
GPR29	EOMES	ENSP00000343952	ENSP00000388620	0.441
GPR29	GATA3	ENSP00000343952	ENSP00000368632	0.598
GPR29	IL2RA	ENSP00000343952	ENSP00000369293	0.476
GPR29	IL2RB	ENSP00000343952	ENSP00000216223	0.494
GPR29	IL6R	ENSP00000343952	ENSP00000357470	0.428
GPR29	IRF4	ENSP00000343952	ENSP00000370343	0.553
GPR29	IRF8	ENSP00000343952	ENSP00000268638	0.584
GPR29	PTPN22	ENSP00000343952	ENSP00000352833	0.485
GPR29	PTPRC	ENSP00000343952	ENSP00000411355	0.614
GPR29	STAT4	ENSP00000343952	ENSP00000376134	0.479
IKZF3	BLK	ENSP00000344544	ENSP00000259089	0.482
IKZF3	ETS1	ENSP00000344544	ENSP00000376436	0.437
IKZF3	GATA3	ENSP00000344544	ENSP00000368632	0.449
IKZF3	IL2RA	ENSP00000344544	ENSP00000369293	0.930
IKZF3	IL2RB	ENSP00000344544	ENSP00000216223	0.939
IKZF3	IRF4	ENSP00000344544	ENSP00000370343	0.817
IKZF3	IRF5	ENSP00000344544	ENSP00000349770	0.431
IKZF3	IRF8	ENSP00000344544	ENSP00000268638	0.514
IKZF3	P2RY10	ENSP00000344544	ENSP00000171757	0.571

IKZF3	PLD4	ENSP00000344544	ENSP00000376372	0.461
IKZF3	PRDM1	ENSP00000344544	ENSP00000358092	0.448
IKZF3	STAT4	ENSP00000344544	ENSP00000376134	0.414
IL2RA	AFF3	ENSP00000369293	ENSP00000386834	0.422
IL2RA	CD2	ENSP00000369293	ENSP00000358490	0.677
IL2RA	CD226	ENSP00000369293	ENSP00000280200	0.508
IL2RA	CD28	ENSP00000369293	ENSP00000324890	0.708
IL2RA	CD40	ENSP00000369293	ENSP00000361359	0.599
IL2RA	CTLA4	ENSP00000369293	ENSP00000303939	0.759
IL2RA	EOMES	ENSP00000369293	ENSP00000388620	0.448
IL2RA	FCGR2A	ENSP00000369293	ENSP00000271450	0.410
IL2RA	FCGR2B	ENSP00000369293	ENSP00000351497	0.453
IL2RA	FCRL3	ENSP00000369293	ENSP00000357167	0.432
IL2RA	GATA3	ENSP00000369293	ENSP00000368632	0.559
IL2RA	GPR29	ENSP00000369293	ENSP00000343952	0.476
IL2RA	IKZF3	ENSP00000369293	ENSP00000344544	0.930
IL2RA	IL2RB	ENSP00000369293	ENSP00000216223	0.996
IL2RA	IL6R	ENSP00000369293	ENSP00000357470	0.566
IL2RA	IRF4	ENSP00000369293	ENSP00000370343	0.493
IL2RA	IRF8	ENSP00000369293	ENSP00000268638	0.471
IL2RA	PRDM1	ENSP00000369293	ENSP00000358092	0.500
IL2RA	PTPN2	ENSP00000369293	ENSP00000311857	0.535
IL2RA	PTPN22	ENSP00000369293	ENSP00000352833	0.720
IL2RA	PTPRC	ENSP00000369293	ENSP00000411355	0.665
IL2RA	REL	ENSP00000369293	ENSP00000295025	0.457
IL2RA	RUNX1	ENSP00000369293	ENSP00000300305	0.927
IL2RA	SH2B3	ENSP00000369293	ENSP00000345492	0.473
IL2RA	STAT4	ENSP00000369293	ENSP00000376134	0.569
IL2RA	TAGAP	ENSP00000369293	ENSP00000356033	0.438
IL2RA	TNFAIP3	ENSP00000369293	ENSP00000481570	0.440

IL2RA	TRAF1	ENSP00000369293	ENSP00000362994	0.418
IL2RA	TYK2	ENSP00000369293	ENSP00000431885	0.768
IL2RA	UBASH3A	ENSP00000369293	ENSP00000317327	0.499
IL2RB	CD2	ENSP00000216223	ENSP00000358490	0.779
IL2RB	CD226	ENSP00000216223	ENSP00000280200	0.489
IL2RB	CD244	ENSP00000216223	ENSP00000357012	0.617
IL2RB	CD28	ENSP00000216223	ENSP00000324890	0.672
IL2RB	CD40	ENSP00000216223	ENSP00000361359	0.534
IL2RB	CTLA4	ENSP00000216223	ENSP00000303939	0.709
IL2RB	EOMES	ENSP00000216223	ENSP00000388620	0.815
IL2RB	FCGR2A	ENSP00000216223	ENSP00000271450	0.465
IL2RB	FCGR2B	ENSP00000216223	ENSP00000351497	0.487
IL2RB	GATA3	ENSP00000216223	ENSP00000368632	0.517
IL2RB	GPR29	ENSP00000216223	ENSP00000343952	0.494
IL2RB	IKZF3	ENSP00000216223	ENSP00000344544	0.939
IL2RB	IL2RA	ENSP00000216223	ENSP00000369293	0.996
IL2RB	IL6R	ENSP00000216223	ENSP00000357470	0.443
IL2RB	IRF4	ENSP00000216223	ENSP00000370343	0.448
IL2RB	PRDM1	ENSP00000216223	ENSP00000358092	0.504
IL2RB	PTPN22	ENSP00000216223	ENSP00000352833	0.730
IL2RB	PTPRC	ENSP00000216223	ENSP00000411355	0.766
IL2RB	STAT4	ENSP00000216223	ENSP00000376134	0.487
IL2RB	TRAF1	ENSP00000216223	ENSP00000362994	0.422
IL2RB	TYK2	ENSP00000216223	ENSP00000431885	0.685
IL6R	CD28	ENSP00000357470	ENSP00000324890	0.459
IL6R	CD40	ENSP00000357470	ENSP00000361359	0.431
IL6R	CTLA4	ENSP00000357470	ENSP00000303939	0.528
IL6R	GPR29	ENSP00000357470	ENSP00000343952	0.428
IL6R	IL2RA	ENSP00000357470	ENSP00000369293	0.566
IL6R	IL2RB	ENSP00000357470	ENSP00000216223	0.443

IL6R	PTPRC	ENSP00000357470	ENSP00000411355	0.457
IL6R	STAT4	ENSP00000357470	ENSP00000376134	0.469
IL6R	TYK2	ENSP00000357470	ENSP00000431885	0.950
IRAK1	CD40	ENSP00000358997	ENSP00000361359	0.549
IRAK1	IRF4	ENSP00000358997	ENSP00000370343	0.651
IRAK1	IRF5	ENSP00000358997	ENSP00000349770	0.746
IRAK1	PTPN22	ENSP00000358997	ENSP00000352833	0.478
IRAK1	REL	ENSP00000358997	ENSP00000295025	0.655
IRAK1	STAT4	ENSP00000358997	ENSP00000376134	0.476
IRAK1	TNFAIP3	ENSP00000358997	ENSP00000481570	0.691
IRAK1	TRAF1	ENSP00000358997	ENSP00000362994	0.509
IRAK1	TRAF6	ENSP00000358997	ENSP00000433623	0.999
IRF4	CD2	ENSP00000370343	ENSP00000358490	0.416
IRF4	CD28	ENSP00000370343	ENSP00000324890	0.576
IRF4	CD40	ENSP00000370343	ENSP00000361359	0.746
IRF4	CTLA4	ENSP00000370343	ENSP00000303939	0.618
IRF4	EOMES	ENSP00000370343	ENSP00000388620	0.681
IRF4	ETS1	ENSP00000370343	ENSP00000376436	0.499
IRF4	GATA3	ENSP00000370343	ENSP00000368632	0.750
IRF4	GPR29	ENSP00000370343	ENSP00000343952	0.553
IRF4	IKZF3	ENSP00000370343	ENSP00000344544	0.817
IRF4	IL2RA	ENSP00000370343	ENSP00000369293	0.493
IRF4	IL2RB	ENSP00000370343	ENSP00000216223	0.448
IRF4	IRAK1	ENSP00000370343	ENSP00000358997	0.651
IRF4	IRF5	ENSP00000370343	ENSP00000349770	0.923
IRF4	IRF8	ENSP00000370343	ENSP00000268638	0.442
IRF4	PRDM1	ENSP00000370343	ENSP00000358092	0.943
IRF4	PTPRC	ENSP00000370343	ENSP00000411355	0.609
IRF4	REL	ENSP00000370343	ENSP00000295025	0.732
IRF4	STAT4	ENSP00000370343	ENSP00000376134	0.534

IRF4	TNFAIP3	ENSP00000370343	ENSP00000481570	0.467
IRF4	TRAF6	ENSP00000370343	ENSP00000433623	0.651
IRF5	ANKRD55	ENSP00000349770	ENSP00000342295	0.424
IRF5	BLK	ENSP00000349770	ENSP00000259089	0.689
IRF5	CD40	ENSP00000349770	ENSP00000361359	0.475
IRF5	CTLA4	ENSP00000349770	ENSP00000303939	0.442
IRF5	FCGR2A	ENSP00000349770	ENSP00000271450	0.481
IRF5	IKZF3	ENSP00000349770	ENSP00000344544	0.431
IRF5	IRAK1	ENSP00000349770	ENSP00000358997	0.746
IRF5	IRF4	ENSP00000349770	ENSP00000370343	0.923
IRF5	PTPN22	ENSP00000349770	ENSP00000352833	0.720
IRF5	REL	ENSP00000349770	ENSP00000295025	0.415
IRF5	SPRED2	ENSP00000349770	ENSP00000348753	0.416
IRF5	STAT4	ENSP00000349770	ENSP00000376134	0.737
IRF5	TNFAIP3	ENSP00000349770	ENSP00000481570	0.687
IRF5	TRAF1	ENSP00000349770	ENSP00000362994	0.490
IRF5	TRAF6	ENSP00000349770	ENSP00000433623	0.951
IRF5	TYK2	ENSP00000349770	ENSP00000431885	0.555
IRF5	UBE2L3	ENSP00000349770	ENSP00000400906	0.445
IRF5	WDFY4	ENSP00000349770	ENSP00000320563	0.571
IRF8	AIRE	ENSP00000268638	ENSP00000291582	0.542
IRF8	CD40	ENSP00000268638	ENSP00000361359	0.522
IRF8	CD83	ENSP00000268638	ENSP00000368450	0.436
IRF8	CTLA4	ENSP00000268638	ENSP00000303939	0.405
IRF8	FCGR2B	ENSP00000268638	ENSP00000351497	0.510
IRF8	GPR29	ENSP00000268638	ENSP00000343952	0.584
IRF8	IKZF3	ENSP00000268638	ENSP00000344544	0.514
IRF8	IL2RA	ENSP00000268638	ENSP00000369293	0.471
IRF8	IRF4	ENSP00000268638	ENSP00000370343	0.442
IRF8	PLD4	ENSP00000268638	ENSP00000376372	0.442

IRF8	PRDM1	ENSP00000268638	ENSP00000358092	0.565
IRF8	PTPRC	ENSP00000268638	ENSP00000411355	0.631
IRF8	REL	ENSP00000268638	ENSP00000295025	0.442
IRF8	STAT4	ENSP00000268638	ENSP00000376134	0.516
IRF8	TAGAP	ENSP00000268638	ENSP00000356033	0.442
IRF8	TNFAIP3	ENSP00000268638	ENSP00000481570	0.480
IRF8	TRAF6	ENSP00000268638	ENSP00000433623	0.612
IRF8	TYK2	ENSP00000268638	ENSP00000431885	0.494
IRF8	WDFY4	ENSP00000268638	ENSP00000320563	0.597
JAZF1	ARAP1	ENSP00000283928	ENSP00000377233	0.543
NFKBIE	REL	ENSP00000275015	ENSP00000295025	0.992
NFKBIE	TNFAIP3	ENSP00000275015	ENSP00000481570	0.630
NFKBIE	TRAF1	ENSP00000275015	ENSP00000362994	0.531
P2RY10	IKZF3	ENSP00000171757	ENSP00000344544	0.571
P2RY10	PLD4	ENSP00000171757	ENSP00000376372	0.534
P2RY10	PTPN22	ENSP00000171757	ENSP00000352833	0.564
P2RY10	PTPRC	ENSP00000171757	ENSP00000411355	0.446
P2RY10	UTS2	ENSP00000171757	ENSP00000054668	0.902
PADI4	CD244	ENSP00000364597	ENSP00000357012	0.412
PADI4	CTLA4	ENSP00000364597	ENSP00000303939	0.441
PADI4	FCRL3	ENSP00000364597	ENSP00000357167	0.598
PADI4	PTPN22	ENSP00000364597	ENSP00000352833	0.718
PADI4	STAT4	ENSP00000364597	ENSP00000376134	0.575
PADI4	TNFAIP3	ENSP00000364597	ENSP00000481570	0.473
PADI4	TRAF1	ENSP00000364597	ENSP00000362994	0.558
PLD4	ARAP1	ENSP00000376372	ENSP00000377233	0.494
PLD4	B3GNT2	ENSP00000376372	ENSP00000305595	0.564
PLD4	IKZF3	ENSP00000376372	ENSP00000344544	0.461
PLD4	IRF8	ENSP00000376372	ENSP00000268638	0.442
PLD4	P2RY10	ENSP00000376372	ENSP00000171757	0.534

PLD4	PTPN22	ENSP00000376372	ENSP00000352833	0.638
PLD4	WDFY4	ENSP00000376372	ENSP00000320563	0.445
PRDM1	CD28	ENSP00000358092	ENSP00000324890	0.557
PRDM1	CD40	ENSP00000358092	ENSP00000361359	0.578
PRDM1	CTLA4	ENSP00000358092	ENSP00000303939	0.591
PRDM1	EOMES	ENSP00000358092	ENSP00000388620	0.681
PRDM1	ETS1	ENSP00000358092	ENSP00000376436	0.585
PRDM1	GATA3	ENSP00000358092	ENSP00000368632	0.544
PRDM1	IKZF3	ENSP00000358092	ENSP00000344544	0.448
PRDM1	IL2RA	ENSP00000358092	ENSP00000369293	0.500
PRDM1	IL2RB	ENSP00000358092	ENSP00000216223	0.504
PRDM1	IRF4	ENSP00000358092	ENSP00000370343	0.943
PRDM1	IRF8	ENSP00000358092	ENSP00000268638	0.565
PRDM1	PTPRC	ENSP00000358092	ENSP00000411355	0.523
PRDM1	STAT4	ENSP00000358092	ENSP00000376134	0.476
PRDM1	TNFAIP3	ENSP00000358092	ENSP00000481570	0.555
PTPN2	AFF3	ENSP00000311857	ENSP00000386834	0.419
PTPN2	CTLA4	ENSP00000311857	ENSP00000303939	0.448
PTPN2	IL2RA	ENSP00000311857	ENSP00000369293	0.535
PTPN2	SH2B3	ENSP00000311857	ENSP00000345492	0.547
PTPN2	STAT4	ENSP00000311857	ENSP00000376134	0.791
PTPN2	TAGAP	ENSP00000311857	ENSP00000356033	0.543
PTPN2	TYK2	ENSP00000311857	ENSP00000431885	0.901
PTPN2	UBASH3A	ENSP00000311857	ENSP00000317327	0.471
PTPN22	AFF3	ENSP00000352833	ENSP00000386834	0.548
PTPN22	AIRE	ENSP00000352833	ENSP00000291582	0.530
PTPN22	ANKRD55	ENSP00000352833	ENSP00000342295	0.520
PTPN22	BLK	ENSP00000352833	ENSP00000259089	0.761
PTPN22	CD226	ENSP00000352833	ENSP00000280200	0.475
PTPN22	CD28	ENSP00000352833	ENSP00000324890	0.408

PTPN22	CD40	ENSP00000352833	ENSP00000361359	0.570
PTPN22	CTLA4	ENSP00000352833	ENSP00000303939	0.756
PTPN22	FCGR2A	ENSP00000352833	ENSP00000271450	0.478
PTPN22	FCRL3	ENSP00000352833	ENSP00000357167	0.722
PTPN22	GPR29	ENSP00000352833	ENSP00000343952	0.485
PTPN22	IL2RA	ENSP00000352833	ENSP00000369293	0.720
PTPN22	IL2RB	ENSP00000352833	ENSP00000216223	0.730
PTPN22	IRAK1	ENSP00000352833	ENSP00000358997	0.478
PTPN22	IRF5	ENSP00000352833	ENSP00000349770	0.720
PTPN22	P2RY10	ENSP00000352833	ENSP00000171757	0.564
PTPN22	PADI4	ENSP00000352833	ENSP00000364597	0.718
PTPN22	PLD4	ENSP00000352833	ENSP00000376372	0.638
PTPN22	PTPRC	ENSP00000352833	ENSP00000411355	0.623
PTPN22	SH2B3	ENSP00000352833	ENSP00000345492	0.655
PTPN22	STAT4	ENSP00000352833	ENSP00000376134	0.788
PTPN22	TAGAP	ENSP00000352833	ENSP00000356033	0.609
PTPN22	TNFAIP3	ENSP00000352833	ENSP00000481570	0.719
PTPN22	TRAF1	ENSP00000352833	ENSP00000362994	0.732
PTPN22	TYK2	ENSP00000352833	ENSP00000431885	0.522
PTPN22	UBASH3A	ENSP00000352833	ENSP00000317327	0.671
PTPN22	WDFY4	ENSP00000352833	ENSP00000320563	0.703
PTPRC	AIRE	ENSP00000411355	ENSP00000291582	0.416
PTPRC	BLK	ENSP00000411355	ENSP00000259089	0.433
PTPRC	CCL21	ENSP00000411355	ENSP00000259607	0.548
PTPRC	CD2	ENSP00000411355	ENSP00000358490	0.915
PTPRC	CD226	ENSP00000411355	ENSP00000280200	0.415
PTPRC	CD244	ENSP00000411355	ENSP00000357012	0.736
PTPRC	CD28	ENSP00000411355	ENSP00000324890	0.860
PTPRC	CD40	ENSP00000411355	ENSP00000361359	0.736
PTPRC	CD83	ENSP00000411355	ENSP00000368450	0.563

PTPRC	CTLA4	ENSP00000411355	ENSP00000303939	0.790
PTPRC	EOMES	ENSP00000411355	ENSP00000388620	0.528
PTPRC	FCGR2A	ENSP00000411355	ENSP00000271450	0.839
PTPRC	FCGR2B	ENSP00000411355	ENSP00000351497	0.865
PTPRC	GATA3	ENSP00000411355	ENSP00000368632	0.577
PTPRC	GPR29	ENSP00000411355	ENSP00000343952	0.614
PTPRC	IL2RA	ENSP00000411355	ENSP00000369293	0.665
PTPRC	IL2RB	ENSP00000411355	ENSP00000216223	0.766
PTPRC	IL6R	ENSP00000411355	ENSP00000357470	0.457
PTPRC	IRF4	ENSP00000411355	ENSP00000370343	0.609
PTPRC	IRF8	ENSP00000411355	ENSP00000268638	0.631
PTPRC	P2RY10	ENSP00000411355	ENSP00000171757	0.446
PTPRC	PRDM1	ENSP00000411355	ENSP00000358092	0.523
PTPRC	PTPN22	ENSP00000411355	ENSP00000352833	0.623
PTPRC	REL	ENSP00000411355	ENSP00000295025	0.400
PTPRC	RUNX1	ENSP00000411355	ENSP00000300305	0.410
PTPRC	STAT4	ENSP00000411355	ENSP00000376134	0.443
PTPRC	TAGAP	ENSP00000411355	ENSP00000356033	0.620
PTPRC	TNFRSF14	ENSP00000411355	ENSP00000347948	0.430
PTPRC	TYK2	ENSP00000411355	ENSP00000431885	0.583
PTPRC	WDFY4	ENSP00000411355	ENSP00000320563	0.649
RAD51B	COG6	ENSP00000419471	ENSP00000397441	0.449
RASGRP1	CD28	ENSP00000310244	ENSP00000324890	0.434
RASGRP1	CTLA4	ENSP00000310244	ENSP00000303939	0.463
REL	CD28	ENSP00000295025	ENSP00000324890	0.564
REL	CD40	ENSP00000295025	ENSP00000361359	0.801
REL	CDK5RAP2	ENSP00000295025	ENSP00000343818	0.544
REL	CTLA4	ENSP00000295025	ENSP00000303939	0.559
REL	IL2RA	ENSP00000295025	ENSP00000369293	0.457

REL	IRAK1	ENSP00000295025	ENSP00000358997	0.655
REL	IRF4	ENSP00000295025	ENSP00000370343	0.732
REL	IRF5	ENSP00000295025	ENSP00000349770	0.415
REL	IRF8	ENSP00000295025	ENSP00000268638	0.442
REL	NFKBIE	ENSP00000295025	ENSP00000275015	0.992
REL	PTPRC	ENSP00000295025	ENSP00000411355	0.400
REL	STAT4	ENSP00000295025	ENSP00000376134	0.411
REL	TNFAIP3	ENSP00000295025	ENSP00000481570	0.649
REL	TRAF1	ENSP00000295025	ENSP00000362994	0.583
REL	TRAF6	ENSP00000295025	ENSP00000433623	0.597
RUNX1	BLK	ENSP00000300305	ENSP00000259089	0.902
RUNX1	CDK6	ENSP00000300305	ENSP00000265734	0.963
RUNX1	CTLA4	ENSP00000300305	ENSP00000303939	0.922
RUNX1	ETS1	ENSP00000300305	ENSP00000376436	0.974
RUNX1	GATA3	ENSP00000300305	ENSP00000368632	0.949
RUNX1	IL2RA	ENSP00000300305	ENSP00000369293	0.927
RUNX1	PTPRC	ENSP00000300305	ENSP00000411355	0.410
RUNX1	TRAF6	ENSP00000300305	ENSP00000433623	0.419
SH2B3	CD226	ENSP00000345492	ENSP00000280200	0.476
SH2B3	CTLA4	ENSP00000345492	ENSP00000303939	0.410
SH2B3	IL2RA	ENSP00000345492	ENSP00000369293	0.473
SH2B3	PTPN2	ENSP00000345492	ENSP00000311857	0.547
SH2B3	PTPN22	ENSP00000345492	ENSP00000352833	0.655
SH2B3	STAT4	ENSP00000345492	ENSP00000376134	0.400
SH2B3	TAGAP	ENSP00000345492	ENSP00000356033	0.658
SH2B3	TNFAIP3	ENSP00000345492	ENSP00000481570	0.421
SH2B3	TYK2	ENSP00000345492	ENSP00000431885	0.464
SH2B3	UBASH3A	ENSP00000345492	ENSP00000317327	0.543
SPRED2	AFF3	ENSP00000348753	ENSP00000386834	0.433
SPRED2	ANKRD55	ENSP00000348753	ENSP00000342295	0.475

SPRED2	IRF5	ENSP00000348753	ENSP00000349770	0.416
STAT4	AFF3	ENSP00000376134	ENSP00000386834	0.473
STAT4	ANKRD55	ENSP00000376134	ENSP00000342295	0.438
STAT4	BLK	ENSP00000376134	ENSP00000259089	0.707
STAT4	CD2	ENSP00000376134	ENSP00000358490	0.409
STAT4	CD226	ENSP00000376134	ENSP00000280200	0.426
STAT4	CD28	ENSP00000376134	ENSP00000324890	0.551
STAT4	CD40	ENSP00000376134	ENSP00000361359	0.533
STAT4	CTLA4	ENSP00000376134	ENSP00000303939	0.681
STAT4	EOMES	ENSP00000376134	ENSP00000388620	0.491
STAT4	ETS1	ENSP00000376134	ENSP00000376436	0.472
STAT4	FCGR2A	ENSP00000376134	ENSP00000271450	0.402
STAT4	FCRL3	ENSP00000376134	ENSP00000357167	0.463
STAT4	GATA3	ENSP00000376134	ENSP00000368632	0.819
STAT4	GPR29	ENSP00000376134	ENSP00000343952	0.479
STAT4	IKZF3	ENSP00000376134	ENSP00000344544	0.414
STAT4	IL2RA	ENSP00000376134	ENSP00000369293	0.569
STAT4	IL2RB	ENSP00000376134	ENSP00000216223	0.487
STAT4	IL6R	ENSP00000376134	ENSP00000357470	0.469
STAT4	IRAK1	ENSP00000376134	ENSP00000358997	0.476
STAT4	IRF4	ENSP00000376134	ENSP00000370343	0.534
STAT4	IRF5	ENSP00000376134	ENSP00000349770	0.737
STAT4	IRF8	ENSP00000376134	ENSP00000268638	0.516
STAT4	PADI4	ENSP00000376134	ENSP00000364597	0.575
STAT4	PRDM1	ENSP00000376134	ENSP00000358092	0.476
STAT4	PTPN2	ENSP00000376134	ENSP00000311857	0.791
STAT4	PTPN22	ENSP00000376134	ENSP00000352833	0.788
STAT4	PTPRC	ENSP00000376134	ENSP00000411355	0.443
STAT4	REL	ENSP00000376134	ENSP00000295025	0.411
STAT4	SH2B3	ENSP00000376134	ENSP00000345492	0.400

STAT4	TNFAIP3	ENSP00000376134	ENSP00000481570	0.678
STAT4	TRAF1	ENSP00000376134	ENSP00000362994	0.695
STAT4	TYK2	ENSP00000376134	ENSP00000431885	0.978
STAT4	UBE2L3	ENSP00000376134	ENSP00000400906	0.447
STAT4	WDFY4	ENSP00000376134	ENSP00000320563	0.506
TAGAP	ANKRD55	ENSP00000356033	ENSP00000342295	0.491
TAGAP	CD2	ENSP00000356033	ENSP00000358490	0.595
TAGAP	CTLA4	ENSP00000356033	ENSP00000303939	0.469
TAGAP	IL2RA	ENSP00000356033	ENSP00000369293	0.438
TAGAP	IRF8	ENSP00000356033	ENSP00000268638	0.442
TAGAP	PTPN2	ENSP00000356033	ENSP00000311857	0.543
TAGAP	PTPN22	ENSP00000356033	ENSP00000352833	0.609
TAGAP	PTPRC	ENSP00000356033	ENSP00000411355	0.620
TAGAP	SH2B3	ENSP00000356033	ENSP00000345492	0.658
TAGAP	TNFAIP3	ENSP00000356033	ENSP00000481570	0.539
TAGAP	TNFRSF14	ENSP00000356033	ENSP00000347948	0.493
TAGAP	TYK2	ENSP00000356033	ENSP00000431885	0.414
TAGAP	UBASH3A	ENSP00000356033	ENSP00000317327	0.461
TAGAP	WDFY4	ENSP00000356033	ENSP00000320563	0.608
TNFAIP3	AFF3	ENSP00000481570	ENSP00000386834	0.430
TNFAIP3	ANKRD55	ENSP00000481570	ENSP00000342295	0.502
TNFAIP3	BLK	ENSP00000481570	ENSP00000259089	0.623
TNFAIP3	CD40	ENSP00000481570	ENSP00000361359	0.634
TNFAIP3	CD83	ENSP00000481570	ENSP00000368450	0.444
TNFAIP3	CTLA4	ENSP00000481570	ENSP00000303939	0.461
TNFAIP3	ETS1	ENSP00000481570	ENSP00000376436	0.432
TNFAIP3	IL2RA	ENSP00000481570	ENSP00000369293	0.440
TNFAIP3	IRAK1	ENSP00000481570	ENSP00000358997	0.691
TNFAIP3	IRF4	ENSP00000481570	ENSP00000370343	0.467
TNFAIP3	IRF5	ENSP00000481570	ENSP00000349770	0.687

TNFAIP3	IRF8	ENSP00000481570	ENSP00000268638	0.480
TNFAIP3	NFKBIE	ENSP00000481570	ENSP00000275015	0.630
TNFAIP3	PADI4	ENSP00000481570	ENSP00000364597	0.473
TNFAIP3	PRDM1	ENSP00000481570	ENSP00000358092	0.555
TNFAIP3	PTPN22	ENSP00000481570	ENSP00000352833	0.719
TNFAIP3	REL	ENSP00000481570	ENSP00000295025	0.649
TNFAIP3	SH2B3	ENSP00000481570	ENSP00000345492	0.421
TNFAIP3	STAT4	ENSP00000481570	ENSP00000376134	0.678
TNFAIP3	TAGAP	ENSP00000481570	ENSP00000356033	0.539
TNFAIP3	TNFRSF14	ENSP00000481570	ENSP00000347948	0.520
TNFAIP3	TRAF1	ENSP00000481570	ENSP00000362994	0.983
TNFAIP3	TRAF6	ENSP00000481570	ENSP00000433623	0.977
TNFAIP3	TYK2	ENSP00000481570	ENSP00000431885	0.491
TNFAIP3	UBE2L3	ENSP00000481570	ENSP00000400906	0.606
TNFAIP3	WDFY4	ENSP00000481570	ENSP00000320563	0.516
TNFRSF14	CCL21	ENSP00000347948	ENSP00000259607	0.463
TNFRSF14	CD28	ENSP00000347948	ENSP00000324890	0.558
TNFRSF14	CTLA4	ENSP00000347948	ENSP00000303939	0.651
TNFRSF14	PTPRC	ENSP00000347948	ENSP00000411355	0.430
TNFRSF14	TAGAP	ENSP00000347948	ENSP00000356033	0.493
TNFRSF14	TNFAIP3	ENSP00000347948	ENSP00000481570	0.520
TNFRSF14	TRAF1	ENSP00000347948	ENSP00000362994	0.943
TRAF1	AFF3	ENSP00000362994	ENSP00000386834	0.457
TRAF1	BLK	ENSP00000362994	ENSP00000259089	0.468
TRAF1	C5	ENSP00000362994	ENSP00000223642	0.473
TRAF1	CD40	ENSP00000362994	ENSP00000361359	0.994
TRAF1	CTLA4	ENSP00000362994	ENSP00000303939	0.514
TRAF1	FCRL3	ENSP00000362994	ENSP00000357167	0.404
TRAF1	IL2RA	ENSP00000362994	ENSP00000369293	0.418
TRAF1	IL2RB	ENSP00000362994	ENSP00000216223	0.422

TRAF1	IRAK1	ENSP00000362994	ENSP00000358997	0.509
TRAF1	IRF5	ENSP00000362994	ENSP00000349770	0.490
TRAF1	NFKBIE	ENSP00000362994	ENSP00000275015	0.531
TRAF1	PADI4	ENSP00000362994	ENSP00000364597	0.558
TRAF1	PTPN22	ENSP00000362994	ENSP00000352833	0.732
TRAF1	REL	ENSP00000362994	ENSP00000295025	0.583
TRAF1	STAT4	ENSP00000362994	ENSP00000376134	0.695
TRAF1	TNFAIP3	ENSP00000362994	ENSP00000481570	0.983
TRAF1	TNFRSF14	ENSP00000362994	ENSP00000347948	0.943
TRAF1	TRAF6	ENSP00000362994	ENSP00000433623	0.723
TRAF1	UBE2L3	ENSP00000362994	ENSP00000400906	0.468
TRAF6	ATG5	ENSP00000433623	ENSP00000358072	0.489
TRAF6	CD28	ENSP00000433623	ENSP00000324890	0.434
TRAF6	CD40	ENSP00000433623	ENSP00000361359	0.994
TRAF6	CTLA4	ENSP00000433623	ENSP00000303939	0.453
TRAF6	IRAK1	ENSP00000433623	ENSP00000358997	0.999
TRAF6	IRF4	ENSP00000433623	ENSP00000370343	0.651
TRAF6	IRF5	ENSP00000433623	ENSP00000349770	0.951
TRAF6	IRF8	ENSP00000433623	ENSP00000268638	0.612
TRAF6	REL	ENSP00000433623	ENSP00000295025	0.597
TRAF6	RUNX1	ENSP00000433623	ENSP00000300305	0.419
TRAF6	TNFAIP3	ENSP00000433623	ENSP00000481570	0.977
TRAF6	TRAF1	ENSP00000433623	ENSP00000362994	0.723
TRAF6	UBE2L3	ENSP00000433623	ENSP00000400906	0.688
TYK2	CD40	ENSP00000431885	ENSP00000361359	0.417
TYK2	CTLA4	ENSP00000431885	ENSP00000303939	0.404
TYK2	IL2RA	ENSP00000431885	ENSP00000369293	0.768
TYK2	IL2RB	ENSP00000431885	ENSP00000216223	0.685
TYK2	IL6R	ENSP00000431885	ENSP00000357470	0.950
TYK2	IRF5	ENSP00000431885	ENSP00000349770	0.555

TYK2	IRF8	ENSP00000431885	ENSP00000268638	0.494
TYK2	PTPN2	ENSP00000431885	ENSP00000311857	0.901
TYK2	PTPN22	ENSP00000431885	ENSP00000352833	0.522
TYK2	PTPRC	ENSP00000431885	ENSP00000411355	0.583
TYK2	SH2B3	ENSP00000431885	ENSP00000345492	0.464
TYK2	STAT4	ENSP00000431885	ENSP00000376134	0.978
TYK2	TAGAP	ENSP00000431885	ENSP00000356033	0.414
TYK2	TNFAIP3	ENSP00000431885	ENSP00000481570	0.491
UBASH3A	COG6	ENSP00000317327	ENSP00000397441	0.446
UBASH3A	CTLA4	ENSP00000317327	ENSP00000303939	0.425
UBASH3A	IL2RA	ENSP00000317327	ENSP00000369293	0.499
UBASH3A	PTPN2	ENSP00000317327	ENSP00000311857	0.471
UBASH3A	PTPN22	ENSP00000317327	ENSP00000352833	0.671
UBASH3A	SH2B3	ENSP00000317327	ENSP00000345492	0.543
UBASH3A	TAGAP	ENSP00000317327	ENSP00000356033	0.461
UBASH3A	UBE2L3	ENSP00000317327	ENSP00000400906	0.434
UBASH3A	YDJC	ENSP00000317327	ENSP00000292778	0.415
UBE2L3	BLK	ENSP00000400906	ENSP00000259089	0.585
UBE2L3	IRF5	ENSP00000400906	ENSP00000349770	0.445
UBE2L3	STAT4	ENSP00000400906	ENSP00000376134	0.447
UBE2L3	TNFAIP3	ENSP00000400906	ENSP00000481570	0.606
UBE2L3	TRAF1	ENSP00000400906	ENSP00000362994	0.468
UBE2L3	TRAF6	ENSP00000400906	ENSP00000433623	0.688
UBE2L3	UBASH3A	ENSP00000400906	ENSP00000317327	0.434
UBE2L3	WDFY4	ENSP00000400906	ENSP00000320563	0.509
UBE2L3	YDJC	ENSP00000400906	ENSP00000292778	0.477
UTS2	AFF3	ENSP00000054668	ENSP00000386834	0.536
UTS2	P2RY10	ENSP00000054668	ENSP00000171757	0.902
WDFY4	ATG5	ENSP00000320563	ENSP00000358072	0.542
WDFY4	BLK	ENSP00000320563	ENSP00000259089	0.636

WDFY4	CD244	ENSP00000320563	ENSP00000357012	0.403
WDFY4	ETS1	ENSP00000320563	ENSP00000376436	0.440
WDFY4	IRF5	ENSP00000320563	ENSP00000349770	0.571
WDFY4	IRF8	ENSP00000320563	ENSP00000268638	0.597
WDFY4	PLD4	ENSP00000320563	ENSP00000376372	0.445
WDFY4	PTPN22	ENSP00000320563	ENSP00000352833	0.703
WDFY4	PTPRC	ENSP00000320563	ENSP00000411355	0.649
WDFY4	STAT4	ENSP00000320563	ENSP00000376134	0.506
WDFY4	TAGAP	ENSP00000320563	ENSP00000356033	0.608
WDFY4	TNFAIP3	ENSP00000320563	ENSP00000481570	0.516
WDFY4	UBE2L3	ENSP00000320563	ENSP00000400906	0.509
YDJC	UBASH3A	ENSP00000292778	ENSP00000317327	0.415
YDJC	UBE2L3	ENSP00000292778	ENSP00000400906	0.477

S1 Text:

PTPN22

rs3765598

CATTGGTATCTTAATATTTTCTGAACAGACAGTAAAATAAGGAATGAAAGGAATTTGAC

Y

CAGGACAAGGGATACAGGAAAAATATTGGGATATTCAGAGTTAGAGAAAATGGCAATGGA

rs3811021

AAAATCTAGTTCAACTCTAATTTTATGTAGTAAATAAATTGGCAGGTAAT

Y

GTTTTTACAAAGAATCCACCTGACTTCCCCTAATGCATTAAAAATATTTTTATTTAAATA

rs1217414

ATACAGCGGTGAACAAAAAGAACCAAAACCTTTGCCTCCTGGAGCTAACA

Y

TCTGGTACCGGATAGCAGATGTGAAAACACTCTGAAAGTCTTGAGTAATTAGATATTATT

FCRL3

rs3761959

AGAGCCTCCTCCGACTTTTTCAGTCTCTAGGTTTTTTTTTTTTTTTCTCCCTACATTACC

N

GTATTCCTCAGACTACAGGAAGGAAACACTTGCAGAGCAAAGGACAGAGGCAAGAGGCTG

CTLA4

rs231779

GCCTGGTTAGTTACAGGAAGGTAATTTGGCATGCAGCCACTATTTTTGAGTTGATGCAAG

Y

CTCTCTGTATGGAGAGCTGGTCTCCTTTATCCTGTGGGAAAAGAGAACAAAGGAGCATGG

rs231777

GCTTAAAATGATGAGTATATCCATTGAATCTCAACCTTATCTCTCTCTAGACCTTCTTGG

H

TAAGAAACCATGTAGTTTGTATGAAGTAGGTACTCAAAAGATATTTGATGATTTAATTTT

TRAF1/C5

rs3761847

TGGGATAAAACAGGCACCCAAGACTTCTCTCCCCATCTGTGGGTCCCTTCTCTCCCCTCC

R

GCCTCAATACCACCCTCTCTACCTGCTCATTCCCACGGACATCAAAACGTGCGCAACCTG

rs2900180

ggtggggtctgggaatttgcattcctgacaaactcccaggcggccagggaccacactttg

H

ATAGCATTGTTCTAAGGCTGACAGTCCTGAGGACCAAAAGAGGAAGGCAAATGGGAAAAT

TNFAIP3

rs5029930

CACCTGTAGAGGACGTCTGACATCAAAAGAGAGCACCATCTGATCCAGAGAATGACTCCC

M

CTATGCCTTTCCCAGCAATCAGATTAAGAATCCCAATCTAAAACTATCACCAGAAAACTC

rs5029937

AGATTAAGGAGTTAACTTTTTTTGTTTTAAACATTCCCCCAAAATATTTATCGTTTGGGG

K

TTGAAAAAAATGCAGCCCATTGAATTGTGAGTTTTAAACTGGAAAGGTCACAGATTAATG

rs5029939

AAAAATGCCCAGTGAACTTAAGGAATTTCCTCCAGGTCACCTAAACTAGTTAGGAGCAGA

S

TTAAGCTAGAACCAAGGTCCCCTGGCTCCTTTGCAGTTGGTGTCATTCATTAAAAAGAAG

STAT4

rs7574865

TATGGAAAATTACATGAGTGTGTATGCAGTAAAAGTATGAAAAGTTGGTGACCAAAATGT

K

AATAGTGGTTATCTTATTTCAGTGGAATTTCAGGGGATTTTTTTTCTTTCTTCTTAGACT

IL2-IL21

rs17388568

CACATTTTCTTATCTAAGTCATCGGATTAATTCCATGTCACCTTTGGAACATTTATCATA

R

TAACGCCAATGTCTACCCTGTGGATTTTAGTGGCTTCTATTATTAAGAACTGTGAACTCA

rs907715

TCTAAGTGAGCAATCCATATATGAAAAACTGTTCAATCTCCCTAGTAATCACATAAATGC

R

AGTTAAAACAAGGAAATCCTGTTTTTTCCAATTAAACATTTTAAACAATACCCTATAATA

IL2RB

rs743777

GTCAGGGCTTTGTGCATGCTGAGCCTGCTCTTCCCTTGCCATTTTACAGGCCAGGAAACC

R

GGCCTCATGAGTCATCCTACCAGAATGTGGCAGAGCCAGGATTAGAACCTAGGTCTGTCC

rs3218253

GCAGCAGGGGCCCCGAACTGCACCTGACCAGGTTCAAATCCCAGCTCTATGCCCTTCTCG

Y

CTGGGTGAGAGGTTGACCCGTTTCTTAGTCTCCTCATTTGGCAAAAAGGAGGCATACTAG

IL2RA

rs2104286

AGAGAGTCATAAGTTGGTGAGGAGGAGAAAGGCATAGATATAGTCATGGTAACACAAGTC

R

TATGTGGTAAGATCTACTGAGCATGGGGCTATCAGAGAAAGCTTCATGAGGGTGACATGG

CD40

rs4810485

ACCTGGCTCCTTCATCCCAGCCCCTCTGGCCTCCCCCTACTTTAGAGGGCTGTAGATTCC

D

GCCTGAAGCCTGGGCAGGAATGACCCATGGTATCAAGGAAAGCAAGGGAAGCAGCAAGGG

rs1535045

CACAGCTTTCACTTTCACCTGAAGCAATGGCTCTTAGGGAACAGGGAGGCAGGGGGAGGG

N

GGAGCTGGAAAGAGGTAAAGGGGGGCCCTTGTGGTAGGAGTGGAGAAAGAGCCAGAGGAG

rs3765459

TCGTCGCCCTTGGTGTGGCCAGCAGGGGGCAGGAGGCACCCGAGGAATCAGCACTGACCC

V

CCGTCTGGGAAAGGGGGGAGGGCTTGGGGAAGGGATCCGCTTCCCAGGGAGGGGCTCCTC

AFF3

rs10865035

CAATTGTTGTCTCATATTAAAGGGTATCAGCTGTGATTTAAAACCTCTATCTGGGGAAAA

R

TAGACAAATTCCTTCCACATCAGGGTAGTTGAAAGAATCCATGATTTGGAATTTAGAGAC

rs1160542

CTGTTTTCAGGTCACGTTCCTCTAGCGCAGCAGATCCCACCTGGCTACACGCTGGAATAA

R

CAAGAGACTTCCAGAAACACAAATGCCTGGGCTGTACCCCAGAGAGATTCCATCAGAGTC

rs9653442

CACACAGTCCTGGCCCATCGGGCTCTCTGGAGGCCCTTCTTCCTTGGCTGTCACCTAttt

Y

taaactgatatgtaatagttgtacatatttatggactatatgtaatattttgatacatgc

CD244

rs3766379

GACAGGCAGAGTTGGCCCCTGCCTGCTGCGAAGAGCAGGCGAGAGGCTCCTGGAGGAGCC

Y

ACTCAGGCTGGTGGGCTTTGTGCCAGGCTGGTTACTCTCTCTAGTACCTCAGCCACACTC

rs6682654

TTGTGGCTGATTGCCTCGTGCTCTGAGAACTCACGGTCAAGGTTTTCTCTTTGCTCATTT

R

GTTAACCTCCTCTGAGTGATGCCAAGAGCATAGATATATCAGACAGGCCTGGCTCAAATT

TRAF6

rs540386

AAAGGAAGCAAACAATGGCAAGACAGCAGAAAATAGCAGAACTAGTCACTACAGCTGGGT

B

GTAAAATACGGTATAGGGCCCCCTTTATACCTTCCAAATCTGAGGACAAATGATGCTGTT

REL

rs702873

TCCTTAAGCTTTCCTGGGCTTTGGGCCCTGTGCGGGTTTTGAGGGTGGTGGTAGACGTAC

R

CATCATCTACCTTCCACTGAGTTGGAACCAACTGAGAAGTAGAATGAGTCCACAGCTAGG

rs13031237

ATACAAGAAAAAGCCTTTCCTTACAACTGAAAACTTAAAGTTTGAAAAAATGGCTCATGT

K

TACTTCATTGTCCTTTCTTTATTGCAGTCAGAGTTTTCGAAGCCTTTTTTTCATTACCCT

CD28

rs2140148

GAACTCATTCAATTCAGGTAGATAGAGTAATTACAGTTCACATTTATCTTGAAGCCTGAC

D

GAGCTAAGCCCTTCGCATGGATTATTTCATTTTACCTCACAATGACCTTGAGGTGAGGTT

BLK

rs2248932

TTACTTCCCAATGGAGTCAGTTATGATTCTTTGGCACAATAGTTCTGAAACCTCAGTTTG

Y

ATTTTAACCAGCAGGGGAGTTTGCAGACTCCCAACCCCAGCCCCAGCAGTGCTGGTATCT

PTPRC

rs10919563

AAGAAAAATAATATTGTATTATAAATAAATAATTATAGTAATTGCTATAAAATGCATATA

R

TCAAACGTGAATGTCCTTATAATGGTGATGTTTGGTCTGGGATCCCTTCTCTTAAGCATA

CD2

rs11586238

tgaacctcttttgcttggttcccattgccttcagcata

S

agttcaaacttcctagtataacaaacaagtctgttcatgatctgggccccacaggcctgt

rs624988

TCTTTCTCCCTCCACTAGAATGTAAGTTCCATGAAGGCTGCCATCTGGTC

D

GGGTGTTTCCACATGCCCCCACCACTTAGTATGGTGGCTGTCACAGTGTGGGGGTGCCAT

rs798036

CCTACCCACTCCAGCAGCTGCAGCTGGCGCCTAAATGTGTGCTCCCAGGA

W

TTCCTCCAGGCTCATTGTCCTTCTCCCTTGTTTGCATCTCCTGGTTACTC

SPRED2

rs871974

ATCTCTCAACCAGACTACTTTTCACAGTCTGTAAGCCTCTCATGGGTCAG

Y

CTCATTTCTGCAAACCATCTTGTGATCTCTTTGAGGGTTGGAGCCATGGCTCACATACCT

IRF5

rs2004640

TGGAGTAGGGCGGGGTCCGCGTCCAGCTGCGCCTGGAAAGCGAGCTCGGG

K

GGGTGCCTACAGCAGGGTGCGCCCGGCCGGCCTGGGACTTCCAAAGCGCCTCCCACGCCC

rs2070197

AGCACCTGGCTGGCTGCAGGGTCCTACCTCTGGGTTTCCTGGAAGTGGAT

Y

TGGGCCAAGAAGGAGAGGGAGAAAGGCCCGAGCCCCTGCCTTCCCGGGCCTTTCTCTCCT

rs10954213

GAGTCCAAGAACCTGGAGCAGAAATAATTTTTATGTATTTTTGGATTAAT

R

AATGTTAAAAACAGACTCAGCTGTTTCTTTCCTTTTACTACTACCAGTTGCTCCCATGCT

CCR6

rs3093023

GCCACATTCCTCGCCTTTTATGCACCTCACAGTGTCTATGCAAATGAACA

R

TGTGATTTTAAATTTGAGGAAGTTTCAATAAGAGTGAGATCTAAGAGGATCTTTAATATA

ANKRD55

rs926657

AACTGGTTGCCCTGGTAACAGTGTGGTTGTTTATCGGTTG

V

TTTTTCTAGATTAAGATTCACGCCCTTTGCTTTCTCAAGTTTTCAAAATTATTTTTCTCA

rs9295089

GAACCTAGATCTGGCAACTGGGAATTATCAGCAAAACGTTATCAAGATATTCTGTCCTTC

Y

GAAATCCACAAGATGTGTGTGCAATGGATATTGCAATCTCAAACATTTCCTTCTTTCTTT

rs212402

TCTGTAAGAAAAGCCCAGGAGGCAGCATCAGGTTTGCAAAAATTACGTGGCTTTCCCTTG

Y

CCCTCTGCCCAAAGTCTTTTCACACAGAAGATGACACTAGCTTTAAACCCTTCCCCCGGA

IL6R

rs4537545

CCCCCTTACTGGTGATGGCATACTCCAGGAATTCTGGAAACCCTCCCTGA

Y

GCCGAAGCAGGGTCACCCTCTCCCCGCCTCCTTGCTAGTCCTTACTCCTGGCCCAGCGAG

rs4845617

CGCCGCTCTGAGTCATGTGCGAGTGGGAAGTCGCACTGACACTGAGCCGG

V

CCAGAGGGAGAGGAGCCGAGCGCGGCGCGGGGCCGAGGGACTCGCAGTGTGTGTAGAGAG

rs4329505|allelePos=501|totalLen=1001|taxid=9606|snpclass=1|alleles='C/T'|mol=Genomic|build=151

ATTTCGTTTTGAATGTAGTGTATCATTCCGGTATTTTAAAATCAGAGCTT

Y

TGCTACCTCCACAGCTGGGGGACGGGCAGAGGGACTTTCATGGTGTTCCGGGCCATGGAG

GATA3

rs569421|allelePos=501|totalLen=1001|taxid=9606|snpclass=1|alleles='C/T'|mol=Genomic|build=151

ATTCACTGAAGACCACCCAGCCCATATATAGCTAGCTCTTCTGTGAGATG

Y

GTATTTAACCCTGAAAAAACTAATGCAAGAAATAGGAGAGATGACCGAAGGGAAGACTCA

rs444929|allelePos=501|totalLen=1001|taxid=9606|snpclass=1|alleles='C/G/T'|mol=Genomic|build=151

AGTTAAAATTTTCTTTTTATGATGGAAGGTGAGGAGGAGAGAGAGGTTTA

B

ATTAGAAGTGACCCAACTCCATTTTCTTCCAATGGTTTTTTTCAGTTTTATTTTTTTAAA

rs3802604|allelePos=501|totalLen=1001|taxid=9606|snpclass=1|alleles='C/T'|mol=Genomic|build=151

GCCTCTTGCTTTCTTTTTGACTCCACCAGCTAGCCTGCCTCCCTCTCCCT

Y

CTGCCTACTTTCATGTACTGAACATGCAGGTCTGGGTTCTTCTGTCAGCCTAACCGCATC

ARID5B

rs10821944|allelePos=501|totalLen=1001|taxid=9606|snpclass=1|alleles='G/T'|mol=Genomic|build=151

TTTGTGATTCATTTGTGCTTTATTTTTATTGCAACAAATCTCACAGTAAA

K

AAACCTCTTTATCTTGGCCATAGAATTCAGTGACATTACTTTGGGCAGATTTATAATGGG

ARAP1

rs3781913|allelePos=501|totalLen=1001|taxid=9606|snpclass=1|alleles='A/C'|mol=Genomic|build=151

TCACCGTTGCATATCTCTTGATAACACTCTGACCCCTCCACTGCCAAATCTGATAAAAGA

M

CTCCCTTTGAAGACCTTCCTCCTGGAGTCGGATCTCAGTCCTTCTTGCTGTCCAGAGCCT

AIRE

rs2075876|allelePos=323|totalLen=716|taxid=9606|snpclass=1|alleles='A/G'|mol=Genomic|build=151

tccttccttgtgtctcccagctgctggtcattatgggggtacccctgtgctccttgttcctg

R

gctcaggacccaccgctccagcctctgcttctgtggtctcacagctgtctcccacgtgtc

rs933150|allelePos=501|totalLen=1001|taxid=9606|snpclass=1|alleles='A/G'|mol=Genomic|build=151

GCATTGATAACGGCCCCGGAAGATGTGTTCCTTGTTCTGCTGCTGTGAGG

R

TAGTAGGTCTACTGTGCACAGACCCAGTGTTCCCTCTGACAGCCCTGAGGGCCAGGGGGC

rs760426|allelePos=501|totalLen=1001|taxid=9606|snpclass=1|alleles='A/G'|mol=Genomic|build=151

GAGTGGAGGAGCTGGGATGTGGCTGTTTGGGGCCACAAATGGGGAATTCCACAGGGTTCA

R

TGTAATATGGTCTCCTCTCTGCTGGGGGTGCCTGCCTGGGGACCTTCTCCCACTCTGGTC

ANXA3

rs2867461|allelePos=501|totalLen=1001|taxid=9606|snpclass=1|alleles='A/G/T'|mol=Genomic|build=151

TTTGTTAGCTTCTAAATTAAATAGGAAGCATGATACAGAAGAATGATCTTTAGAGATGGG

D

ATTAAATTGAGTATCAGTCTCAGGATTAAAAAAAAGAAAATGATTGCTGCAATTTCAGCC

TNFRSF14

rs3890745|allelePos=501|totalLen=1001|taxid=9606|snpclass=1|alleles='A/G'|mol=Genomic|build=151

GTGTTGACCAAATTTGCTTGATAATTGGAATCACCTGGGGAAATTGTTACAAATCCAGAC

R

TTGGGCCCTCCCTCAGTTTGCCTGGGCTACAGTCACAAAATGCTCAGGTGGCTTAAAAAC

rs6684865|allelePos=1396|totalLen=3384|taxid=9606|snpclass=1|alleles='A/G'|mol=Genomic|build=151

TGTTTTTCAGACACCACATTATCCTAGAAGCTGTTGGTTTTTGATACCATTATCC

R

ATAAAAGAAGCCAGGGCTCCTTAGAAAAATGGTTGATTATACAACTGGGGTAGGAAACAT

RUNX1

rs2268277|allelePos=501|totalLen=1001|taxid=9606|snpclass=1|alleles='A/C/G'|mol=Genomic|build=151

GGATTTCCTAGAGAGGCACCTGTGATATCTCTGGCTAAACAGATACAGTTAAGAGCTGTC

V

CTGGGGCAGATCATGTTAAGTAACATGGCTGAGATTTGCCAGGGTCCATGTAATGCCTTA

RASGRP1

rs8043085|allelePos=501|totalLen=1001|taxid=9606|snpclass=1|alleles='G/T'|mol=Genomic|build=151

GTCAGGAGGGAAGTTGGTCCTCTTCAGTTCTGGAAATTGGACACAAGTCT

K

TGGGTGGGGTTTGGGGACTGGGGCTAGGAAATAAACAGAAGAGCAGGGAAGAGGTACTGC

PYPN2

rs2847297|allelePos=501|totalLen=1001|taxid=9606|snpclass=1|alleles='A/G'|mol=Genomic|build=151

TAAAATCTTTTCTTCCTTAGTGGGGCCTAAAGAAATTATGTCTGTGTAATGACTTGCCAA

R

TGGGACCTGCCCAGGTTTTCCCGTATATTTATGCAATTTAGGATATTAACCAATGGGAAT

ILF3

rs147622113|allelePos=51|totalLen=101|taxid=9606|snpclass=1|alleles='C/T'|mol=Genomic|build=151

TACTAAAAATACAAAAATTAGCCAGGAATGGTGGTCTGTGGCTGTAATCC

Y

AGTTACTCAGTAGAGTGAGGCAGAAGAATTGCTTGAACCCACGAGGCGGA

FADS2

rs968567|allelePos=301|totalLen=601|taxid=9606|snpclass=1|alleles='A/G'|mol=Genomic|build=151

CCTCCGCCCCTCCCTCAGCCTCCCGCTATGGACTTTTGCCTCCAGTAAAAACTCCCCGG

R

AGCTCAGGGCCTCGACACCTCCTCTGCCCGCCCCTCCGGCTCCCCCCGCCTCGGGTTCCA

rs968567|allelePos=301|totalLen=601|taxid=9606|snpclass=1|alleles='A/G'|mol=Genomic|build=151

CCTCCGCCCCTCCCTCAGCCCTCCCGCTATGGACTTTTGCCTCCAGTAAAAACTCCCCGG

R

AGCTCAGGGCCTCGACACCTCCTCTGCCCGCCCCTCCGGCTCCCCCCGCCTCGGGTTCCA

FADS3

rs76599700|allelePos=251|totalLen=501|taxid=9606|snpclass=1|alleles='C/T'|mol=Genomic|build=151

AGGTGTCATGCAGGGTGTGTTGGGGAAGACCCTGCCAGGGACACAGATGCCTGGCAGAGT

Y

AGCCCAGCAACTCCTCCTGGGAAGACACCCTCACCGAGAGAGACCCACACCCCCCCTGTT

CDK5RAP2

rs12379034|allelePos=501|totalLen=1001|taxid=9606|snpclass=1|alleles='A/G'|mol=Genomic|build=151

GCACCTAAAATAAAAGAGATGATGAGTAGAAAAGAATGAGCTAGTCCATGAGGACAAATT

R

GGACTTAGAGCCTATAAAATAAAAGAATAAACATTGGGTTCCAATATTTATCCTTTCAAA

JAZF1

rs864745|allelePos=501|totalLen=1001|taxid=9606|snpclass=1|alleles='A/G'|mol=Genomic|build=151

AGCTGTAAAGTTCTTTCTGCGTTAAAACATTGAACATTTCCTACAACCATTCAAAACATT

R

TAACAGTTCAAATTATATTTGAGCATCACTTATATGGCTCTTACGGAACTTATGTAAAGT

ETV7

rs879036|allelePos=501|totalLen=1001|taxid=9606|snpclass=1|alleles='C/T'|mol=Genomic|build=151

TAATCTCCATCCTCCCATTCCACCCCACCTCTCTGGTGTTTTACACCCACATCCATCTAA

Y

CTCCTGCTTCTCATAGGCCATGATCTAGGGCAGCATTTCTCAAACCATTTTTGACCTCAA

ETS1

rs1128334|allelePos=501|totalLen=1001|taxid=9606|snpclass=1|alleles='A/G'|mol=Genomic|build=151

TAAGCTGAGAAGTATAGGGATGAAAAAGATGTTATATTGTGTTTGACTATTTTCCAACTT

R

TATTTTCATATAATTTATATTTTTTAAAAGCTGAAAATTTAGAAGCAAGATGAAAAAAAG

COG6

rs9603612|allelePos=501|totalLen=1001|taxid=9606|snpclass=1|alleles='C/G'|mol=Genomic|build=151

ATTTACCAAAAGACTCTCTGCAAGTGGTAAATCATTAGCTCTAGTGTTGCTCTTTGTAAC

S

TCAGGTCTTTGGGGAATGGTGCAGAATTAGTATTGCTTCCTTCTTTCTGTGTGTGATAAT

rs7993214|allelePos=501|totalLen=1001|taxid=9606|snpclass=1|alleles='C/G/T'|mol=Genomic|build=151

TGGTCTGGTCTTTAGTGACTCAGGTTATAGGGAATTTACCACTTCTGTCGAAGCTGCTTT

B

GTAGTGGGGACTATCTAGCTGCTAGGAAAGTTTGTCAAATTCCCCTTGCTTTTGTTTAGT

CDK6

rs42041|allelePos=501|totalLen=1001|taxid=9606|snpclass=1|alleles='C/G'|mol=Genomic|build=151

ACCTGTGCTTCACCCACGGGCAGAAGCAGATATCAAAGGCTGTTAATTCAGGAGGTAAAG

S

AGACACTCATTACTCACTGGGTTTCAGACATCAAGAACAGCCCAACAGGTACACTTTTCT

UBE2L3

rs5754217|allelePos=1583|totalLen=1783|taxid=9606|snpclass=1|alleles='G/T'|mol=Genomic|build=151

GGAACCAAGTCCAGgtcTTTACTGGATCTCTAAAATATTTCATTCTGTGACTTGGGGTGT

K

GTTTTGCTATCCAGGATCCTTAGAGGTTTGGTCTTTAAGCCTGTATTCGTTCCTTCCCTG

UBASH3A

rs11203203|allelePos=501|totalLen=1001|taxid=9606|snpclass=1|alleles='A/G'|mol=Genomic|build=151

TAAAGACACTGTGTGTTTTATAGTTTTGTTGCCTAAAGAGTTTTCTTCTTGAAACTCTAC

R

ATTAAACAAAAAGCCGTTGAACTGGAGCTCGGGCCTGCAAGTCCAGGCCACTGGGTAACG

rs3788013|allelePos=501|totalLen=1001|taxid=9606|snpclass=1|alleles='A/C'|mol=Genomic|build=151

GCAGAGGTCAGAGTACACATGGTGAAAAAAGAGAAAAGCTGCTCAGCCTCATGGGTGTGC

M

TGTTGGGGTGGAGCTCTTGCAGGTGTCAAGACTGATGGTTGGAACAACTGAATGATGAGA

TEC

rs4694890|allelePos=501|totalLen=1001|taxid=9606|snpclass=1|alleles='A/C'|mol=Genomic|build=151

TTATTTGCCAGCTTCCATGCTGGGGCAGTACAACAGACAAAAAGCCAACCAAATTAAGAG

M

TCCAAAGTCCAATTCTAAAAATTCCTGATTTCTTAGCTAATGATAGTTGCAAATTTACAT

rs2089510|allelePos=501|totalLen=1001|taxid=9606|snpclass=1|alleles='A/G/T'|mol=Genomic|build=151

TTTCAAACTTTTCTCATATTTATTCATCTCAACAATTTTAGTAGTTCAGGGATGGAATGC

D

TTCTGTTCTACTTGTATTATCATTCTTAAAAGCAACCCAAAACTTGCAGAGTGAATCTCT

SYNGR1

rs909685|allelePos=501|totalLen=1001|taxid=9606|snpclass=1|alleles='A/T'|mol=Genomic|build=151

GTCTGCCCACCCCTGGGCCTCTGGCCTGGAAGGGCGAAGCCACTGGCTTTGTGAGGGGGC

W

TGTCTGCTTGGGTCATTTCTGCCTCTGATGCCTTCATTTAGCAAAGCTTTATTGAATCTG

RAD51B

rs3784099|allelePos=501|totalLen=1001|taxid=9606|snpclass=1|alleles='A/G'|mol=Genomic|build=151

TAAATCAAAGGGAGGGGAATCCCCAGAGCCCAGGAGTACACATGAATAGAAAGCACCACA

R

GTAATAAAAACACCTAAAAAGGTCAGCAGAAACTCGGGGAGCTGCTGGAAATTCACCTCC

rs911263|allelePos=501|totalLen=1001|taxid=9606|snpclass=1|alleles='A/G'|mol=Genomic|build=151

AGCAAATACCTTGAGTTAAGAGAGAATAAGCAGAATTAACTGGTCATTAGTCAGAGGATT

R

AGCCAACTGAGCCCAACTCTCTGCCAGTCTATGTGCCCAATGCAGACCCTCTGCCTTCAG

PRKCH

rs912620|allelePos=501|totalLen=1001|taxid=9606|snpclass=1|alleles='G/T'|mol=Genomic|build=151

TATCTCTTATGCATTTTGAAGGTAAAATTACAATTTTGTTCCATGTGAAATGGGAGCATG

K

GTTGACAAACCGTTGATTAACTTGAGTGGATTTGGGTTAATCTTTTTACTCTGCTCCATG

rs959728|allelePos=501|totalLen=1001|taxid=9606|snpclass=1|alleles='C/T'|mol=Genomic|build=151

AAGAAGTTCCCCTGCCTTTTATTTTGAGATAAGCCTCTCCACCTAAATGTGTGTTCCTTA

Y

TTCTGGACCTGTTGGCCAACGTACCTTTAGGGAGTGCCCCTCCAGCTGGCTTCAACATGT

rs3783782|allelePos=501|totalLen=1001|taxid=9606|snpclass=1|alleles='C/T'|mol=Genomic|build=151

AGATTTTTGATTTTTTTTTTTTTTAAGACAGAGTCTCGCTCTGTTGCCCAGGCTGGAGTG

Y

AATGGCGTGATCTTGGTTCACTGCAACCTTTGCCTCCCGGGTGCAAGCGATTCTCCCTGC

PPIL4

rs9498368|allelePos=501|totalLen=1001|taxid=9606|snpclass=1|alleles='A/G'|mol=Genomic|build=151

TCTCAACCTCAAAGGTGGATTTTGAGGAAGACTGACATGGGCTGGAATGGTGGTTTTCCA

R

AGAAAGGTTATGAGGAAGCCCAACAGGCAGAGCTATGCTAATTACATGTGGGACCACAGT

PLCL2

rs4535211|allelePos=501|totalLen=1001|taxid=9606|snpclass=1|alleles='A/G'|mol=Genomic|build=151

TTGAGCACAAGGCACACATAAATTTCATGCTGTCTGGGATGGGATAAGGATGTCGTGTAA

R

CATATCATTAATGTAGCTTAGAAAAGCTGAAAAGTGGCTGGGAGAGGATGGGGTTTGTGT

P2RY10

rs10465436|allelePos=1916|totalLen=3916|taxid=9606|snpclass=1|alleles='A/G'|mol=Genomic|build=151

ttttatgatattgattcttcctatccatgaccatggaatgtttttccatttcttt

R

tgtcctcccttatctccttgagcagtggtttgtagttcttcttgaagagctgcttcacat

MTF1

rs67704103|allelePos=501|totalLen=1001|taxid=9606|snpclass=1|alleles='C/T'|mol=Genomic|build=151

CAACATGCATTTGAACTGCACAAGTGGACTTATACTTGGGACTTTTTTCCACTTCTGCCA

Y

CCCTGACACAGCAAAACCAACCCCTCCTCTTCCTCTTCCTCCGCCTACTCAATGTGAAGA
