# Supplementary figures and images for: Characterization of Rheumatoid Arthritis Risk-Associated SNPs and Identification of Novel Therapeutic Sites Using an In-Silico Approach
Source: Biology (Basel). 2021 Jun 4;10(6):501. doi: 10.3390/biology10060501 (PMC8227790; doi:10.3390/biology10060501)

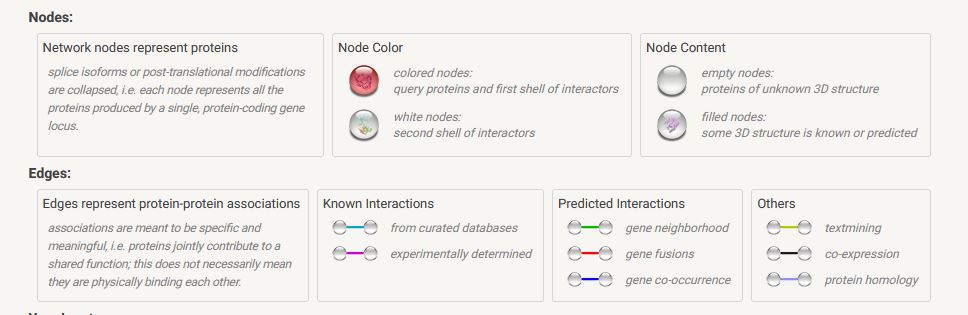

Supplement: Supplementary file 1 [file biology-10-00501-s001.zip › S2 Fig.JPG]
